# Beyond synaptic plasticity: a summary of a linear model of the cerebellar locomotor computation

**DOI:** 10.3389/fncir.2026.1815319

**Published:** 2026-05-12

**Authors:** Mike Gilbert, Anders Rasmussen

**Affiliations:** 1 School of Psychology, College of Life and Environmental Sciences, University of Birmingham, Birmingham, United Kingdom; 2 Department of Experimental Medical Science, Lund University, Lund, Sweden

**Keywords:** cerebellum, hypothesis, motor control, neural networks, neuroscience, theory, linear model, cerebellar computation

## Abstract

We present a summary of ideas that attempt to explain how the locomotor cerebellum may represent and process information. It includes the proposals that (i) the main network computation is a passive and unlearned effect of cell type morphologies and neural architecture; (ii) information has topographically defined spatial dimensions; (iii) it is coded at collective level, at any instant, in any random sample of functionally grouped signals; (iv) topographical organization extends outside the cerebellum and maps to the peripheral nervous system; and (v) learning and memory are at microzone level and in a supplementary role. The aim is to provide competition for traditional learning models and to challenge some common assumptions. We have found that the main resistance to the proposals is in these areas: loyalty to the traditional model; mathematically, the computation is unexpectedly unsophisticated; on the face of it, the mechanism is resource-heavy; we propose that neuroanatomy automates motor coordination and converts feedback into motor output in real time. Some of the ideas are contentious. We argue, nonetheless, that the proposals can explain the evidence.

## 1 Introduction

The cerebellum contains more than half the neurons in the vertebrate brain. It is essential for the coordinated and skilled execution of movement, yet the mechanistic principles through which it transforms input to output remain incompletely understood. The transformation is commonly thought to depend on algorithmically controlled synaptic weights that are tuned through learning [starting with the Marr/Albus (Albus, 1989) and adaptive filter (Fujita, 1982) models]. This idea has been a strong influence on neural network modeling generally, which, in turn, has influenced current thinking. In fact, it is one of several related assumptions, often implicit, which are mutually reinforcing and may be unsafe (Table 1). These include:

- The information coded in neural signals activity depends on which cells fire and what rate they each fire at
- Replicability—a repeat of the same information requires that there is a repeat of the same pattern of active cells
- Computations are sophisticated—linear functions are underpowered to perform sophisticated motor control—complex neural networks likely use non-linear algorithms, as AI does
- Biological units of the same type have standard and reliable performance characteristics
- The role of sensory feedback, including proprioception, is limited to performance refinement rather than directly contributing real-time drive

**Table 1:** A challenge to widely made assumptions.

Current thinking	Hypothesis
Code and replicability	
The information coded in neural signal activity depends on which cells fire and at what rates they each fire. Replicability: a repeat of the same information requires that there is a repeat of the same pattern of active cells.	Information is coded in the collective activity of topographically defined groups of computational nodes (‘code groups’). A node is any site where signals converge. Physically, some nodes are a whole cell, some are part of a cell, and some are some other structure. At each moment, information is encoded in the frequency distribution of the concurrent outputs of the computations of a code group. Code groups communicate at the group level, group to group. It makes no difference to information coded in this way which individual cells are active, or which cells fire at what rates; only the distribution across the group is functional.
Data processing	
Computations are sophisticated –powerful computations are necessary to perform complex motor tasks. Linear functions are underpowered. Complex neural networks likely use non-linear algorithms, as AI does,	Single unit computations are a linear function of the mean (and of the sum) of inputs. The function depends on the unit. Linear functions and random sampling together have the effect that, at any given time, outputs of a code group are statistically and predictably related to its inputs. This is a passive, unlearned effect of neuroanatomy.
Modular organization	
The cerebellum is organized functionally into circuits (sometimes called microcircuits). Circuits provide the mechanism that implements the cerebellar computation.	The smallest functional unit of the cerebellum is much larger: a ‘network’. Each network includes a circuit. Networks perform the main cerebellar computation. A single network contains millions of cells, mostly granule cells. Networks overlap—neighboring networks contain most of the same granule cells.
Learning and memory	
Memory is stored as finely graduated changes of parallel fiber synaptic transmission strength (termed synaptic weights). The pattern of trained synapses and permutation of individual weights is critical—otherwise, weights would not code pattern memory. Memory can be stored by a single Purkinje cell.	Training teaches synaptic weights to polarize, so a synapse either transmits robustly or transmits very weakly or not at all (‘silent’ synapses). Memory is stored as the ratio of transmitting to silent synapses. Functionally, it is immaterial which individual synapses transmit and which are silent. The smallest unit of learning and memory is a microzone. The compound effect of learned synaptic weight changes is a gain adjustment to the passive, anatomically mediated network computation.
Noise	
Models often assume biological units of the same type have standard and reliable performance characteristics. They also assume that patterns are repeated: a repeat of the same information is represented by a repeat of the same pattern of active cells.	Behavior of biological components—neurons and synapses—is randomly inconsistent. In fact, this is well-established: the behavior of single neurons is often unpredictable (Faisal et al., 2008) and synaptic transmission is noisy because it includes random variables (Rusakov et al., 2020)—Biology is messy. Patterns (in which cells are active) are not reliably repeated and perhaps are never repeated. In the cerebellum, this is in part messiness, but it is also an ‘intended’ function of the granular layer.
Sensory feedback	
The role of sensory feedback is limited to performance refinement.	‘Sensory’ feedback does not include proprioception (signals generated internally in muscles and joints), which should be considered a functionally different category. Proprioception-driven analogue cerebello-spinal signals supplement motor control. They contribute coordination and skilled execution of movement. They may also propagate or help propagate motor sequences.

Some new proposals (hypothesis column) and assumptions they challenge (current thinking column), which normally escape explicit scrutiny and may not be safe.

We propose a reinterpretation of the evidence. Our scope is the locomotor cerebellum, and our approach is bottom-up but bridging cellular, network, and systems levels. We put forward a candidate to explain the evidence without making the usual assumptions. In essence, we argue that modular cerebellar networks are the tangible form of a topographically organized network of linear functions, and that the network computation is primarily a passive effect of cell morphologies, anatomical architecture, topography, and specialized synaptic (and other) adaptations. With this simple proposition, we attempt to explain how input to the cerebellum is coded and organized, how it is processed and recoded through several internal steps, how that is reconciled with evidence from learning studies, how automated data sharing between cerebellar networks coordinates motor output, and how mapping to the peripheral nervous system may coordinate and propagate motor sequences.

Speaking generally, linear systems tend to be unpopular with modelers because linear functions limit computational flexibility. Some researchers regard them as simplistic. However, several lines of evidence suggest that cerebellar communication operates in a linear regime. For example, high-fidelity transmission has been reported between cerebellar cortical neurons (Rancz et al., 2007; Ritzau-Jost et al., 2014; Duguid et al., 2015; Duguid et al., 2012; Walter and Khodakhah, 2006; Walter and Khodakhah, 2009; Jelitai et al., 2016; Park et al., 2012), and some synapses are highly adapted for faithful transmission of rate information (Delvendahl and Hallermann, 2016; Saviane and Silver, 2006; Cesana et al., 2010; Moore and Blazis, 1989; Sargent et al., 2005; Turecek et al., 2016; Turecek et al., 2017). Additionally, firing of cerebellar cells has been shown to correlate linearly with behavioral metrics, reported ‘at all levels of the cerebellar circuit’ (Raymond and Medina, 2018, p. 239, citing 30 references). Furthermore, neurons are adapted to mitigate biophysical noise. ‘Although cerebellar neurons and synapses have nonlinear intrinsic properties, these properties are disengaged or compensated in ways that keep the cerebellar network operating in a linear regime’ (Raymond and Medina, 2018).

Most of the main text is a description of the network computation, a step at a time, with evidence. The main structure of the paper is provided by discussing cell types in turn, following the main direction of information flow. Some of the ideas are discussed separately in Boxes 1 to 5. To put the proposals in an example of functional context, in the final section, we incorporate the network computation into a short description of the way networks may communicate, and communicate with muscles, to control and coordinate anguilliform swimming, likely to have been the original vertebrate locomotor strategy.

The main focus of the paper is the network computation. Swimming provides context to demonstrate that a linear regime can, in principle, plausibly control complex behavior. We do not apply the principles to terrestrial locomotion or flight. Navigating a physical environment requires constantly calculating and compensating for gravity, joint inertia, and unpredictable ground-reaction forces, for example. We do not claim the same principles are alone sufficient to explain later-evolving land-based locomoting, only that if we are correct about the original principles, it may provide a platform to investigate more complex movement, which may be in part explained by adapting the original principles to new, land-based problems.

**Figure 1:**
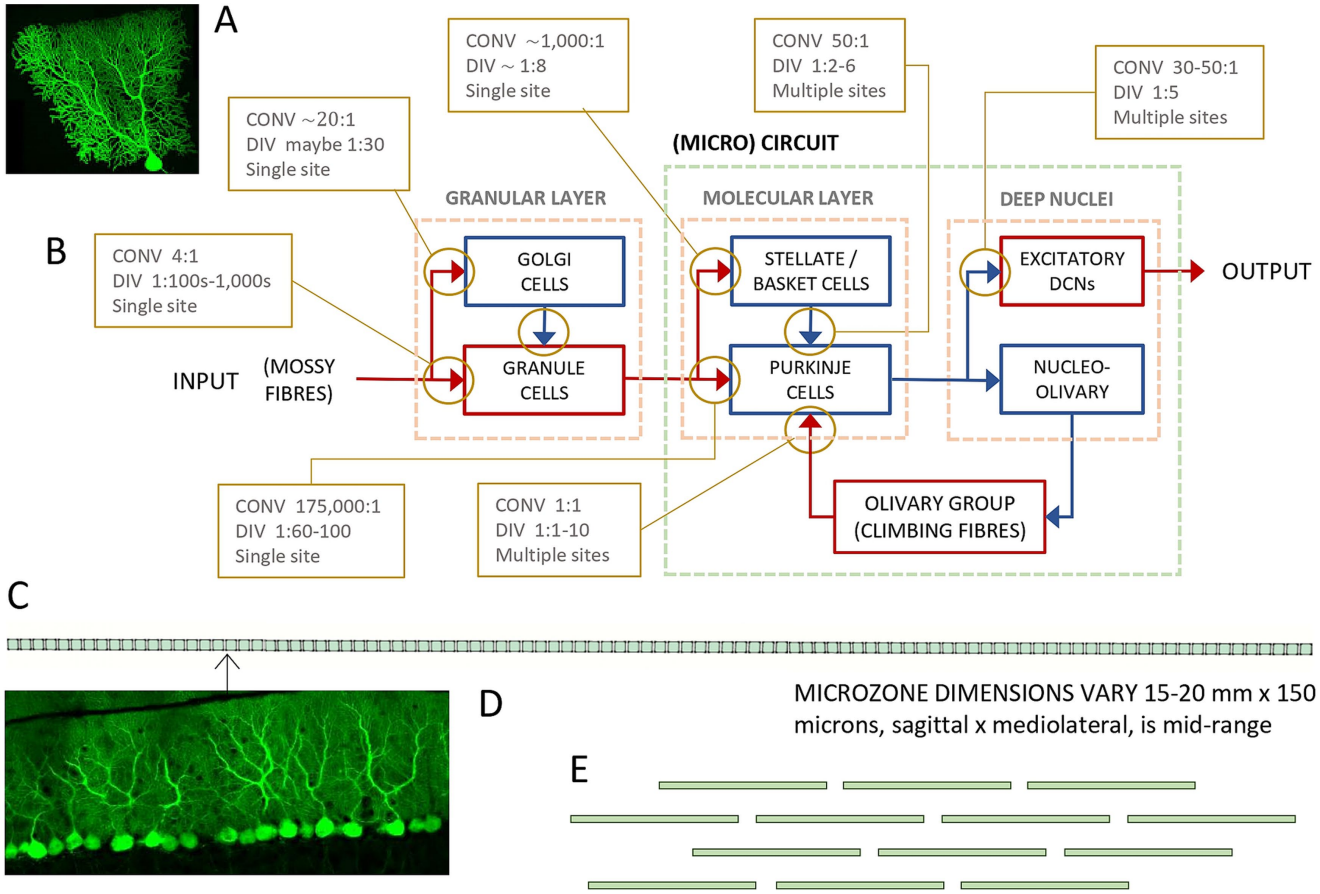
Simplified wiring diagram. **(A)** A Purkinje cell (two-photon laser scanner micrograph, courtesy Mike Hausser). **(B)** Simplified wiring diagram. Blue: GABA; red: glutamate; CONV: convergence ratio; DIV: divergence ratio; bottom line: whether an afferent cell typically makes contact at a single synaptic site or more than one. The classic cerebellar circuit is a three-‘cornered’ loop made up of a functionally defined parasagittal strip of the molecular layer termed a microzone, a group of cells in the deep cerebellar nuclei which receives the output of the microzone, and a group of cells in the (usually) contralateral inferior olive which project back to that microzone, where they terminate as climbing fibers. **(C)** Schematic to give a sense of the relative dimensions of a microzone and of flattening of Purkinje cells. Microzone dimensions probably vary, but a mid-range estimate is approximately 20,000 μm long × 150 μm wide viewed from the cerebellar surface (Garwicz et al., 1998; Oscarsson, 1979; Ozden et al., 2009; Dean et al., 2010). The line of squares represents the dendritic territories of a row of Purkinje cells from the same view as A. The Purkinje cell dendritic arbor is large from this view—300 microns vertically × 200–300 microns wide. **(D)** An actual view of Purkinje cells from this view. **(E)** Schematic of a short section of a microzone viewed from the cerebellar surface showing only the narrow silhouette of Purkinje cells. Relative dimensions of the flattened Purkinje cell arbor and the spaces between Purkinje cells are preserved. In reality, Purkinje cells are not organized into neat rows, but their large cell bodies are strictly aligned at an invariant depth, and they are all strictly oriented in the same plane, orthogonal to parallel fibers.

**Figure 2:**
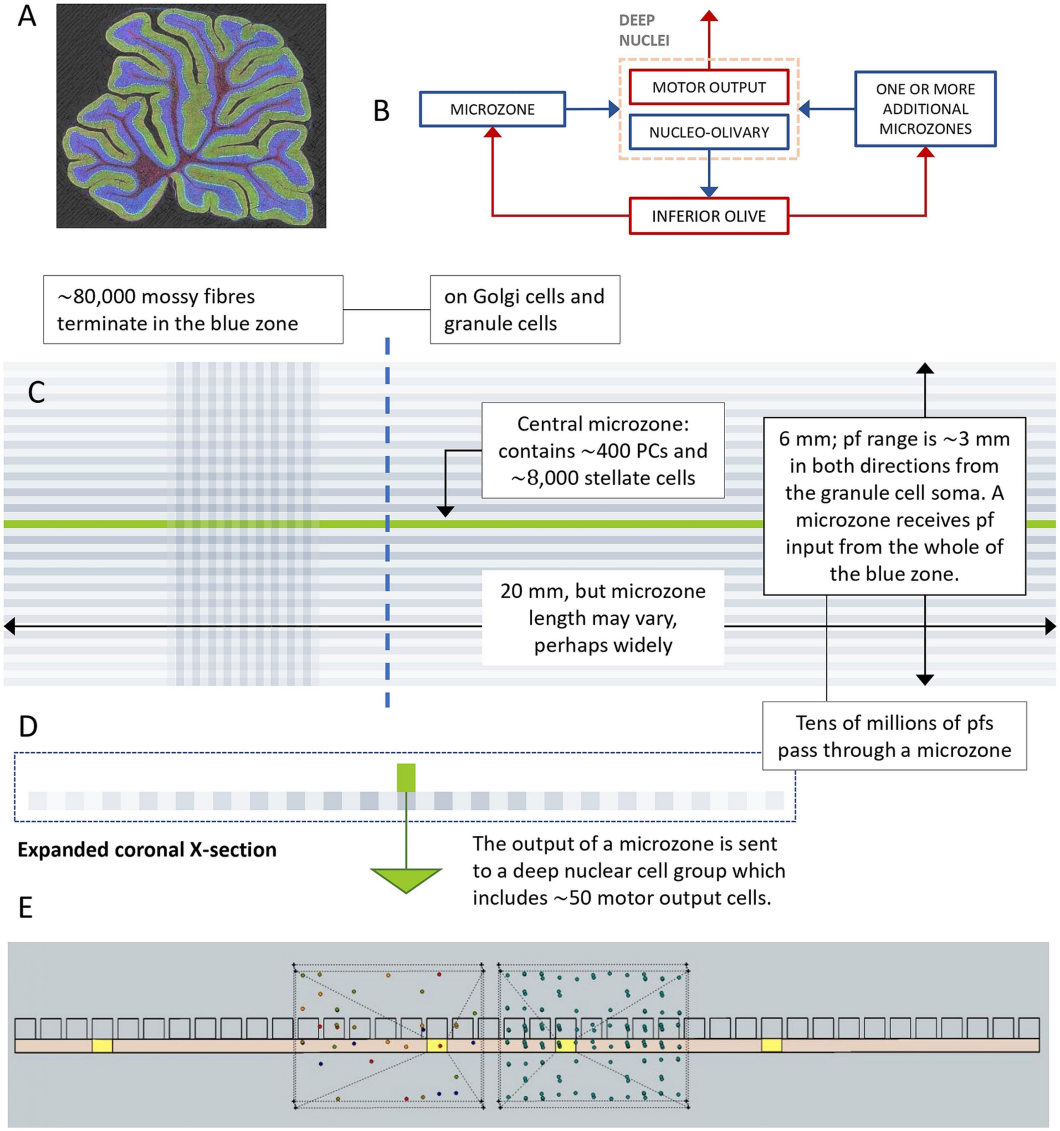
Network architecture. **(A)** Parasagittal section of the rat cerebellum (obtained by multi-photon microscopy, courtesy Tom Deerinck) showing the folded cerebellar cortex, made up of an inner layer, the granular layer (blue), and an outer layer, the molecular layer (green). **(B)** Red: glutamatergic cells; blue: GABAergic cells. There has been a strong research focus on the circuit formed of a microzone and functionally discrete cell groups in the deep cerebellar nuclei and olivary complex. Microzones may participate in functional modules made up of multiple microzones, but a single nuclear group and a single olivary group. **(C)** Schematic of a network (vs. circuit) with folding of the cerebellar cortex removed. Green strip: a microzone; blue strips: together the region that provides parallel fiber input to the middle microzone, each a ‘microstrip’; pf: parallel fiber; PC: Purkinje cell. The fade toward the top and bottom of the blue region is to represent the fact that granule cells make fewer synaptic contacts distally, so the middle microzone receives lighter contact from microstrips that are further away. The blue region is the input layer of the cerebellar network, the green strip is the middle layer, and the deep nuclear group, which receives the output of the middle microzone, is the output layer. Microstrips are functional divisions of the granular layer. The vertical stripes (‘microfiles’) are nominal divisions that we use to quantify simulations. They are about the width of a Purkinje cell. The intersection of a microstrip and a microfile has the dimensions of an average mossy fiber cluster field—a ‘field’ (so each of the squares in the chequered part of the blue region is a field). Fields are also purely notional and simply a modeling device. Approximately 4,000 fields provide parallel fiber input to a microzone (only a few are shown here, for illustration). Approximately 8,750 granule cells populate a single field, so a microzone receives approximately 350,000 parallel fibers from each microfile. **(D)** Cross-section in the mediolateral plane along the blue dashed line. **(E)** Schematic of half of the length of a microzone with folding of the cortex removed again. The row of squares represents the dendritic territories of a row of Purkinje cells, filling the molecular layer vertically. The yellow squares are field-sized locations of the underlying granular layer. The wire-framed boxes are an exploded view of two fields. The left box shows randomly scattered and intermingled terminals of 5 mossy fibers (each a different color), and the right box shows the terminals of 20 mossy fibers. Relative dimensions (of fields and terminals) are preserved to give a sense of scale. Approximately 180 mossy fibers contribute terminals to a field, and a field contains approximately 700 terminals.

Some of the proposals have been presented previously in four papers, one each on the inner (Gilbert and Rasmussen, 2025b), middle (Gilbert and Rasmussen, 2025c), and outer (Gilbert and Rasmussen, 2025a) layers of the cerebellar network, and the fourth modeling of parallel fiber synaptic learning (Gilbert, 2021). (See Figures 1, 2 for schematics of a circuit and a network, respectively.) This study pulls the ideas together into an accessible narrative and explains how the separately published parts of the theory are related. The nature of the proposals means that network computations can be simulated with high fidelity in physiological detail, working with data that is available from the literature, to generate experimentally testable predictions.

We are only concerned with the locomotor cerebellum and not with other functions or other regions of the cerebellum. We do not attempt to explain all lines of evidence because they are not all related to locomotor function. So, for example, data obtained with the conditioned blink reflex and vestibulo-ocular reflex protocols fall outside our scope.

## 2 A biologically constrained quantitative model that is not mathematically formulated

We claim that physiology implements a functionally layered network of analogue linear mathematical functions. Computations are performed by ‘nodes’. A node is the physical site of a linear function that takes as inputs signals that converge on that site. There is a node at any site where signals converge. Therefore, we know where they are, how they are connected, and how they are positioned in three-dimensional space, because the anatomical literature is rich.

Not all nodes are whole cells, although they can be—granule cells and stellate cells are nodes, for example. A node can also be part of a cell—a dendritic compartment, for example—or a specialist structure adapted for communication between cells, such as the glomerulus (in the granular layer of the cerebellar cortex) and the basket cell pinceau (in the Purkinje cell layer).

We infer the topography of inputs to the system from the terminal anatomy of mossy fibers. The inference is that mossy fiber signals, which arise from the same source, terminate in what we call a ‘microstrip’. Microstrips (in the granular layer) mirror microzonal organization (in the molecular layer). Microstrips are defined topographically by their mossy fiber input, just as microzones are defined by their climbing fiber input.

Since mossy fiber signals appear to be randomly intermingled in a microstrip, we further infer that nodes which populate a microstrip, and which are of the same type, are functionally grouped (‘code groups’). Functionally, code groups have laminar organization. Physically, some code groups occupy the same volume. In that case, nodes are still functionally organized in layers but physically intermingled.

We go on to propose that information is coded at this population level, and how code groups communicate. We claim that the same information is coded in any random sample of simultaneous outputs of a code group. The pattern of active cells and which specific cells fire at what particular rates are immaterial.

Functionally, code groups form a chain (with some recurrent pathways). The output of each code group is randomly sampled by nodes in the next. Physically, that is, of course, not possible, but anatomy simulates random sampling (with replacement). Signals, sampling, and unit computations are analogue. Analogue as it applies to computations means continuously refreshed in response to continuous inputs or to a constant turnover of inputs.

Neuroanatomy enables group-to-group communication by exploiting statistical effects of random sampling. Sampling is a passive effect of anatomy. Cell morphologies and network architecture together simulate independent random sampling by single nodes of the whole output of an afferent group. Put another way, code-grouped nodes all sample the same distribution in the same short period, effectively a moment of time, notwithstanding that single cells individually receive fixed synaptic contact from a tiny fraction of the afferent population.

Random sampling with replacement has statistical effects called the central limit theorem. In consequence, the output of a code group is reliably predicted by (and linearly related to) its inputs, as a passive result of anatomy. The central limit theorem is a repeating feature of cerebellar network data processing.

This infrastructure provides the hardware for:

- Code that occupies spatial dimensions
- Functionally defined information pathways; since code has spatial dimensions, so do pathways
- Communication at code group level, group to group
- Data processing at each step

We can replicate that virtually in biologically constrained high resolution by nominally dividing microstrips and microzones into 100 sub-regions each. These divisions are purely notional, simply a modeling device. Sub-regions of a microstrip are ‘fields’ and sub-regions of a microstrip are ‘sectors’. A network has approximately 40 microstrips, a single microzone, and a single output cell group in the deep cerebellar nuclei, so 4,000 fields, 100 sectors, and 1 output cell group.

This provides all we need to trace collectively coded data flow through a network. We ‘know’ (that is, the model makes an explicit proposal) at each step how data are processed, what the steps are, and how it is routed. By replicating a network as a computer program in node-level resolution, we are able to map the transformation of input values to output. Signals traffic is quantified at all steps.

Working this way, the reconstruction does not sacrifice computational resolution. That is, it is not a simplification or unphysiological in any way that affects how, we claim, information is coded or processed. There is no explicit overarching mathematical formulation because we claim the cerebellum does not implement one. This is not a lack of rigor but reflects our position that abstracting the computation to a formula does not describe cerebellar operations.

Parallel fiber synaptic learning is mathematically formulated. Learning, in this model, is supplemental to the main computation.

Group-to-group communication depends critically on the scaffold-like architecture of the cerebellar cortex, and therefore on the shape and orientation of cells and code groups and their arrangement in space. This is the reason we describe cell morphologies and neural architecture in detail—to understand function, we argue, it is indispensably necessary to understand the functional effect of cell shape and size, connectivity, and spatial organization.

BOX 1 **The central limit theorem**

If a population of values is randomly sampled and the mean of each sample calculated, the distribution of the sample means will be approximately normal, centered on the mean of the sampled distribution, and narrower. The original, sampled distribution can have any shape—the result is still the same. The same effects emerge if samples are summed instead of taking an average.^1^ Taking large samples gives a more nearly normal output distribution, but a large number of small samples also works.

The result of successive samplings, each step sampling the output of the one before, is therefore a progressively narrower distribution, and the mean of the distribution at each step has a linear relationship with all other steps. The first-sampled distribution can have a different (indeed, any) shape and still give this result.

We will argue that random sampling is centrally important to the cerebellar network computation. In this proposal, functionally, code groups form a chain (with some side routes). Nodes that populate a code group randomly sample values coded as the output of the afferent group (or sometimes two groups). During behavior, sampling happens simultaneously and continuously at all steps. To capture statistical effects that perform the computation, samples must be independent; that is, each sample must have no effect on the makeup of any other sample. Independence is also called sampling with replacement. Clearly, simultaneous sampling with replacement is not possible, and anyway, sampling is anatomically hardwired, so targets do not receive either an independent (or indeed a random) sample of signals, but a hardwired sample. We argue that, at each step of the locomotor network computation, anatomy simulates independence and random sampling.

## 3 Mossy fibers

Summary: A single mossy fiber gives rise to several terminal branches, each ending in a cluster of terminals. We propose: 1. All locations of a thin sagittal strip of the granular layer receive concurrently approximately the same frequency distribution of mossy fiber firing rates. 2. Single Golgi cells and granule cells each receive contact from a random sample of local terminals. 3. Therefore, they receive, individually and simultaneously, a facsimile of an independent random sample of mossy fiber firing rates received by a whole strip.

A single mossy fiber gives rise to several terminal branches, each ending in a cluster of terminals (sometimes called rosettes; average 7–8), which are strictly sagittally aligned and separated by a randomly variable but minimum distance (Wu et al., 1999; Shinoda and Sugihara, 2013; Shinoda et al., 2000; Sultan, 2001). The average size of the area that encloses a terminal cluster is 150 × 200 μm, mediolateral by sagittal, viewed from the cerebellar surface (Sultan, 2001). We term a region of the granular layer of this size a ‘field’. *Fields are purely a modeling device; they are entirely notional,* simply a way of dividing the anatomically seamless granular layer into regions of equal size. We use the notion of fields as an aid to describe physiology and to quantify the proposals. A sagittal row of 100 fields has the same dimensions as a mid-range estimate of a microzone (viewed from the cerebellar surface) (Garwicz et al., 1998; Oscarsson, 1979; Ozden et al., 2009; Dean et al., 2010). We term a sagittal row of 100 fields a ‘microstrip’. We will argue that microstrips are functionally defined by their mossy fiber input in the same way that microzones are defined by their climbing fiber input. A microstrip is a functional division of the granular layer; a microzone is a functional division of the molecular layer; a field is a nominal subdivision of a microstrip.

**Figure 3:**
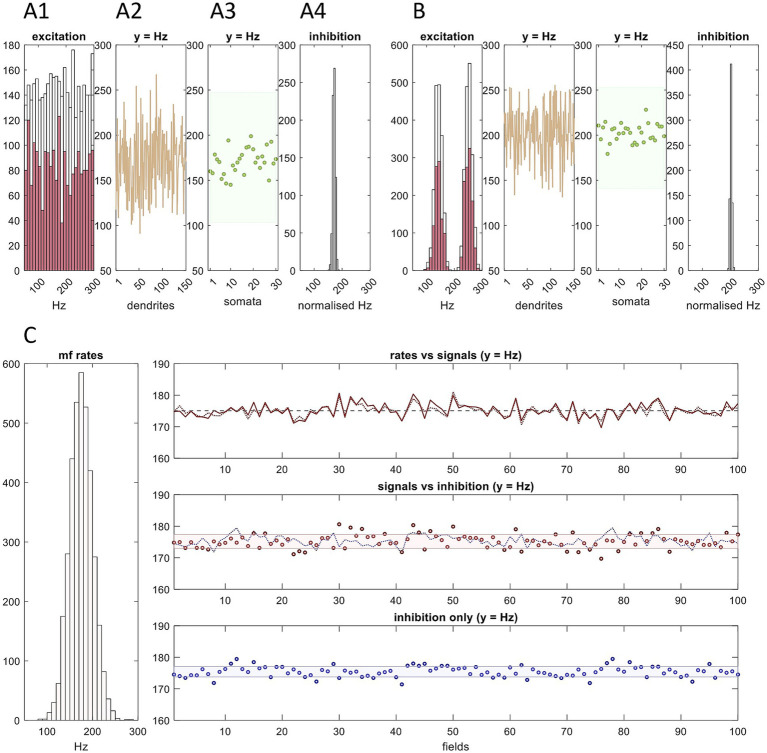
Golgi cells in a microstrip form a functional unit. This figure is adapted from Figures 2 and 3 in Gilbert and Rasmussen (2025b). **(A1)** We simulated a snapshot of mossy fiber input to a sagittal row of 100 fields at 50–300 Hz, thought to be the physiological range (Jörntell and Ekerot, 2006; citing van Kan et al., 1993). The shape of the physiological distribution of input to a microstrip is unknown and perhaps much narrower. This illustration is chosen to show that even a very wide distribution, of any shape, is converted to predictable features of the output of the Golgi cell conversion. Rate data were randomly sampled to derive input rates to each field, and copies were added to represent terminal clustering, variable cluster size, and straddling by clusters of field boundaries. Pale data: input rates to 100 fields without copies added. Dark red: input rates to a row of three fields with copies added. **(A2)** Golgi cells average 5 basal dendrites, each receiving contact from a random sample of local mossy fiber terminals, mean 4 (we estimate). The red data in panel 1 were randomly sampled 150 times to represent sampling by a 3-field population of 30 Golgi cells, 10 per field, based on a ratio of 2.5:1 with Purkinje cells. Data are the sample means, representing Golgi cell dendritic charge states. **(A3)** A2 data were randomly sampled without replacement 30 times, sample size 5, and the sample means calculated, to represent somatic integration of dendritic charge. **(A4)** A3 data were randomly sampled 700 times, sample size 8–12, reflecting convergent input from 3 fields of Golgi cells onto 700 glomeruli in the middle field. Data are the sample means, representing inhibition of middle-field granule cells mediated by rate-proportional spillover of GABA into the intraglomerular space. See Supplementary materials SM8 for the derivation of Golgi cell model parameters. **(B)** The same as A1-A4, but sampling a twin-peaked mossy fiber distribution. **(C)** Here, simulation of the Golgi cell computation is extended to a sagittal row of 100 fields, sampling the mossy fiber distribution on the left. Right top. Dashed line: the mean of mossy fiber rates received by all fields; dotted line: the mean of mossy fiber rates received by each field; solid line: the mean of mossy fiber rates received by each field with duplicate signals added to reflect terminal clustering. Adding duplicate signals has almost no effect. Right middle. Red: the same as the solid red data in the top panel; shaded area: SD of the red data; blue: the average, for each field, of the mean of Golgi cell rates received by each glomerulus (that is, we calculated the mean rate received by each glomerulus, then took the average for each field). Right bottom. Blue dots: the same as the blue data in the middle panel; shaded area: SD of the blue data. Inhibition of granule cells which populate a microstrip is statistically constrained to a tightly focused range which linearly tracks mossy fiber rates. Justification of a normalized scale is discussed in the main text, in Box 3.

Each mossy fiber afferent to a microstrip innervates a random sample of fields. Conversely, each field receives innervation by a random sample of mossy fibers. Since, therefore, each field receives, at the same time, a random sample of mossy fiber firing rates, all fields receive approximately the same mean and distribution of mossy fiber firing rates (remembering we use distribution to mean frequency distribution) (Gilbert and Rasmussen, 2025b) (Figure 3). Local (that is, field scale) duplication of mossy fiber signals by terminal clustering means that single Golgi cells and granule cells receive the equivalent of an independent random sample of local mossy fiber rates. Since all fields receive the same mossy fibre distribution, this has the same computational result as simultaneous independent random sampling by single cells of mossy fiber firing rates received by the whole microstrip.

Code groups in the granular layer have the shape and dimensions of a microstrip. Those dimensions are ‘topographically defined’ (see Glossary).

BOX 2 **Neuroanatomy provides the physical scaffold for a functional 3-D lattice**

Unlike the electrical wiring plan of, say, a car, which is tailored to its housing but is not dependent on that shape for the ignition and lights to work, the geometry of cerebellar wiring is indispensable to its function. The cerebellar cortex is divided anatomically into inner and outer layers, the granular and molecular layers, respectively. We propose that both layers are subdivided functionally into inner and outer levels (or strata—we use ‘levels’ and strata interchangeably). Strata are defined by the modality of signals at that level (hence ‘modal strata’).

In the C3 region, which is involved in forelimb movement, mossy fiber input to the outer level of the granular layer is a response to cutaneous stimulation, and inner-level inputs are a response to limb movement, which generates signals in muscle and joints: proprioception (Jörntell and Ekerot, 2006). The inner stratum is thicker.^2^ This organization is preserved in the molecular layer—outer-level granule cells prevalently give rise to parallel fibers at the outer level of the molecular layer, and inner-level granule cells give rise to parallel fibers at the inner level of the molecular layer (Zhang and Linden, 2012; Palay and Chan-Palay, 1974).

Strata lie parallel with the cerebellar surface, orthogonal to microstrips (in the granular layer) and microzones (in the molecular layer), creating a 3-dimensional functional lattice. Each microstrip is functionally subdivided into an inner level and an outer level, and each microzone is also functionally subdivided into an inner level and an outer level. The flow of information through a network is overridingly in one direction: microstrip → microzone → deep nuclear group. A single microstrip has output to approximately 40 microzones, 20 each side (based on parallel fiber range and a midrange estimate of microzone dimensions); conversely, a microzone receives parallel fiber input from 20 microstrips each side.

Within that basic architecture, there are recurrent networks, feed-forward circuits, and feedback loops^3^. Typically, these are confined to communication between cells in the same microstrip or the same microzone. In most aspects of their organization, microstrips do not communicate with other microstrips, and microzones do not communicate with other microzones. An exception is feedback received by Golgi cells via parallel fibers. This is discussed in the section on granule cells.

## 4 Golgi cells and glomeruli

Summary: The shape, range, and orientation of the Golgi cell axonal plexus together have the result that Golgi cells in a sagittal row of three fields all innervate the whole or almost the whole of the middle field. Accordingly and conversely, glomeruli in the middle field each receive convergent inhibition from a random sample of Golgi cells in a 3-field row. The computational effect of two stages of independent random sampling—first of mossy fiber firing rates by Golgi cells, and then Golgi cell firing rates by middle-field glomeruli—is that the strength of inhibition of granule cells in the middle field is very narrowly focussed (effectively, it is synchronized). Since all Golgi cells in a microstrip sample the same frequency distribution of mossy fiber firing rates, all granule cells in a microstrip receive synchronized inhibition. This is a sustained state; at any time, the strength of inhibition is linearly related to the mean of mossy fiber rates by statistical effects of random sampling.

Mossy fibers contact Golgi cell basal dendrites. Golgi cells inhibit granule cells in a structure termed a glomerulus. The Golgi cell conversion of mossy fiber signals into inhibition of granule cells is in two steps.

### 4.1 Step 1 of the Golgi cell conversion

Contact by mossy fibers on a Golgi cell is on 4–6 basal dendrites (Palay and Chan-Palay, 1974), which extend radially from the soma with little branching. The number of mossy fibers that contact a Golgi cell is unreported. We take it as 20, 4 per dendrite × 5 basal dendrites (the mean). Golgi cell dendritic signals are sustained states, we suggest: at any time, a state is a linear function of the mean of inputs. Input values are mossy fiber firing rates. (Please see Supplementary materials SM2 for a review of evidence of a linear relationship of inputs and Golgi cell dendritic signals.) Dendrites form a continuous internal space with the soma. Somatic charge states linearly reflect the mean of dendritic states. Because Golgi cells in a microstrip all randomly sample the same mossy fiber distribution, Golgi cell firing rates in a microstrip are statistically constrained to a narrowed range with an approximately normal distribution whose mean is coupled to mossy fiber rates by a linear function (Figure 3).

### 4.2 Step 2 of the Golgi cell conversion

The Golgi cell axon branches profusely, giving rise to a dense plexus of fine, beaded filaments, which can look like a cloud in images. The axonal territory is elongated in the direction of the long axis of a microstrip—mean range 650 +/− 179 μm by 180 +/− 40 μm, sagittal x mediolateral (in mice: Barmack and Yakhnitsa, 2008)—and is the depth of the granular layer, which it fills vertically. There are perhaps 10 Golgi cells per field, reasoning from estimates of the ratio of Golgi cells to Purkinje cells (Lange, 1974). Please see the mossy fiber section above, Figure 2, and the glossary for the meaning of fields.

Each mossy fiber terminal is surrounded by a semipermeable membrane, which restricts neurotransmitter diffusion, a structure termed a glomerulus. Excitation of granule cells and Golgi cells by mossy fibers, and inhibition of granule cells by Golgi cells, take place here. There are approximately 700 glomeruli per field (derived from an estimate of the number of mossy fibers which innervate a field and the average number of terminals per cluster; Sultan and Heck, 2003).

The dimensions and orientation of the Golgi cell axonal plexus mean that the axonal territory of a single cell fills approximately the volume of a sagittal row of three fields. Therefore, the Golgi cell population of a sagittal row of three fields, approximately 30 cells, all innervate the whole of the middle field, or close to it. (They have a combined range of 5 fields.) Consequently, glomeruli in the middle field each randomly sample a three-field ensemble of Golgi cells. An ensemble can be thought of as a minimum functional unit. Ensembles are a purely nominal grouping of Golgi cells, which we use to divide the anatomically seamless granular layer into chunks we can simulate. In reality, ensembles do not operate as separate units but overlap sagittally in a continuous manner to form functional strips defined by mossy fiber topography. The simulation acknowledges and reflects the anatomical overlap of ensembles.

Golgi cell axons enter glomeruli, where they inhibit granule cells (Hámori and Szentágothai, 1966). The large majority of inhibition (98%) exerted is by spillover (Duguid et al., 2012), where GABA released into the synaptic cleft spills out into the glomerular space. This is detected by high-affinity GABA_A_ receptors located perisynaptically and extrasynaptically on granule cells (Nusser et al., 1998; Brickley et al., 1996). A glomerulus receives continuous input from Golgi cells (Ruigrok et al., 2011). As a result, there is a sustained build-up of glomerular GABA during behavior (Rossi et al., 2003) at an adjustable concentration controlled by afferent Golgi cell firing rates (Mapelli et al., 2014). Signaling by spillover is sometimes assumed to be ambient and slow. However, the action of glomerular GABA spillover has a fast phasic component (Mapelli et al., 2014).

The result is that glomerular GABA concentration is given by a linear function of an independent random sample of the firing rates of Golgi cells which innervate a field. At any time, glomeruli in a field all simultaneously sample the same Golgi cell distribution. The result is that inhibition of granule cells in a field is statistically constrained to a tightly focussed distribution (effectively, it is synchronized), at a strength which is predicted by mossy fiber rates (Figure 3). The ‘computation’ at each step is an ongoing condition, a state of change.

### 4.3 Inhibition of granule cells in a microstrip

Since all Golgi cells in a microstrip sample the same mossy fiber distribution, inhibition of granule cells is effectively synchronized in a microstrip and scales with mossy fiber rates.

BOX 3 **Modeling with normalized data; analogue dendritic signals**

We argue that the cerebellar network is a layered web of linear functions. Evidence of linearity has tended to be under-represented by theory/modeling, partly because it is not obvious how to reconcile linear relationships with the known non-linearities of biophysics. Network models sometimes deal with that by using abstractions. For example, axonal signals (firing rates) are generally represented by a value. The rationale is that, if firing rates are a biological stand-in for numbers, we can represent signals as an abstraction, without needing to include underlying biophysical events as model parameters.

Rate information transitions through several biophysical steps that connect the input and output of a single neuron. Experimental recordings are made on a prescribed scale that depends on the measurement. Scales are arbitrary *per se*, but bring consistency to data collection. However, they may not be the correct measure to compare and relate *information* coded at successive steps.

The case for treating dendritic (and somatic) signals in the same way as axonal signals is less accepted, probably because it is less evident that biology has controlled for biophysical noise. Dendritic recordings, for example, lack the *appearance* of an information scale that firing rates naturally suggest. We extend the same convention—and a linear regime—to dendritic signals. It forms part of our thinking that the relationships of biophysical events measured in the usual way may mask a linear relationship on a normalised scale of the information it codes. We observe that although ‘cerebellar neurons and synapses have nonlinear intrinsic properties (Hoebeek et al., 2010; Ishikawa et al., 2015; Jahnsen, 1986; Loewenstein et al., 2005; Person and Raman, 2012a), these properties are disengaged or compensated in ways that keep the cerebellar network operating in a linear regime (Alvina et al., 2008; Rössert et al., 2014; Schonewille et al., 2006; Turecek et al., 2017; van Vreeswijk and Sompolinsky, 1996; Raymond and Medina, 2018).

Experimentally measured dendritic recordings are often made in artificially manipulated conditions in order to isolate the relationship between the controlled and observed variables, to determine if there is a correlation. The stimulation applied is typically discrete (that is, an episode of limited and usually short duration); therefore, so is the response. Partly for this reason, dendritic signals are often regarded (and modelled) as discrete events. We postulate that during behavior, dendritic signals are analogue, a sustained state that is modulated by inputs. In this proposition, sustained signals are not a simplification to avoid modeling problems but a physiological strategy to implement a linear regime.

To simulate the regime, we use a normalized scale. With this approach, values that represent firing rates are not always typical of reported values. Our interest is in relationships: how information is coded at each step and how that is related to the code at other steps. Tuning the function at each step to get desired rates would be uncomplicated, but it would have no merit. The merit of the model is to show that a close virtual likeness of physiology, in very high relevant resolution, which codes information the way we say it is coded, behaves in a way that can explain the evidence.

The exact calibration of each step is not necessary for the demonstration, and in fact, we suggest that it is not a system requirement for real networks. It is unnecessary for the conversion at each step to be exactly adjusted to get the right output at the end, because learning makes a network-level gain adjustment.

## 5 Granule cells

Summary: Granule cells in all fields of a microstrip simultaneously randomly sample the same distribution of mossy fiber rates, and at the same time, they also randomly sample the same narrow glomerular distribution which tracks the mossy fiber mean. As a result, 1. The percentage of granule cells that are concurrently active automatically self-regulates. 2. Granule cell firing rates have a near-normal distribution, which is narrower than the mossy fiber distribution and linearly tracks the mossy fiber mean. 3. Any random sample of granule cell firing rates, taken anywhere in a microstrip, has the same distribution.

### 5.1 Granule cell regulation

Each field is estimated to contain 8,750 granule cells (Gilbert and Rasmussen, 2025b). Granule cells have 3–5 short (15 μm) dendrites. Each dendrite extends into a different glomerulus where it receives input from a single mossy fiber and simultaneous inhibition via GABA spillover, as discussed. Each dendrite of a single cell receives contact from a different mossy fiber (an assumption; however, this is very likely in any given case (Gilbert and Rasmussen, 2025b)). Excitation and inhibition compete for influence in each glomerulus. A swing in the balance of influence may be amplified by acting through extrasynaptic receptors. Extrasynaptic GABA inhibits glutamate release, acting through presynaptic GABA_B_ receptors (Mitchell and Silver, 2000a), while extrasynaptic glutamate inhibits GABA release, acting through presynaptic mGluR2 receptors (Mitchell and Silver, 2000b).

Granule cells have been reported to fire in response to a single active input in experimental conditions using stimulation that excites very high mossy fiber rates (Rancz et al., 2007). However, other evidence suggests that one is not sufficient (Chadderton et al., 2004; Duguid et al., 2012; Bengtsson and Jörntell, 2009), and three may be the minimum (Billings et al., 2014; Jörntell and Ekerot, 2006).

All granule cells in a microstrip independently randomly sample the same frequency distribution of mossy fiber rates at the same time (rather, physiology mimics independent random sampling, as discussed in the mossy fiber section). As inhibition is effectively synchronized between fields, mossy fiber signals compete in all fields with the same strength of inhibition. Therefore, the outcome of the glomerular competition depends, in each glomerulus, on the strength of the mossy fiber signal. It follows that all granule cells in a microstrip have the same probability of receiving at any time a minimum of three dominant mossy fiber signals.

**Figure 4:**
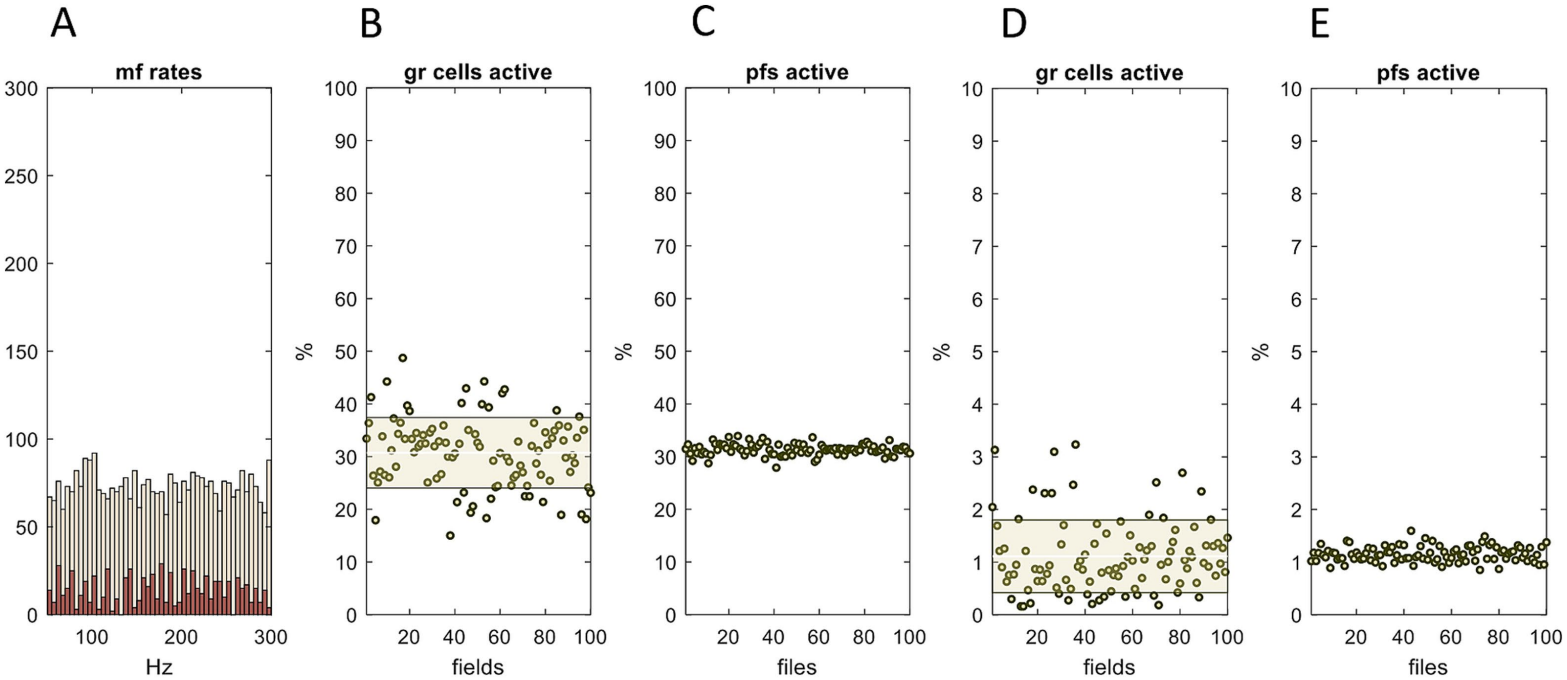
Feed-forward (mossy fiber → Golgi cell → granule cell) inhibition regulates the percentage of active granule cells and (even more strictly) parallel fibers. **(A)** Mossy fiber rates were generated in the same way as Figure 3, simulating input to 41 microstrips, making up the input layer of a network. Input to each microstrip was randomly generated with a uniform distribution (mean 175 Hz). The data shown are for a single microstrip, randomly selected, and represent a moment of time. **(B)** Input to each microstrip was passed through the granular layer simulation. The simulation returns, *inter alia*, the number of granule cells that meet the conditions to fire in each field. Data points: the percentage of granule cells that fire in each field of a single microstrip, with the Golgi cell coefficient set where approximately 30% of granule cells fire; shaded area: SD; white line: mean. **(C)** From the number of granule cells that fire in each field, we calculated the number that fire in each ‘microfile’ (abbreviated to file). A microfile is a mediolateral row of fields. Microfiles are notional divisions of the granular layer and lie at right angles to microstrips (they are the ‘vertical stripes’ in Figure 2). They are simply a modeling aid. Data are the percentage of granule cells that fire in each microfile (there are 100 microfiles because microstrips are 100 fields long) and therefore the percentage of parallel fibers that are active in the immediately overlying strip of the molecular layer. **(D,E)** The same as B,C except that the gain applied to the Golgi cell computation was adjusted by a constant so that the mean percentage of active granule cells is ~1%. The range of the *y*-axis is 0–10%, a tenth of the range in B,C. Sparsely coded parallel fiber activity is in a very narrow range. The simulation does not include local (field scale) feedback via granule cell synaptic contact on Golgi cells, which likely narrows the data range in D (and therefore also E). We reran the simulation varying the mossy fiber distribution (not shown). The results are independent of the bandwidth, shape, and range of the distribution of mossy fiber firing rates.

The number of granule cells is large even in a single field, so control of the probability that any single cell fires reliably regulates the proportion of granule cells that are active at any time (Figure 4). Moreover, since it is random which granule cells meet the conditions to fire at any time, and what mossy fiber signals they each receive, the subpopulation that fires is a random sample, and it is random which active cells fire at what rates.

The Golgi cell ensemble conversion preserves a linear relationship between the strength of inhibition and the mean of mossy fiber rates (previous section and Figure 3). Because inhibition automatically scales with mossy fiber rates, the probability is constant: it is invariant with time and location. Therefore, the percentage of granule cells that are active at any time is also constant: the system automatically self-regulates.

While feed-forward inhibition of granule cells (mossy fiber → Golgi cell → granule cell) is the primary regulator, it is not the only one. Two other mechanisms (not included in our simulation) provide important refinement, both by the feedback pathway: mossy fiber → granule cell → Golgi cell → granule cell. The evidence is not conclusive that mossy fibers have a stronger effect. We propose they do so for the functional reasons described here and also because while a Golgi cell receives contact from many more granule cells (hundreds) than from mossy fibers, only a small percentage are active at any time (perhaps <1%) if the granule cell code is sparse.

In fact, the feedback pathway has two routes. One is local, where the loop is formed by granule cell ascending axon contact on Golgi cell basal dendrites (Cesana et al., 2010), and the other is formed by parallel fiber contact on Golgi cell apical dendrites. The first provides fast local (field scale) tuning, we suggest. A Golgi cell receives ~400 functional synaptic contacts from this source (making up approximately 25% of inputs). During sustained mossy fiber discharge, synapses from local granule cells contribute equivalent charge to Golgi cells as mossy fiber synapses and ‘display similarly fast kinetics’ (Cesana et al., 2013). As a result, local inhibition of granule cells receives a bidirectional adjustment at short latency in proportion to the local number of active granule cells, with (we suggest) a self-regulatory effect. This provides important alignment of relative variance (or, put another way, uniform dispersion of active granule cells) between all locations at local scale, key to the expression of granule cell rate code.

The second mechanism, and further fine-tuning, acts through parallel fiber synaptic contact on Golgi cell apical dendrites. This is a long-standing idea (Albus, 1971). The function is to provide homeostatic regulation of the proportion of active parallel fibers by forming feedback loops that connect microstrips. Gap junctions between apical dendrites may provide the mechanism for Golgi cells to detect the density of parallel fiber activity (Gilbert and Rasmussen, 2024) and respond with rectifying modulation of granule cell inhibition, stronger if parallel fiber density is too high and weaker if it is too low.

### 5.2 The granule cell code

**Figure 5:**
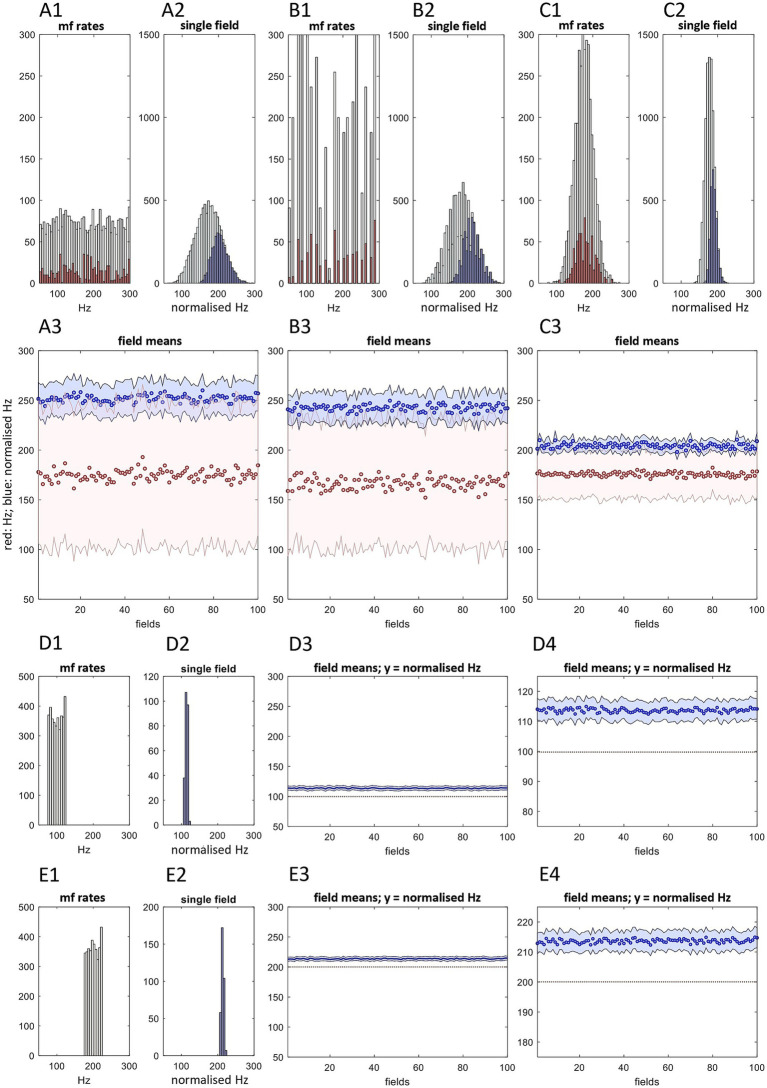
Information coded in granule cell ascending axon signals has spatial dimensions that mirror microzonal organization. This figure is adapted from Figures 5 and 6 in Gilbert and Rasmussen (2025b). The Golgi cell ensemble conversion was incorporated into a simulation of recoding in the cerebellar granular layer. **(A1)** Mossy fiber data were generated in the same way as Figure 3. Pale data: mossy fiber firing rates received by a microstrip; red data: copies-added data (to reflect terminal clustering) for a single field selected at random. **(A2)** Red data for each field were randomly sampled 8,750 times, sample size 4, representing a per field population of 8,750 granule cells, each with 4 dendrites, which each (meaning, each dendrite) receive contact from a single mossy fiber terminal. Pale data: the mean of input rates to each granule cell (the ‘sample means’) in one field; blue data: sample means of the subgroup of granule cells that meet the conditions to fire, assuming ~30% fire, for illustration. We propose that granule cell firing rates are a linear function of inputs; all data represent firing rates on a normalised scale. **(A3)** Mean and SD (shaded area) of A1 red and A2 blue data for each field of a microstrip. Blue data are statistically constrained to a narrow range: all fields return almost the same blue frequency distribution. **(B1–B3)** The same, but sampling a discontinuous mossy fiber distribution, with the same results. **(C1–C3)** The same, but sampling a normal mossy fiber distribution, SD 25 Hz. The C3 blue mean and SD are constrained to a narrowly focussed range. Summary: granule cell rates in a microstrip are statistically constrained to a narrowed range and linearly track the mean of mossy fiber rates. **(D1)** Mossy fiber rates were derived in the same way as A1 but constrained to a smaller range, with a mean of 100 Hz. **(D2)** Frequency distribution of the mean of input rates to granule cells that meet the conditions to fire in a single field selected at random, assuming ~3% fire. **(D3)** Dark and pale blue: mean and SD of granule cell rates in each field of a microstrip; dashed gray line: the mean of mossy fiber rates. There is a strict and reliable linear relationship between mossy fiber rates and granule cell rates. These features are independent of the shape and range of the mossy fiber distribution (for distributions that were tested; other distributions are not shown). **(D4)** The same data plotted with a shorter *y*-axi*s* range. **(E1–E4)** The same as D1–D4, but the mossy fiber distribution is shifted to the right, with a mean of 200 Hz. The linear relationship of mossy fiber rates and granule cell rates is preserved.

The granule cell firing rate is a linear function of the sum or mean of (a minimum of three) dominant mossy fiber signals, we moot. (This is contentious. Evidence of a linear relationship is discussed in Supplementary materials SM2.) Since granule cells independently randomly sample mossy fiber rates, we see the usual statistical effects—mossy fiber signals are converted to a narrowed range of normally distributed granule cell rates, whose mean is linearly related to the mean of mossy fiber rates, by the central limit theorem (Figure 5).

The range of granule cell firing rates is additionally narrowed because dominant mossy fiber signals must all exceed the inhibitory threshold. As a result, excitatory input rates to active granule cells are confined to the higher end of the mossy fiber range.

Since all granule cells in a microstrip sample the same mossy fiber distribution, the granule-cell-encoded output of all fields in a microstrip codes (at any moment) the same information. Moreover, the same information is coded in any random sample of granule cell signals (Figure 5).

### 5.3 The parallel fiber code

The granule cell axon rises into the molecular layer, where it divides into two to form parallel fibers. The two branches travel in opposite directions for approximately 3 mm (Brand et al., 1976; Mugnaini, 1983) parallel to the cerebellar surface and to other parallel fibers. Parallel fibers lie at right angles to microstrips and microzones. A single microstrip has output to some 40 microzones, 20 on each side. Conversely, therefore, a single microzone receives convergent parallel fibre input from some 40 microstrips, again 20 on each side. As a result of this architecture, all locations of a microzone, along its full length, simultaneously receive from each afferent microstrip, at any given time, the same frequency distribution of parallel fiber firing rates, and also receive the same (regulated and constant) number of signals.

BOX 4 **The inner-level parallel fiber code is analogue**

Inner-level granule cells receive signals from muscles that code muscle length and the rate at which it changes (referenced in the main text). Locomotor sequences of muscle contraction and extension, therefore, generate inner-level parallel fiber signals that code information about movement. Our focus is this level because, we will argue, the inner level code is the primary controller of cerebellar locomotor outputs.

A high density of mossy fibers that terminate at inner level are active—many are continuously active during behavior. Regardless of how many are active (including the case where they are all active), they are converted to a strictly regulated low density of active granule cells—and therefore parallel fibers—in a random pattern (described in the section on granule cells).

Unusually for neurons in the cerebellum, granule cells fire in short, high-frequency bursts (this is part of a strategy that conserves energy—please see the Discussion section). 10–20 ms has been reported in adult cats (Jörntell and Ekerot, 2006) and 8–40 ms in rabbits (van Beugen et al., 2013). For part of a burst, in most cases, the whole cell is active (that is, the transmission time of a spike is shorter than the sum of interspike intervals). However, the active part of a signal seems to be the initial segment. At the parallel fiber-Purkinje cell synapse, high-frequency bursts cause short-lived facilitation of release probability within the first few spikes, rapidly followed by a reduction of neurotransmitter release (van Beugen et al., 2013). This may be a noise reduction strategy, so that the length of a burst does not vary the postsynaptic response, or therefore interfere with an exclusively rate-coded response.

We propose that inner-level parallel fiber signaling is analogue at collective level. This is achieved by a combination of (i) a constant fast turnover of active parallel fibers; (ii) randomization of the pattern in which they are active; and (iii) it being also functionally random which cells fire at what rates. Bursts are variable in duration with asynchronous onset and offset times, so the pattern of active cells (and permutation of firing rates) changes incrementally every moment. There is a full turnover every 2–4 hundredths of a second (the refresh rate is about equal to the average duration of a burst). Input to all parts of a microzone is refreshed at this speed.

Purkinje cells, stellate cells, and basket cells all receive contact from a random sample of passing parallel fibers, and therefore receive, at any moment, a random sample of parallel fiber firing rates, at a constantly changing pattern of active synapses. Parallel fiber rate information is coded at collective level in the frequency distribution of granule cell firing rates, as discussed. The code is continually updated by the turnover of active granule cells. As a result, Purkinje cells, which receive hundreds of signals at any given time, even if only a very small fraction of granule cells is active, receive what is functionally an analogue signal, and therefore a smoothly modulated effect. The speed at which the parallel fiber code is updated is not the duration of a full turnover but much faster, because the code is updated continuously, each time a signal is added or removed.

## 6 Stellate cells

Summary: Inner-level stellate cells in a sagittal strip of the molecular layer, such as a microzone, each independently randomly sample the same frequency distribution of parallel fiber firing rates, simultaneously, at any given time. The stellate cell firing rate is a linear function of the (continuously recomputed) mean of inputs to the cell. As a result, stellate cells in a microzone are statistically constrained to firing rates with a strongly focussed distribution which linearly tracks the parallel fiber mean.

The morphology of molecular layer interneurons varies continuously with the depth of the cell body (Paula-Barbosa et al., 1983; Palay and Chan-Palay, 1974), but they are usually divided into stellate cells, which occupy the upper two-thirds of the molecular layer, and basket cells, found in the lower third. Possibly, they are different genotypes (Palay and Chan-Palay, 1974). All molecular layer interneurons can fire at rates of up to 300 Hz (Kim and Augustine, 2021) and receive synaptic contact in passing from parallel fibers.

Both axonal and dendritic stellate cell territories are severely flattened in the sagittal plane, orthogonal to parallel fibers. Stellate cells occupy the spaces (of about 40 μm) between Purkinje cells, which they contact synaptically and inhibit.

As any random sample of inner-level granule cell firing rates in a microstrip has the same distribution, and all locations of a microzone receive parallel fiber input from all the same microstrips, all locations of a microzone receive the same inner-level parallel fiber distribution—that is, the same code—at the same time. Therefore, all inner-level stellate cells which populate a microzone simultaneously and independently randomly sample the same frequency distribution of parallel fiber rates. It has been estimated that a stellate cell receives ‘several hundred excitatory inputs from granule cells’ (Carter and Regehr, 2002 p.1310), perhaps 1,000. Contact by a parallel fiber is at a single synapse. This has the computational significance that random sampling is not skewed by multiple selections of the same signal.

The number of active inputs to a stellate cell is statistically constrained by a probability distribution that depends on the regulated proportion of parallel fibers that are active in the general population. Given (or assuming) the regulated proportion, we can calculate the probability distribution and use that in the simulation of the stellate cell computation (Supplementary materials SM3).

As in prior steps, the stellate cell computation is a linear function of the mean of active inputs, with the same statistical effects. Like Golgi cells, stellate cell dendritic signals are analogue, a sustained state which is bidirectionally modulated by inputs, we propose. Dendritic states are continuously averaged at the soma. Unlike Golgi cells, input signals are short bursts, but the high rate of turnover of the changing pattern of active inputs causes a sustained postsynaptic effect during behavior.

The total number of active inputs to a stellate cell at any time is probably low. If the regulated proportion of active parallel fibers is 0.5%, for example, the number is in single figures (Supplementary material SM3), consistent with ‘two to eight substantial EPSPs’ (Jörntell and Ekerot, 2003 p. 9628). On the face of it, a low number of co-active inputs to a whole cell is not consistent with sustained internal states. However, gap junctions, which connect stellate cells dendritically, may mitigate unevenness of the effect. Gap junctions between molecular layer interneurons were first reported 50 years ago (Sotelo and Llinás, 1972). Inner-level stellate cells are more densely electrically connected than at outer level (Alcami and Marty, 2013), consistent with the larger size of the inner-level stellate cell dendritic territory (Palay and Chan-Palay, 1974). Gap junctions are formed of a region—a plaque—where small channels directly connect the cytoplasm of neighboring neurons, allowing ions and some molecules to pass from one cell to another. Flow is passive, so charge seeks to equalise. Charge movement at short distances is fast. Cases of gated channels have been reported, but not so far in the cerebellum.

In summary, the constant turnover, high refresh rate, and changing pattern of active inputs to a stellate cell, coupled with the averaging effect of charge sharing through dendritic gap junctions, may mitigate the locally and temporally uneven postsynaptic effect of receiving a small number of signals. Gap junctions may also contribute to align dendritic states between networked cells.

**Figure 6:**
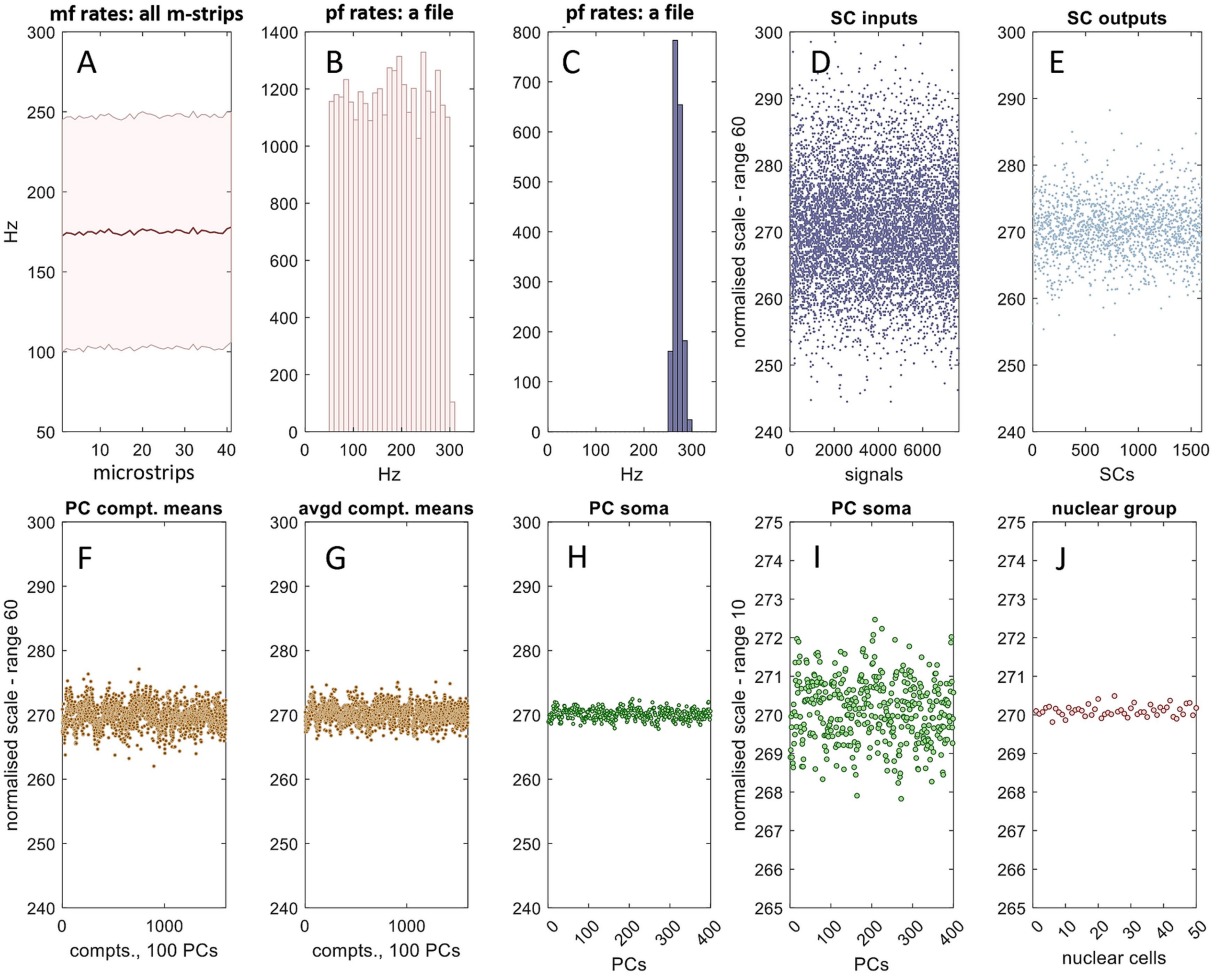
Inhibition of Purkinje cells is tightly focused and synchronized at microzone scale. This figure is adapted from Figure 4 in Gilbert and Rasmussen (2025c). mf: mossy fiber; m-strips: microstrips; pf: parallel fiber; file: microfile; SC: stellate cell; PC: Purkinje cell; compt.: PC dendritic compartment. **(A)** We simulated mossy fiber input rates to a network. Input to 41 microstrips was independently randomly generated with a uniform distribution, mean 175 Hz, SD 75 Hz, range 50–300 Hz. Rates received by each microstrip were randomly sampled 100 times to derive rates received by each field, and copies were added to reflect terminal clustering, adjusted for random variation of the number of terminals per cluster, and to reflect straddling of field boundaries. Data are the mean (red line) and SD (shaded area) of input to each microstrip. **(B)** Histogram of mossy fiber rates received by a single microfile selected at random. **(C)** The number of granule cells that fire in each field, and the rates they fire at, were derived in the same way as Figures 4, 5. The number of granule cells that fire in each microfile, and the distribution of firing rates, were derived from the field data, representing parallel fiber signals that converge on the middle microzone. Data are for a single microfile selected at random. The parallel fiber distribution is the same in all microfiles; this holds whether or not microstrips all receive the same mossy fiber rates. **(D)** The middle microzone was represented as a row of 100 sectors. Sectors are nominal divisions of the molecular layer. A sector has the same area as a field viewed from the cerebellar surface. Each sector contains 4 Purkinje cells and 80 stellate cells, assuming a Purkinje cell to stellate cell ratio of 1:16. Parallel fiber rates in each file were randomly sampled 80 times to represent active inputs to stellate cells in the middle microzone. The size of each sample was randomly generated with a distributed probability (for derivation of the probability distribution: Supplementary materials SM3). Data (approximately 7,200 values) are the population of parallel fiber rates received by 16 stellate cells in each sector (the number of inner level stellate cells in a sector afferent to a Purkinje cell, 8 each side). The rationale for using a normalized scale is discussed in Box 3. **(E)** The sample means. Each data point represents a stellate cell (so there are 1,600 values; the program simulates all stellate cells—8,000 values—but the data are thinned for presentation). **(F)** The Purkinje cell dendritic arbor was imagined as 16 compartments. Compartments were represented by randomly sampling stellate cell data to reflect synaptic contact on each compartment from a random sample of stellate cells afferent to each Purkinje cell. The size of each sample was randomly generated in the range 8–12. The sampled data represent stellate cells that immediately neighbor each Purkinje cell on one side, plus sagittally neighboring inner-level stellate cells. The model postulates that dendritic compartment membrane voltage is a linear function of inputs. Data are the sample means for a single Purkinje cell in each sector. **(G)** We repeated F but sampling stellate cells on the other side, then took an average of the two sample means for each compartment. Compartment data are tightly grouped across all sectors. **(H)** The effect on Purkinje cell firing of integrating dendritic signals was represented by taking the mean of the compartment means for each cell, giving 400 values: 4 Purkinje cells per sector x 100 sectors. **(I)** The same data, but with a shorter y-axis range to give a more detailed sense of the spread of the data. **(J)** Sample means of 50 independent random samples of the Purkinje cell soma data, sample size 30–50, representing the effect received by DCNs that receive the output of a microzone. When we ran the simulation with a normal distribution of mossy fiber rates (SD 40) and a discontinuous mossy fiber distribution (range 50–300 Hz), we obtained the same narrowly focused output (not shown).

The output of this step is inhibition of Purkinje cells at a narrow range of stellate cell firing rates (Figure 6). By this step in the network computation, firing rates are tightly grouped, so that Purkinje cells which populate a microzone receive coherent (effectively synchronized) inhibition (Gilbert and Rasmussen, 2025c). Note that it is unnecessary for stellate cells to individually ‘read’ the parallel fiber code for this result. The physiologically irregular firing pattern of stellate cells (and of Purkinje cells) is covered in the DCN section.

## 7 Purkinje cells

Summary: Like stellate cells, Purkinje cells, which populate a microzone, all randomly sample the same parallel fiber distribution at the same time. A Purkinje cell receives concurrently perhaps 200 inner-level parallel fiber signals at synapses capable of transmission (assuming 1% of parallel fibers are active in the general population), in a random pattern—enough to be representative of the parallel fiber distribution. The number is constant, but the pattern constantly changes with a fast refresh rate. Microzone-grouped Purkinje cells all receive as a result the same smoothly modulated representation of the parallel fiber code, which is continuously updated. Cerebellar cortical architecture cancels noise, so the Purkinje cell response exclusively reflects parallel fiber rate information. There is an inverted linear relationship between parallel fiber and Purkinje cell firing rates. This is the result of a rate-dependent net effect of simultaneous direct excitation and indirect (parallel fiber → molecular layer interneuron → Purkinje cell) inhibition.

### 7.1 Excitation of Purkinje cells

The Purkinje cell dendritic arbor is flattened (it is 9 μm thick; Hámori, 1992) in the plane lying at right angles to the cerebellar surface and to parallel fibers, filling the molecular layer vertically. A Purkinje cell receives excitation from parallel fibers and inhibition from stellate cells. An estimated 350,000 parallel fibers pass through the dendritic territory of a single Purkinje cell, of which around half make contact, at an average of 1.24 synapses per fiber, that is, one and sometimes two (Napper and Harvey, 1988a; Napper and Harvey, 1988b; Harvey and Napper, 1988; Harvey and Napper, 1991).

The number of parallel fibers that make contact on a Purkinje cell is much higher than for stellate cells (175,000 vs. ~1,000). Purkinje cells receive contact from one in two parallel fibers and therefore receive one in two signals (vs. around one in 80 received by a stellate cell), although the functional number is lower (nearer one in ten) because a large majority of parallel fiber–Purkinje cell synapses seem to be strongly depressed (‘silent’), perhaps 80–85% (Isope and Barbour, 2002).

As a result, Purkinje cells receive a large number of active inputs at ‘working’ (that is, not silent) synapses, which is a random sample of around one in ten parallel fiber signals. Parallel fiber contact is made exclusively on dendritic spines that arise in a spiral pattern from narrow-diameter tertiary dendrites (O'brien and Unwin, 2006). Each spine receives no more than a single synapse. Tertiary dendrites are equally and densely expressed in all parts of the Purkinje cell dendritic territory, so that parallel fibers which pass through the field have an equal chance of making contact, therefore with no sampling bias. All Purkinje cells in a microzone sample the same frequency distribution of parallel fiber firing rates, at the same time, because all locations of a microzone receive the same parallel fiber code, which is contained in any random sample of signals. Samples are independent (sampling has no effect on the makeup of other samples), and like stellate cells, severe flattening of the Purkinje cell arbor mitigates interference with the computation by repeat selections of the same signal.

Communication in this form makes it unnecessary for parallel fiber signals to be sent to or received by specific individual Purkinje cell targets. It is only necessary that all parallel fibers make contact on the way through a microzone on a number of Purkinje cells given by the same probability distribution. This is achieved by an equal probability of contact on any single Purkinje cell coupled with microzone dimensions and orientation to parallel fibers.

The pattern of active parallel fibers may not always be perfectly random (some clustering has been reported; Wilms and Häusser, 2015). We propose that clustering is not functional and does not de-randomise sampling of parallel fiber signals, for these reasons: (i) a random sample of only one in ten signals is received at a synapse that is capable of transmission, and (ii) the spiral configuration of Purkinje cell dendritic spines reduces the chances that closely neighboring parallel fibers make contact on the same cell.

Physiology combats noise. Noise is unwanted interference by variables that code nothing. The random pattern of active parallel fibers, tight regulation of the proportion and density that are active, and firing rates being randomly scattered among active cells, eliminate an effect of information-free variables (the pattern of active cells, the number that are active, and which cells fire at what rates). All have no effect on the response of the postsynaptic cell. This is not a passive accident but noise reduction. It eliminates interference with exclusive control of Purkinje cells by the parallel fiber rate code.

With this setup, Purkinje cell dendritic compartments receive a functionally analogue stream of signals, that is, a large number of active inputs in a constantly changing pattern that is fully refreshed in a time equal to the average duration of a granule cell burst (2–4 hundredths of a second). Like earlier steps, we propose that Purkinje cell dendritic signals are a sustained state, and the computation by a dendritic compartment is a linear function of the mean of co-active inputs. Therefore, we see the usual statistical effects with the result that compartment data have a further (and narrowly) focussed range. The distribution of the sample means (the mean of inputs to each compartment) is approximately normal, and the mean of the distribution scales linearly with parallel fiber rates.

### 7.2 Inhibition of Purkinje cells

Stellate cells make contact on smooth primary and secondary Purkinje cell dendrites (Palay and Chan-Palay, 1974) which extend into all parts of the Purkinje cell dendritic territory. The stellate cell axonal territory is flattened in the same plane as the rest of the cell. Contact from a single inner-level stellate cell is made through a horizontal main axon (which may extend sagittally up to 450 μm; Palay and Chan-Palay, 1974) and vertical collaterals, at multiple synapses, across multiple Purkinje cell dendritic compartments. There are estimated to be 16 stellate cells for each Purkinje cell (Llinás and Negrello, 2015). Our simulations assume that an inner-level Purkinje cell dendritic compartment is in range of a random sample of stellate cells afferent to a Purkinje cell. The statistical effects are the same as usual, so that the postsynaptic effect is a linear function of the mean of inputs to each compartment, and the distribution of the sample means is strongly focussed and approximately normal (Figure 6).

### 7.3 The net effect of excitation and inhibition

Purkinje cell firing is a linear function of the strength of direct granule cell input, when measured with inhibition blocked in slices (Walter and Khodakhah, 2006; Walter and Khodakhah, 2009). In walking mice, molecular layer interneurons are reported to reflect ‘granule cell input with linear changes in firing rate’ (Jelitai et al., 2016 p.6). The granule cell → interneuron → Purkinje cell pathway drives a fall in the firing rate of Purkinje cells as a linear function of the strength of input from granule cells, *in vitro* (Park et al., 2012). Both excitatory and inhibitory influences thus scale linearly with parallel fiber rates. Recording from Purkinje cell primary dendrites, ‘Locomotion-dependent modulation of the balance between excitation and inhibition generates depolarising or hyperpolarising dendritic *V*_m_ [dendritic membrane voltage] changes’ and these ‘linearly transform into bidirectional modulation of PC SSp [Purkinje cell simple spike] output’ in behaving mice (Jelitai et al., 2016 p.9).

What is the balance between excitation and inhibition, and what controls it, and therefore controls modulation of Purkinje cell firing rates? The competition for influence between excitation and inhibition is incompletely understood. We propose that Purkinje cell firing rates are related to parallel fiber rates by a linear inversion. In this view, the postsynaptic action of excitation is probably independent of the action of inhibition and vice versa (because a postsynaptic interaction is a less obvious strategy to accomplish a linear outcome). Both excitation and inhibition increase linearly in response to a rise in parallel fiber rates (as reported in experimental conditions, referenced above). But (we postulate) as parallel fiber rates increase, inhibition, weaker initially, becomes stronger at a faster rate. The result is net excitation of Purkinje cells by weak parallel fiber signals, but a balance that progressively and linearly favors inhibition as parallel fiber rates increase.

As far as we are aware, it has not been reported (or tested) whether there is a rate-dependent balance of influence of excitation and inhibition of Purkinje cells. There is evidence, however, that excitation of stellate cells may be weakened at low parallel fiber rates, and we cite that here as part of a possible mechanism.

Stellate cells make synaptic contact on each other (and on basket cells) at the soma, forming networks (Palay and Chan-Palay, 1974; Sultan and Bower, 1998). Stellate cells fire spontaneously at a rate that is modulated by excitatory and inhibitory inputs. They are readily excited with inhibition blocked (Carter and Regehr, 2002), but in physiological conditions, mutual inhibition causes strong attenuation of passive excitatory dendritic current flow, reducing charge transfer from synapse to soma (Häusser and Clark, 1997). The number of concurrently active excitatory inputs to a stellate cell is low and predominantly dendritic (Lemkey-Johnston and Larramendi, 1968). Inhibition reduces the excitatory time constant so that summation of the postsynaptic effect is ‘severely affected’ (Häusser and Clark, 1997, p. 675). It has also been reported that elevation of postsynaptic intracellular calcium triggered by parallel fiber synaptic activation causes an endocannabinoid-mediated, short-term suppression of neurotransmitter release (Tran-Van-Minh et al., 2016).

BOX 5 **An intrinsic drawback of rate codes**

Coding information in neuron firing rates has the drawback that it takes time to ‘read’ a signal (an intrinsic problem of coding information in a sequence). Convergence of signals (ideally onto a point) offers a solution, provided they all code the same rate. This is because the combined spike rate is higher, reducing the time needed for a readout (because the interspike interval is smaller).

In physiological practice, a ‘readout’ means the postsynaptic effect of the movement of ions across the postsynaptic membrane. For an analogue linear readout, the postsynaptic effect is continuous and at any time point proportional to the number of spikes received in a short rolling period ending at that time. The duration of the period depends partly on the time constant of the transmembrane current triggered by an afferent spike. A lot of things can affect the movement of charge across the postsynaptic membrane, and they can also affect each other. Traditionally, that has been seen as a multi-parameter problem with a computational function. We see it as the hardware of a linear relationship of pre and postsynaptic signals.

Convergence of signals that code the same rate allows the window to be short (so the readout is fast), still with a smooth effect. As far as we are aware, there is no physiological mechanism to convert convergent signals into a smooth stream of perfectly evenly spaced spikes. To approximate even spacing, the cerebellum uses the method of making afferent spike timing randomly irregular, within constraints. This can be thought of as coding information in a time-varying probability of afferent spike discharge.

Randomised spike timing renders information coded in a single signal unreadable (because a spike count in a short period is randomly related to the afferent probability). But convergence of signals that code the same probability converts afferent signals into a steady stream with very short interspike intervals or no interspike intervals at all (because spikes themselves overlap in time). This allows an intelligible readout at short latency, which is reliably proportionate to afferent spike discharge probability.

**Figure 7:**
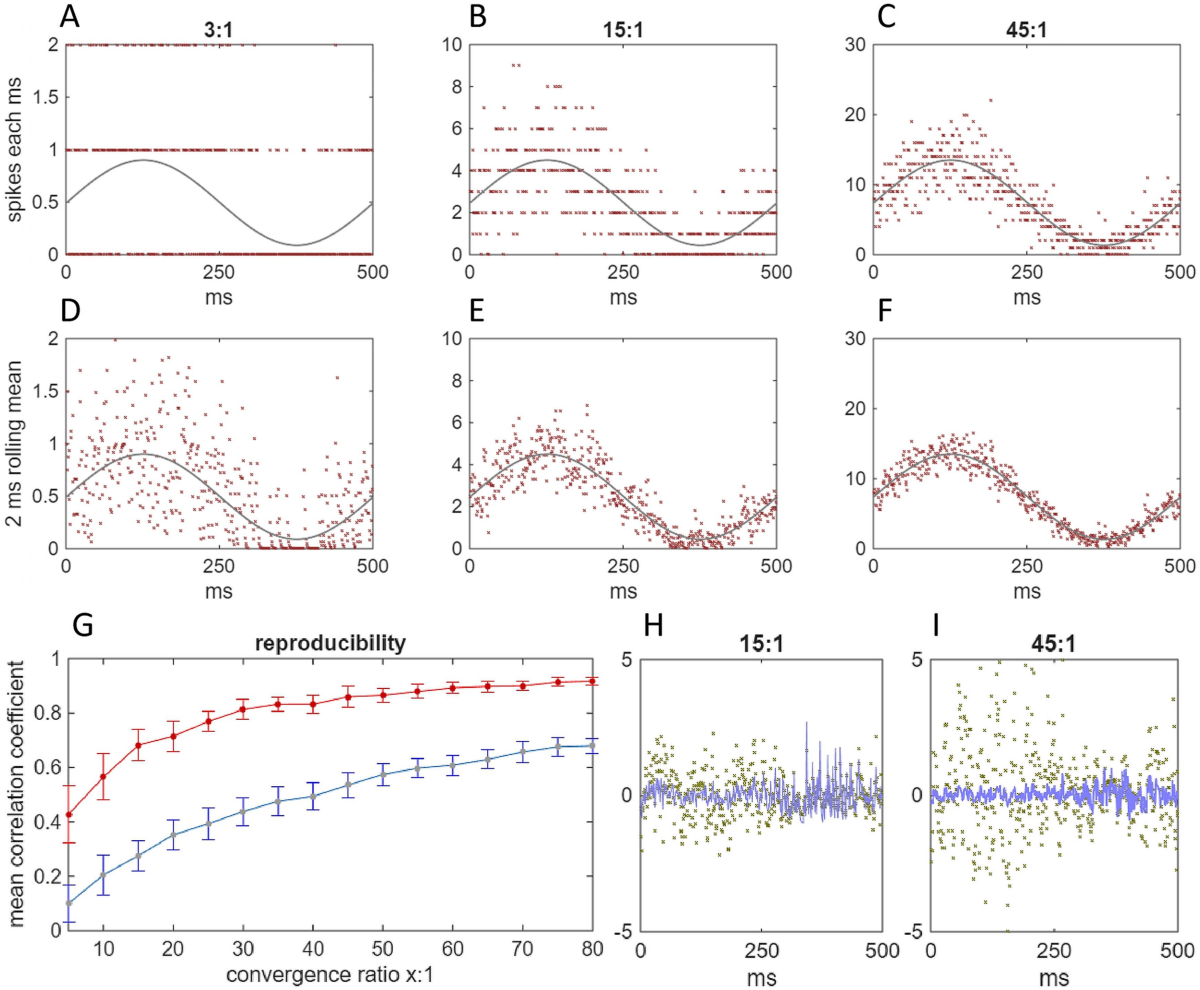
Irregular spike timing with a synchronized discharge probability generates a smoothly modulated combined input spike rate to a DCN at very high frequency. This figure is based on Figure 1 in Gilbert and Rasmussen (2025a). We propose that irregular Purkinje cell spike timing codes a time-varying probability of spike discharge. While individual signals are unreadable, convergence unmasks the code. **(A)** To simulate irregular spike timing of Purkinje cells, we generated data points with a sinusoidally time-varying discharge probability. Every millisecond, a virtual cell spikes or does not spike. Summing data sets represents the convergence of Purkinje cells onto a DCN. Red data: spike input to a DCN from 3 afferent Purkinje cells, counted in 1 ms bins. Spikes are generated with a probability that would cause Purkinje cells to fire at an ‘expected’ rate, which fluctuates sinusoidally between 30 and 300 Hz. Gray line: the ‘expected’ count/ms generated with that probability. **(B)** The same as A but simulating spike input to a DCN from 15 afferent Purkinje cells. The *y*-axis is scaled to normalize presentation for comparison. The count is in more finely graded increments relative to the expected count. **(C)** Convergent input from 45 afferent Purkinje cells, the physiological mean. The *y*-axis is again scaled for a normalized view. There is a further increase in noise reduction. A curve emerges that linearly reflects discharge probability. The combined rate of spike input to the DCN soma is very high, approximately 1,350–13,500 Hz (not taking into account the fact that each Purkinje cell terminates in 24–36 boutons). **(D–F)** At even relatively modest individual afferent rates, spikes themselves overlap in time. To reflect temporal integration, we took a 2 ms rolling average. Data converge toward a smooth curve. **(G)** We simulated 20 cycles and calculated the correlation coefficient of all possible pairs of data sets, for each convergence ratio in the range 10:1 to 80:1, with (red) and without (blue) integration. There is a strong increase in reliability in the lower end of the range, but with a diminishing effect of increasing the ratio and a low return at ratios that are higher than the physiological mean. **(H)** Green: the difference each millisecond between the spike count and the ‘expected’ count, derived from the data in E; blue: the difference divided by the expected count, a measure similar to relative variance. **(I)** The same, but derived from the data in F. Blue noise is reduced.

Purkinje cell communication with DCNs (Figure 7) uses this strategy, and non-synaptic basket cell communication with Purkinje cells (discussed in Supplementary materials SM5) exploits the same principle, we propose.

## 8 Deep cerebellar neurons

Summary: A single DCN receives convergent input from a random subset of Purkinje cells afferent to a deep nuclear group. Therefore, DCNs in a group all simultaneously receive a facsimile of an independent random sample of Purkinje cell signals. Purkinje cells have an irregular firing pattern. Spike timing is asynchronous. This is induced and functional. The combination of asynchronous Purkinje cell spike timing, convergence, and a coherent probability of Purkinje cell spike discharge generates an extremely high combined input spike rate to a DCN, which is 1. Intelligible on a very short time scale (with a therefore smoothly modulated effect). 2. Linearly related to Purkinje cell discharge probability. 3. Tightly aligned between DCNs in a group.

### 8.1 Microzone → DCN group communication

Output of a microzone is channeled down onto a smaller, discrete group of cells in the deep cerebellar nuclei, which includes perhaps 50 DCNs that carry the main output of the cerebellum. Perhaps 400 Purkinje cells converge onto a DCN group. Each Purkinje cell makes contact on an estimated 4 or 5 DCNs, and each DCN receives contact from 30–50 Purkinje cells (Person and Raman, 2012b). DCNs fire spontaneously (Mercer et al., 2016; Raman and Bean, 1999) at 70–110 Hz (*in vitro* in the mouse interpositus (Person and Raman, 2012a, Mercer et al., 2016) with synaptic inputs removed or blocked), and during behavior at a rate that is inversely related to afferent Purkinje cell rates. Modulation of inhibitory postsynaptic current in response to a changing afferent Purkinje cell rate is sensitive and fast (Telgkamp et al., 2004; Pugh and Raman, 2005).

Stellate cells form planar networks (Palay and Chan-Palay, 1974; Sultan and Bower, 1998), making and receiving synaptic contact at the soma (Lemkey-Johnston and Larramendi, 1968). Stellate cells fire irregularly *in vitro* (Häusser and Clark, 1997; Ruigrok et al., 2011; Carter and Regehr, 2002) and *in vivo* (Barmack and Yakhnitsa, 2008; Jörntell and Ekerot, 2002)—probably the result of inhibiting each other, because they fall into a regular firing pattern when inhibition is blocked (Häusser and Clark, 1997). Inhibition of Purkinje cells by stellate cells causes the regular intrinsic firing of Purkinje cells to become irregular (Häusser and Clark, 1997; Jelitai et al., 2016), and selective silencing of molecular layer interneurons causes Purkinje cell firing to fall back into a more regular pattern, resulting in motor deficits (Jelitai et al., 2016; Wulff et al., 2009).

On this evidence, irregular firing of Purkinje cells is *induced*. What function does that have? Purkinje cells generate field potentials in the Purkinje cell layer, which promote spike synchrony between neighbors (Han et al., 2018). As a result, left to themselves, neighboring Purkinje cells fall into a regular firing pattern with synchronized spike timing.

This would be a problem for clear communication between Purkinje cells and DCNs if, as we propose, Purkinje cell information is coded only in firing rates and not in spike timing. Purkinje cell-mediated inhibitory postsynaptic currents (IPSCs) recorded in DCNs decay quickly with a time constant of ~2.5 ms (Mercer et al., 2016; Person and Raman, 2012a). Even brief gaps between IPSCs make inhibition-reduced windows in which DCN spiking is more likely (Wu and Raman, 2017). This is seen, for example, in the first couple of rounds of spikes after stimulation of the mouse whisker pad, when spikes are briefly synchronized (Brown and Raman, 2018). In spike synchrony models, spike timing codes information (Person and Raman, 2012b; Gauck and Jaeger, 2000). If, instead, information is coded exclusively in firing rates, an influence of spike timing on firing by DCNs would be noise.

To prevent that, stellate cells randomise Purkinje cell spike timing, but not completely. If spikes are counted in step-locked bins in walking rats across a few tens of step cycles, a smooth (in some cases, sinusoidal) curve emerges (Sauerbrei et al., 2015). We propose that this would also be the result of recording simultaneously from (any) 30–50 Purkinje cells in the same microzone in the same cycle (Gilbert and Rasmussen, 2025a).

Rate information coded in the irregularly timed spike discharge of a single Purkinje cell is unreadable. The code emerges, however, in the combined input spike rate to a DCN. One way to think about this is that rate data are coded in the moment-to-moment time-varying probability of simple spike discharge. The probability is synchronized between microzone-grouped Purkinje cells. Purkinje cells converge directly onto the DCN soma (Person and Raman, 2012b). The compound effect of convergence, asynchrony of afferent spike timing, and coherence of discharge probability is that spikes are received by a DCN in a continuous and smooth stream at a very high combined rate, which is linearly related to the probability. As a result, the ‘hidden’ Purkinje cell rate (coded as the probability of spike discharge) is both readable by a single DCN in a very short rolling time window, and noise-reduced (Figure 7).

### 8.2 Convergence onto a ‘point’

Direct somatic contact (Uusisaari and De Schutter, 2011; de Zeeuw and Berrebi, 1996) via multiple boutons per Purkinje cell (24–36 in mice) (Person and Raman, 2012b; Telgkamp et al., 2004) means that each spike received from each Purkinje cell afferent to a single DCN is received simultaneously at multiple sites of the soma surface. The surface of the DCN soma is engulfed in boutons (~1,200), and contact made by a single Purkinje cell is dispersed and randomly intermingled with boutons of other afferent cells. As a result, the combined input spike rate is approximately the same at all locations of the somatic surface (Gilbert and Rasmussen, 2025a, Figure 3). In theoretical terms, this approaches the effect of convergence onto a point, a noise-canceling strategy, because it mitigates the randomly variable effect of spatial integration.

### 8.3 DCN group firing rates are synchronized

There is no reported internal organization of the pathway that connects a microzone and a DCN group (Apps et al., 2018 p. 663) suggesting—and in any event we propose—a single DCN receives an independent random sample of 30–50 Purkinje cell signals, and therefore the whole group receives the same combined input spike rate, and therefore the same effect, with the same timing (Figure 7). That effect, we suggest, is a continuously refreshed linear function of the mean of inputs, with the same statistical effects as previous steps. So, motor output of a DCN group is at strongly coherent (effectively synchronized) rates which are inversely linearly related to the time-varying probability of Purkinje cell spike discharge. Discharge probability is synchronized between Purkinje cells which populate the microzone afferent to a DCN group.

Synchronization of DCN rates may be enhanced by DCN axon collaterals that terminate back in the same DCN group with an interneuronal effect (Kebschull et al., 2020). As far as we are aware, convergence and divergence ratios for contact by collaterals are unknown. In theory, however, recurrent input by a DCN group to itself, received dendritically, would allow a group to resample the frequency distribution of its own output, on a loop. This may provide tonic excitatory tone, which tightens the synchrony of DCN firing between functionally grouped cells (Gilbert and Rasmussen, 2025a).

Adaptations for high-fidelity linear transmission of Purkinje cell rate information into DCN firing rates are discussed in Supplementary materials SM4.

## 9 Basket cells

Basket cells are discussed in Supplementary materials SM5. Stated briefly, basket cells communicate with Purkinje cells through a pinceau. Communication is ephaptic (Blot and Barbour, 2014). Basket cell signals that converge on a pinceau generate a local electric field around the Purkinje cell soma and first axonal segment, where action potentials are generated (Palmer et al., 2010; Foust et al., 2010). Basket cells are networked with inner-level stellate cells and have the same irregular firing pattern. We propose:

1. The compound effect of (i) convergence, (ii) asynchronous basket cell spike timing, and (iii) coherent microzone-grouped basket cell discharge probability, is coherent microzone-grouped Purkinje cell discharge probability. It is coherent because—like stellate cells and Purkinje cells—basket cells all randomly sample the same (inner level) parallel fiber distribution.
2. The pinceau-generated electric field ‘cloaks’ the Purkinje cell soma and first axonal segment, preventing neighboring Purkinje cells from falling into a regular firing pattern with synchronized spike timing (seen when left to themselves; Han et al., 2018). Synchronized spike timing would be noise, as discussed.

## 10 Parallel fiber synaptic learning

Summary: Learned changes at the parallel fiber–Purkinje cell synapse provide a bespoke gain adjustment applied to the network computation. Learning and memory are at microzone scale. Synaptic weights are functionally binary. Memory is stored as the ratio of working to silent parallel fiber synapses. All Purkinje cells in a microzone learn the same ratio. Climbing fiber instruction signals are functionally invariant. At inner level (the proprioception stratum), neither the timing of instruction signals nor the pattern of signals paired with an instruction signal is functional; that is, neither makes a difference to the learning outcome. Moreover, the learning outcome is the same whether patterns are repeated or not. The function of learning and the learning outcome are different at different depths of the molecular layer.

### 10.1 Inner level parallel fiber–Purkinje cell memory

Repeatedly pairing parallel fiber synaptic activation with a climbing fiber signal teaches parallel fiber-Purkinje cell long-term depression acting through presynaptic (Qiu and Knopfel, 2009) and postsynaptic (Hansel et al., 2001; Ito and Kano, 1982) mechanisms. Repeated unpaired parallel fiber activations incrementally strengthen transmission [presynaptic (Salin et al., 1996; Chen and Regehr, 1997); postsynaptic (Lev-Ram et al., 2002; Coesmans et al., 2004)], so that changes are reversible in both directions. The parallel fiber-molecular layer interneuron synapse is less studied, but acquired changes are reported in the same conditions, but in the opposite direction [*in vitro* (Smith and Otis, 2005; Soler-Llavina and Sabatini, 2006; Rancillac and Crépel, 2004); *in vivo* (Jörntell and Ekerot, 2002; Jörntell and Ekerot, 2003; Ekerot and Jörntell, 2003)].

In supervised learning models, which have been a strong influence on cerebellar theory, synaptic weights are individually adjusted for a pattern-sensitive compound response (for an excellent review, refer to Raymond and Medina (2018)). In our model, the learning outcome—memory—is stored as the ratio of working (robustly transmitting) to silent (severely long-term depressed) parallel fiber–Purkinje cell synapses (the ‘synaptic ratio’). Because the proportion of active parallel fibers is regulated and constant, the synaptic ratio controls the number of signals concurrently received at working synapses. Since data are coded in the statistics of collective activity, learned adjustment of the synaptic ratio linearly modulates the Purkinje cell response without otherwise interfering with the postsynaptic effect of the rate code.

We previously published a mathematical model of synaptic learning and collective memory with these features (Gilbert, 2021). The function envisaged in that paper was binary pattern discrimination, not a gain adjustment. We now regard that function as incorrect, but the mechanism as still sound.

The reason that synaptic weights polarise is in part that learning is bidirectional and indefinitely reversible. The direction depends on whether synaptic activation is paired with a climbing fiber signal. The net direction at any single synapse over many trials depends on the ratio (and sequence) of paired to unpaired activations.

The ratio of activations is critically affected by nucleo-olivary inhibitory feedback (‘feedback’). An estimated 30–35% (Baumel et al., 2009) of deep nuclear projection cells, and perhaps as many as half (Nelson and Mugnaini, 1989), form a GABAergic pathway which terminates in the contralateral inferior olive, on climbing fibers which form part of the same circuit. Cells that form the nucleo-olivary pathway fire spontaneously at a rate that is bidirectionally adjustable under the control of Purkinje cells (de Zeeuw and Berrebi, 1996; Teune et al., 1998) and therefore receives modulation by learning (that is, by the compound effect of parallel fiber synaptic changes as these are acquired).

Inhibition is received by the climbing fiber group, which projects back to the population of Purkinje cells, which in turn control feedback, forming a closed circuit. Learned parallel fiber synaptic depression weakens Purkinje cell inhibition of the nucleo-olivary pathway and, in this way, modulates feedback. In due course, as training proceeds, feedback becomes strong enough on some occasions to block climbing fiber signals, so that on these occasions the direction of parallel fiber synaptic change is reversed. At this point, there is a dynamic equilibrium between the effect of instruction on the direction of further synaptic change and the effect of change on further instruction.

This has the result of reversing the net direction of synaptic change at some synapses in response to further training. Weights migrate to the limits of their range, where they are more stable because per-activation synaptic change is in smaller steps. The result is to polarise parallel synaptic weights in proportions that depend on the relative strength of parallel fiber signals that converge onto a microzone, and of externally originating signals received by the inferior olive that normally elicit teaching signals.

In this model, evoked climbing fiber signals are functionally invariant, and lessons (evoked climbing fiber signals) are received by the whole Purkinje cell population of a microzone. All Purkinje cells in a microzone learn the same synaptic ratio. The basis in evidence for functionally invariant teaching signals, and that microzones learn as a unit, is discussed in Supplementary materials SM6, and most of it previously appeared here (Gilbert, 2021). The case in evidence for an iterative synaptic learning function, which incorporates the effect of learned changes on further learning, is made in the same paper.

A key feature of learning with these elements is that the learning outcome is independent of the timing of instruction signals and whether or not patterns are repeated. This is counter to the idea that climbing fiber signals teach an association. Pattern independence is key for the practical reason that, in physiological practice, unlike modeling, it is unlikely that patterns are reliably repeated and, arguably, recoding on entry to the cerebellum is designed in part to ensure that they aren’t. We take up this point again in the Discussion.

Important note: of course, this does not mean that climbing fiber instruction signals do not, in some cases, teach an association (eyeblink conditioning studies) or signal an error (VOR). Indeed, the function of instruction can depend on depth: in the outer modal stratum of the molecular layer, it teaches recognition of a cutaneous receptive field (Jörntell and Ekerot, 2002; Jörntell and Ekerot, 2003; Ekerot and Jörntell, 2003). However, whether or not that is the case elsewhere, we take the position that timing is immaterial at inner level in locomotor regions; immaterial means any timing teaches the same outcome.

Final note: if this is correct, it would suggest that the external drive to an olivary group is from more than one source, a cutaneous source and a second, internal source. Signals triggered by cutaneous stimulation are discrete. Internally sourced signals are analogue at time-variable strength. Signal strength co-varies in step with the strength of granule cell signals that converge onto the same microzone as climbing fibres that receive the same drive.

### 10.2 Inner level parallel fiber-interneuron memory

Inner-level parallel fiber-interneuron memory is discussed in Supplementary materials SM7. In short, the distributed probability of the number of active inputs to working synapses is almost unchanged by training, because the synaptic ratio is reversed. Inner-level synapses acquire learned changes, as they do at outer level, but the changes do not alter the response of the postsynaptic cell. Bad inner-level memory is functional: it means that the outcome of learning at the inner level is implemented exclusively by the parallel fiber-Purkinje cell synaptic ratio.

### 10.3 Outer level parallel fiber-interneuron memory

Inner and outer level parallel fiber synaptic memory have different functions which reflect the source and modality of parallel fiber signals, we propose. Outer level memory may be involved in the withdrawal reflex (not a new idea; Garwicz et al., 2002; Garwicz, 2002).

Cutaneous stimulation evokes both mossy fiber and climbing fiber signals, which converge onto the same strip of the cerebellar cortex (for example, Jörntell and Ekerot, 2003; Jörntell and Ekerot, 2011). Mossy fiber signals are received in the granular layer, and climbing fibers terminate in the overlying molecular layer. The relative timing is guaranteed because they are both evoked by the same stimulus. This teaches stellate cells at outer level to respond selectively to stimulation of an associated ‘receptive field’ (a discrete site of the body surface). Learning at this level may coordinate the withdrawal reflex in response to averse or unexpected stimulation (Garwicz et al., 2002, Garwicz, 2002). Withdrawal ends the stimulus: training teaches a discrete behavioral response to a discrete stimulus. This is consistent with the proposal that the inhibitory projection from the cerebellar deep nuclei to the inferior olive blocks the climbing fiber response to expected stimulation [Abed Nashef, University of Colorado, unpublished].

So, parallel fiber synaptic learning at inner and outer levels is involved in different behaviors with different functions. The learned response at inner level is a gain adjustment and analogue, and at outer level it is involved in the withdrawal reflex and discrete.

## 11 Swimming

Summary: The network computation calculates timely and coordinated motor outputs that power anguilliform swimming. Modular organization of the cerebellum extends outside to include spinal and peripheral nerves, forming proprioception-motor arcs. An arc is made up of multiple input pathways, a network which forms the junction of input and output pathways, and a ‘single’ output pathway (meaning not a single cell, but a few cells firing at aligned rates). Signals generated in muscles, sent to the cerebellum, are passed through the network computation to generate motor output to the other side of the body. With this source, input signals are automatically phase and wavelength sensitive. Signals in both directions, ascending and descending, are analogue, using a rate code. Communication is at multicellular level. Anatomical overlap of networks, so that neighboring microzones share most of the same parallel fiber input, automatically coordinates the relative strength and timing of motor outputs. It also contributes to the propagation of muscle contractions.

### 11.1 Default control of Purkinje cells is by inner-level information about movement

The size of the area that encloses the stellate cell dendritic territory varies with the depth of the cell body in the molecular layer, between around 80 μm^2^ superficially (that is, at outer level) and 120 μm^2^ for deeper lying cells (what we term inner level) (Palay and Chan-Palay, 1974). Stellate cell morphology varies continuously with depth, but there are clear outer-level and inner-level features (Paula-Barbosa et al., 1983; Sultan and Bower, 1998; Palay and Chan-Palay, 1974). The outer-level main axon is shorter, meandering, and may not leave the dendritic field. The inner-level main axon follows a distinct horizontal course, has a substantially longer range, and gives rise to vertically ascending and descending collaterals, so that total axonal length (and the number of synapses made on Purkinje cells if it is proportional) is greater than at outer level.

The broad division of stellate cells into outer-level and inner-level morphological types corresponds to modal stratification. This is functional, we suggest. It has the result that there is a concentration gradient of stellate cell synaptic contact on Purkinje cells, with the concentration being higher at inner level, where stellate cells receive contact from inner-level parallel fibers, which code proprioception. Stellate cells at that level make more synaptic contact, at a higher density, nearer the Purkinje cell soma, than outer-level stellate cells.

There are other depth-dependent differences. GABA spillover from stellate cell synapses enhances parallel fiber synaptic transmission to stellate cells (Stell et al., 2007; Dellal et al., 2012). Parallel fibers express functional GABA_A_Rs. Activation of these depolarizes the axon and thus increases release probability at parallel fiber synapses (Pugh and Jahr, 2013 p.16924). The relationship is reciprocal, creating a positive feedback loop. ‘GABA release from molecular layer interneurons activates parallel fiber GABA_A_ receptors, and this, in turn, increases release probability at synapses between parallel fibers and molecular layer interneurons’ (Pugh and Jahr, 2011 p.565). Possibly, and we propose, the stellate cell–Purkinje cell synaptic concentration gradient is reflected in a corresponding concentration gradient of GABA spillover, which enhances parallel fiber synaptic transmission to stellate cells at inner level (vs. a weak or no effect at outer level).

Basket cell dendrites extend toward the cerebellar surface across the inner two-thirds of the molecular layer, and, therefore, receive the same parallel fiber code as inner-level stellate cells. Basket cells, therefore, add to the influence on Purkinje cells of inner-level parallel fiber signals.

We propose that control of Purkinje cells is by the analogue inner-level parallel fiber code, the proprioception stratum. This is the default condition, which may be overridden and interrupted by the withdrawal reflex. The significance of inner-level control is that—in our model—inner-level information, which codes cycle phase and wavelength, is a plausible controller of locomotor output.

### 11.2 Modular mapping

The hyper-low resolution of granule cell information coded by a microstrip becomes even lower in the molecular layer, where parallel fiber signals are randomly intermingled (within modal strata) with the output of other microstrips. How does a microzone ‘read’ parallel fiber data without being able to discriminate between signals from different microstrips, and—in our model—without external ‘supervision’ traditionally provided by climbing fiber signals?

Swimming provides a functional context. The early vertebrate brain evolved in a marine environment, probably to power anguilliform (eel-like) swimming. The principal vertebrate brain structures are well conserved across species (Larsell, 1967; Larsell, 1970). If the basic wiring plan has been substantially conserved across evolutionary time, it may mean that the same functional principles have been adapted to later body plans and may still contribute to function today.

Cerebellospinal neurons provide ‘a direct link between the cerebellum and spinal substrates for motor coordination’ (Sathyamurthy et al., 2020). The output pathway is reciprocated by spinocerebellar neurons. Neurons of the spinocerebellar tracts are excited monosynaptically by group Ia and group Ib afferents that originate in muscle spindles and tendon organs, respectively (Sengul and Watson, 2015). Signals are transmitted by large-diameter, fast spinal neurons (Loeb and Mileusnic, 2015). Information is coded in time-varying firing rates, which reflect muscle length and rate of extension (and also contraction, but more weakly).

Fish musculature is almost entirely made up of bilaterally paired trunk muscle segments. Anguilliform swimming is powered by a repeating sequence of muscle contractions that pass backwards down the body. Axial muscle segments are called myomeres.

In our proto-vertebrate proposal, each myomere maps to a corresponding microstrip, which lies directly under the microzone that controls motor output to its antagonist. A row of myomeres maps to a corresponding row of microstrips. The cerebellum forms the junction of input and output pathways. The junction is a cerebellar network, so that the network is the apex of a proprioception-motor arc. A single arc has multiple input pathways and a single motor output pathway. Input pathways are connected to the output pathway via parallel fibers.

Input pathways originate contralaterally. Contralateral muscle extension generates signals that are sent to the cerebellum. The network computation converts inputs into ipsilateral output, which is sent to a single spinal motor neuron pool. (A muscle receives control from a single motor neuron pool, and a motor neuron pool controls a single muscle; this is still the case today.) The motor neuron pool that receives the output of a network controls the myomere opposite the middle of the myomere row, which generates input to the arc. Mapping is bilaterally mirrored.

### 11.3 Propagation of motor sequences

An executively controlled lateral head turn initiates a backward traveling sequence of myomere contractions ipsilateral to the direction of the turn. Propagation is by parallel fibers. A head turn causes extension of muscle fibers on the outside of the bend, generating signals coded in firing rates which are proportionate to muscle fiber length and the rate of muscle fiber extension. These are received contralaterally (that is, the side to which the head is turned) and in turn generate motor output to further rearward muscles, up to the range permitted by the length of parallel fibers. The strength of influence of an input pathway on outputs is inversely related to the distance between the microstrip that receives the input and the microzone that receives the influence. Both input and output signals are analogue. Generated in this way, inputs are automatically phase and wavelength sensitive.

The response of rearward muscles in turn generates inputs that drive motor output to still further rearward muscles, and so on, setting up a spiral of inputs and outputs. More strictly speaking, it is a spiral of *modulation* of inputs and outputs, because signals in both directions are analogue. Alternating left and right head turns drive left and right-sided, backward-traveling sequences of muscle contractions that are 180 degrees out of phase. Once initiated, movement (and cross-propagation between left and right sides) is aided by passive undulation of the laterally flexible trunk. We envisage that motor control by proprioception works in tandem with executive control acting through spinal central pattern generators, which sets the pace, and with spinal reflexes.

### 11.4 Coordination of related motor outputs

Coordination of outputs to near neighboring myomeres is automated by the simple mechanism that their microzone controllers share parallel fiber input, and therefore also, in proportion, the same signals. Immediately neighboring microzones receive convergent innervation by > 95% of the same parallel fibers. The amount shared by two microzones is inversely proportional to the distance between them (because more distant microzones receive input from a smaller number of the same microstrips). It is therefore also inversely proportional to the distance between myomeres that receive their output.

**Figure 8:**
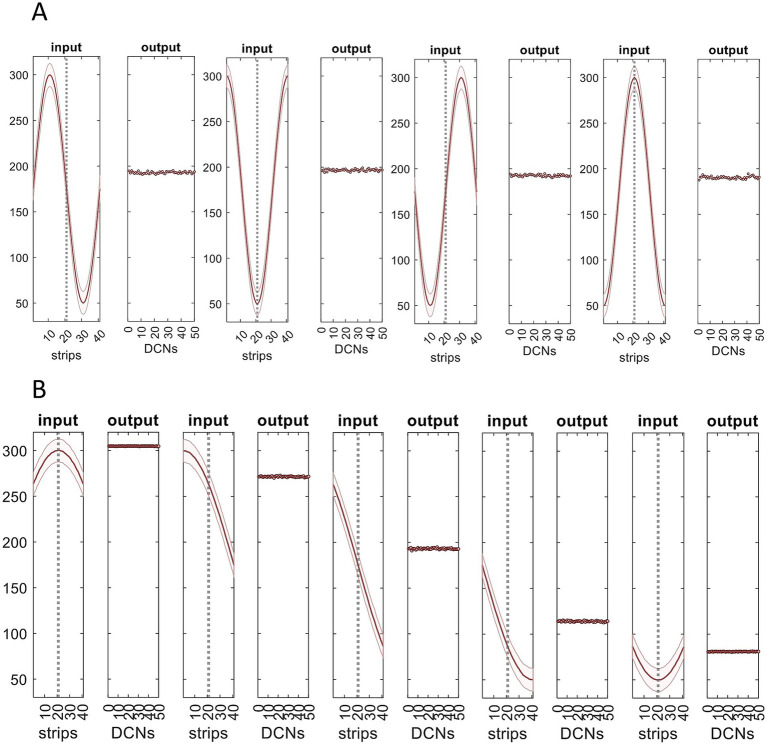
How do microzones ‘read’ parallel fiber input from multiple microstrips? This figure is adapted from Figure 6 in Gilbert and Rasmussen (2025c). A key problem for theory is how data are coded across at least tens of thousands of signals, which are received simultaneously by a microzone as input from granule cells, which are up to 3 mm away on both sides. We propose that granule cells are functionally organized into long, thin sagittal strips (which we have termed microstrips) mirroring microzonal organization in the molecular layer above. Most of the musculature of fish is made up of bilaterally paired trunk muscle segments called myomeres. In anguilliform swimming, a wave of muscle contractions passes backwards down the body. Each myomere passes through a cycle of contraction and extension. Bilaterally paired myomeres are 180 degrees out of phase. Signals are sent to the cerebellum coded as firing rates, which are proportional to muscle fiber length and rate of fiber extension. The spinal pathway is topographically organized such that each muscle segment maps to a corresponding microstrip. During swimming, therefore, each microstrip receives signals from a muscle segment at a sinusoidally fluctuating firing rate. A row of muscle segments maps to a corresponding row of microstrips. As a result, microstrips that provide parallel fiber input to a microzone (approximately 20 each side) receive as a unit, at any given instant, a sine wave-like pattern of input signal frequencies, or part of a sine wave. This is shown in panels titled ‘input’; red: mean; pink: SD; ‘strips’: microstrips; dotted gray line: position of the microzone. A network is the junction of 41 input pathways (each terminating in a microstrip) and a single output pathway (populated by 50 DCNs). Input is passed through the network computation to generate the data in the panels titled ‘output’, representing firing rates of the output cells of the network on a normalised scale. In these panels, the data are the normalised firing rates of a group of DCNs (hence 50 data points). Output is motor output. **(A)** The network computation converts input into strongly focussed firing rates of a DCN group. DCN firing rates are synchronized regardless of cycle phase but do not code the phase or wavelength of the input cycle. **(B)** If input to a network is not from a full wavelength of the trunk, however, but a smaller section, output has almost the same range as input rates and precisely reflects phase and wavelength.

We illustrate input to a network in Figure 8, in the panels titled input. These show the mean and SD of a snapshot of mossy fiber input rates to a row of 41 microstrips. Ascending signal frequencies generated by a single myomere vary with both phase (because muscles extend and contract at a sinusoidally varying rate) and wavelength (because muscles extend and contract at a faster rate in a shorter cycle). Input rates to a microstrip therefore pass through a sinusoidal cycle with the same duration as the cycle of contraction and extension of muscle fibers. Input rates to next-door neighbouring microstrips pass through the same cycle but a small step out of phase. Therefore, at any given moment, input rates to a network also look like a sine wave, or part of a sine wave. This is what Figure 8 shows.

The size of the part of a sine wave represented by input to a network depends on the length of the body that maps to a network. More specifically, it depends on how much of a complete body wave—a full S shape—fits into that length. The top row of panels in Figure 8 shows input to a network from a whole body wave, so that a graph of the mean of input rates to each microstrip looks like a sine wave. The bottom row of panels shows input to a network from a quarter of a body wave, so that the graph of input rates looks like a quarter of a sine wave. Inputs are passed through the network computation to derive output—Figure 8 does not show the steps of the computation, only input and output.

A whole wave (top row) is unreadable—output is the same regardless of cycle phase. But a quarter of a cycle (bottom row) is converted by the network computation into phase and wavelength-sensitive locomotor output, which is timely, coordinated, and precise. We claim that this illustrates in theory that a microzone can ‘read’ the inner-level parallel fiber code without discriminating between signals or patterns, or even between microstrips.

As a footnote, we observe that the weight of influence on motor output of parallel fiber signals arising in more distant microstrips is weaker because parallel fibers form about half as many synapses distally as proximally (Pichitpornchai et al., 1994). This is reflected in a mutual influence of networks that diminishes with distance (in addition to diminishment because they receive less of the same parallel fiber input).

### 11.5 Short-term plasticity may compensate for sensory-motor lag

An argument against motor control by sensory inputs (including proprioception) is that transmission time to and from the cerebellum would be too long. Short-term plasticity (STP) at the Purkinje cell–DCN synapse may compensate for extracerebellar signal transmission times.

Purkinje cell to DCN transmission simultaneously receives opposing effects of short-term plasticity, which both depress (Telgkamp and Raman, 2002; Pedroarena and Schwarz, 2003) and facilitate it (Turecek et al., 2016). When the Purkinje cell firing rate is constant, the strength of inhibition scales linearly with the afferent rate (Turecek et al., 2016; Turecek et al., 2017). However, when the rate changes, there is a delay (of approximately 100 ms (Telgkamp and Raman, 2002)) before the balance readjusts, with the result that DCN behavior is also sensitive to the dynamics of the Purkinje cell rate (Telgkamp and Raman, 2002). At a changing Purkinje cell rate, the overall strength of inhibition at any given moment depends on the balance of the influence of the Purkinje cell rate itself and net STP at that time. The response is sensitive and fast, with DCNs responding ‘to increasing, as well as decreasing, changes in PC [Purkinje cell] firing rate with immediate modification of their output firing’ (Pedroarena and Schwarz, 2003 p.713).

What is the balance, what controls it, and what is it for? During behavior, Purkinje cell firing rates change constantly. Some locomotor cycles resemble a sine wave (Edgley and Lidierth, 1988; Sauerbrei et al., 2015). As a result, the relative weight of influence of short-term depression and facilitation is constantly changing, in a balance that is phase sensitive. At any given time, the amount and direction of net STP depend on the rate of change and the direction of change of the Purkinje cell firing rate. When the rate is falling, the balance of STP favors depression (because the mechanism of depression is slower to adjust to rate dynamics than the mechanism of enhancement), reducing the strength of inhibition of DCNs. When the Purkinje cell firing rate is rising, the balance of STP favors enhancement (for the same reason), increasing the strength of inhibition of DCNs. At any given moment, the strength of the net effect depends on the gradient of the Purkinje cell rate curve at that time.

Put another way, the outcome of the competition is a linear function of the derivative of the Purkinje cell rate (we propose). Therefore, net STP is also a sine wave, because the derivative of a sine wave is another sine wave with the same wavelength, shifted to the left by 90 degrees.

**Figure 9:**
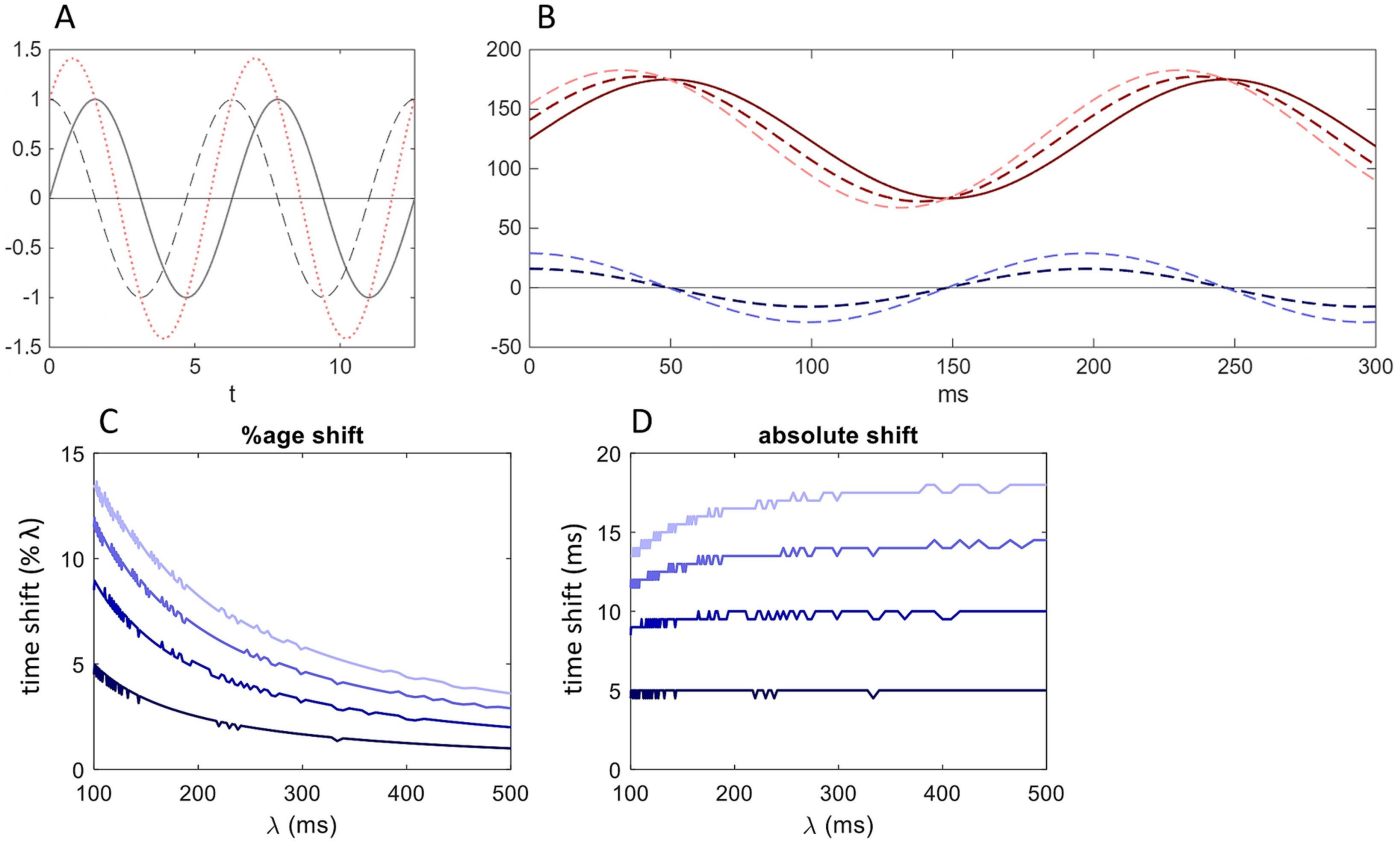
STP time-shifts the cycle of inhibition of DCNs by Purkinje cells. This figure is adapted from Figure 6 in Gilbert and Rasmussen (2025a). We propose that the effect of Purkinje cell–DCN STP is to change the timing of the cycle of inhibition of DCNs. **(A)** Short-term plasticity at the Purkinje cell-DCN synapse simultaneously facilitates and weakens transmission by independent mechanisms. Solid gray line: sine wave representing the component of inhibition of DCNs that is linearly related to the afferent Purkinje cell firing rate in a swimming cycle; dashed gray line: the derivative of the solid gray line, another sine wave phase-advanced by 90 degrees (that is, shifted to the left—‘time shift’), representing the component of inhibition that is the net effect of STP; pink dotted line: the sum of the solid gray line and the dotted gray line, representing net inhibition of DCNs. This panel is purely an illustration. The amount of the time shift depends on the relative amplitude of the rate curve and the STP curve, here the same, so the sum is a 45-degree shift. **(B)** Here, Purkinje cell rate data (solid red line) are all > 0, and STP data (a linear function of the derivative, dashed blue lines) have a smaller range. Dashed dark red line: the sum of rate data and the dashed dark blue line; dashed pale red line: the sum of rate data and the dashed pale blue line. Stronger relative influence of STP (represented by blue amplitude) causes a larger time shift. **(C)** The size of the time shift as a percentage of wavelength. Dark to light blue corresponds to an increasing amplitude of the STP curve. **(D)** The size of the time shift measured in milliseconds, plotted against wavelength. Same color code. A long-standing argument against the participation of sensory-motor arcs in driving motor signals is that motor signals would lag events. We found that the amount of the time shift varies inversely with wavelength measured as a percentage of cycle duration, but that it has an almost unchanging time measured in milliseconds for *λ* > 100 ms, making it suited to compensate for fixed spinal transmission time.

Inhibition of DCNs is the net effect of STP and the absolute Purkinje cell rate. If that is the sum of their influence, the result is a third sine wave somewhere between the first two (because that is the result of summing two sine waves with the same wavelength). This is therefore also shifted to the left of the Purkinje cell firing rate curve, by an amount—that is, by a time—that depends on the relative weight of the influence they each have (Figure 9). The greater the influence of STP relative to the Purkinje cell rate, the larger the time shift, and vice versa.

With this mechanism, the rate of change of the Purkinje cell firing rate—and therefore the relative weight of STP in the balance of influence—is inversely proportional to wavelength. This has the result that there is a larger time shift relative to wavelength if cycle duration is short and a smaller time shift relative to wavelength if cycle duration is long. The result is a functional range where the absolute time shift is fixed (Figure 9).

If this is correct, short-term plasticity is a candidate mechanism to compensate for fixed extracerebellar transmission time, and therefore the elapse of time between conditions that generate sensory feedback (including proprioception) and the effect downstream of motor signals on movement. Time-compensated feedback could contribute to coding and generating motor signals, we propose, so that motor sequences are self-driving with relatively light input from higher brain centers.

## 12 Discussion

### 12.1 Scope

**Figure 10:**
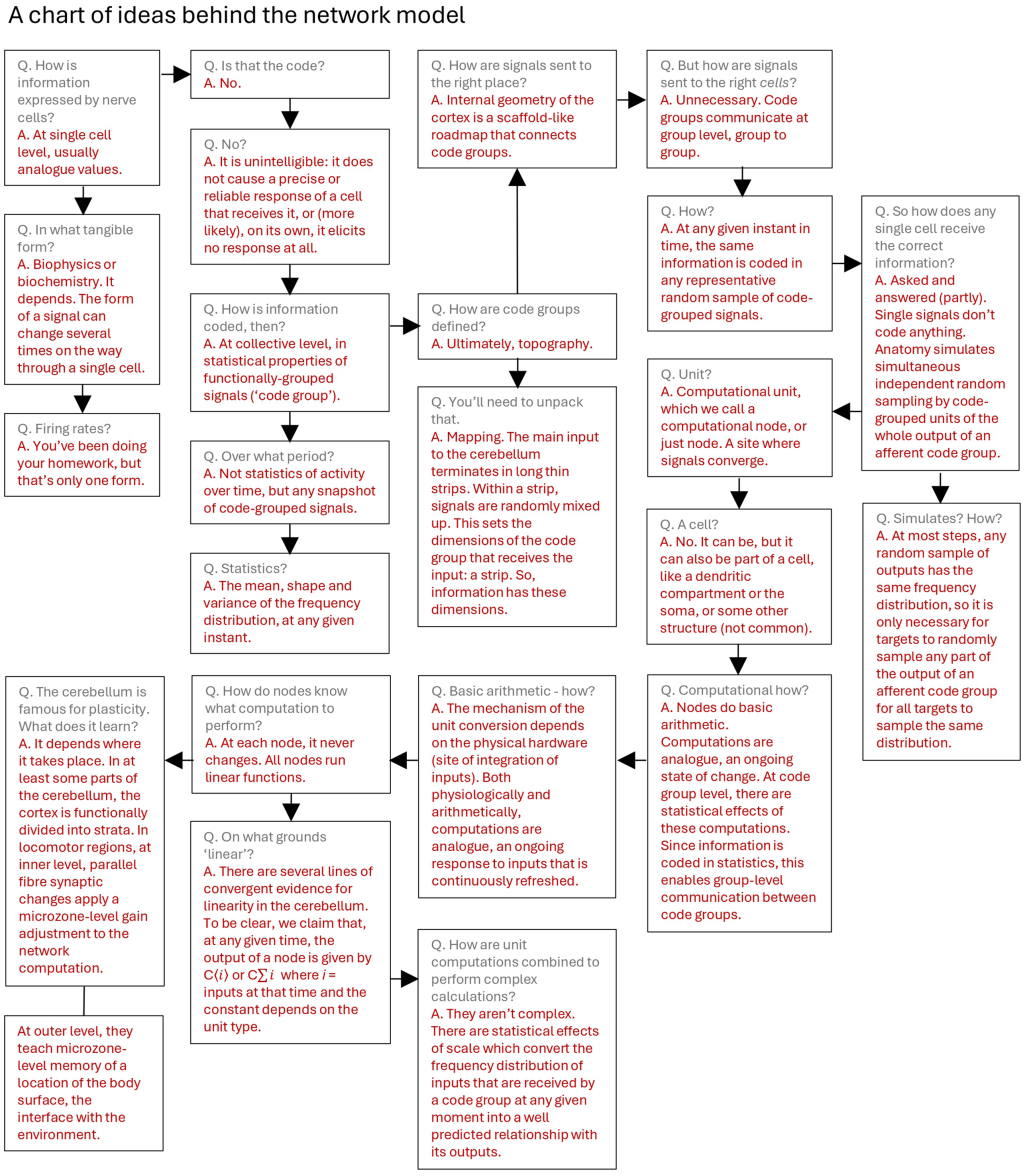
A flow chart of ideas.

We present here what is mostly a summary of the main ideas that have previously appeared in four papers, one each on the inner (Gilbert and Rasmussen, 2025b), middle (Gilbert and Rasmussen, 2025c), and outer (Gilbert and Rasmussen, 2025a) layers of the cerebellar network, and the fourth modeling parallel fiber synaptic learning (Gilbert, 2021) See Figure 10 for a recap.

Our scope is the network computation, and more narrowly, the inner-level computation in a locomotor network. It is likely that different regions of the cerebellar cortex, dedicated to other functions, use the same repeating architecture to process data in different ways, and also that the form and function of memory are different. We do not claim to explain, for example, evidence of blink reflex conditioning or the vestibulo-ocular reflex.

For functional context, we discuss whether proprioception could code kinematics, and this data could be converted by the network computation into phase-coupled motor outputs to power anguilliform swimming, probably the original vertebrate locomotor strategy. Our purpose is to demonstrate that the network computation could, in principle, control complex behavior. We do not address the detailed mechanics of neuromuscular control or land-based locomotion, but make the following remarks.

It is a central question of evolutionary neuroscience how proto-vertebrate locomotion may have first appeared. If skilled movement can be primarily the passive result of neuromuscular hardware, it would be a candidate to explain how the hardware evolved without a plan and without higher brain function yet in place.

The principles that underpin the neuromuscular organization of land animals are likely to have been a later development of the principles that coordinate swimming. Neuroanatomy of the cerebellar cortex is highly conserved between extant vertebrates (Larsell, 1967; Larsell, 1970), suggesting that elements of functional organization may have been retained but adapted to later body plans (and other functions, not just motor control). Candidate early principles may therefore provide a platform to investigate modern systems.

### 12.2 Method

Our methodology is atypical. Normally, neural network models propose a computational mechanism to implement a hypothesized function (function-led); direction: function → model → support (Marr’s three levels of analysis). The function is the justification of the mechanism, but also a premise, and therefore an assumption of the model, with the known downside that if it is wrong, the model falls over. Our approach is in reverse—we infer a mechanism from the evidence and then infer function from the mechanism (evidence-led). In the traditional direction, the model is often an algorithm that solves a problem. With our direction, the computational work is a biologically grounded, physiologically high-resolution reconstruction of a network.

A feature of our model is that individual computational nodes have an identified tangible physical form. Cerebellar neurophysiology is known in detail, so we know how nodes and node groups are connected. A node—we claim—is the physical site and biophysical form of a linear function. We can therefore represent a defined volume of the cerebellar cortex and network function as a set of calculations. In this way, we can represent—as a computer simulation—data processing by complex physiology without simplifying the computation.

Normally, bottom-up mechanistic models have a narrow focus: single cell or biophysics. ‘It is sometimes said that top–down theories are concerned with uncovering computational principles, whereas bottom-up realistic models deal with biological implementations’ (Wang et al., 2020). We do both: we take a bottom-up approach to uncover computational principles via biological implementation.

We do not tune or optimize the model to achieve a desired outcome. Working in this way, we are able to map the transformation of input values to output. There is no explicit overarching mathematical formulation because we claim that abstracting the computation to a formula does not describe cerebellar operations.

### 12.3 Learning

Controversially, we propose that parallel fiber synaptic learning, for years a focus of cerebellar models, has *in its locomotor role* a function that is supplementary to the network conversion (and does not, for example, implement a learning algorithm). That function is a network-level gain adjustment. The adjustment is learned under climbing fiber tuition and acquired and stored separately by each network (as microzone memory). This allows the network computation to adapt to changes or variations in the peripheral hardware of the system, such as changes during growth and development, or physical variation between individuals. It may also bring flexibility that allows basic circuit wiring to be standard and conserved across species (Larsell, 1967; Larsell, 1970).

How does binary synaptic plasticity reconcile with studies showing what seem to be graded changes? Our reading of the literature is that the evidence is inconclusive. Experimental work has not yielded unmixed support for the expected role of long-term depression in motor learning (Welsh et al., 2005; Schonewille et al., 2011; ten Brinke et al., 2015) or for finely graded modification of synaptic weights predicted by learning models (Isope and Barbour, 2002; Ekerot and Jörntell, 2001). There is accumulating evidence of linear transmission of rate-coded data in the cerebellum (Rancz et al., 2007; Ritzau-Jost et al., 2014; Jelitai et al., 2016; Duguid et al., 2015; Duguid et al., 2012; Walter and Khodakhah, 2006; Walter and Khodakhah, 2009), and that contact between cells is adapted for faithful transmission of rate information (Delvendahl and Hallermann, 2016; Saviane and Silver, 2006; Cesana et al., 2010; Moore and Blazis, 1989). Many studies report that cells fire at a rate that is a linear function of behavioral and task metrics ‘at all levels of the cerebellar circuit’ (Raymond and Medina, 2018 p. 239, citing 30 sources). Moreover, neurons seem to be adapted to mitigate biophysical noise, such that non-linear intrinsic properties of neurons and synapses ‘are disengaged or compensated in ways that keep the cerebellar network operating in a linear regime (Alvina et al., 2008; Rössert et al., 2014; Schonewille et al., 2006; Turecek et al., 2017; van Vreeswijk and Sompolinsky, 1996)’ (Raymond and Medina, 2018).

We believe it is unlikely that linear transmission will be reconciled with models that use experience to train synaptic weight adjustments. It is a contentious area, and some of the evidence can be read in more than one way. We think it is a valid position to argue against graded weights and the healthy competition of ideas.

### 12.4 A challenge to core assumptions

Our methodology has led to unexpected conclusions, forcing—we claim—a reappraisal of the principles that underpin the network computation. The proposals are likely to be controversial. We challenge widely made assumptions that do not usually receive explicit scrutiny. We do so by arguing that a different construction of the evidence is possible that does not need to make them. For example, we propose that information is coded in hyper-low resolution (in rate statistics) by code groups that occupy a large volume (long, thin strips). The pattern of active cells (in cases where they are not all active) and the permutation of rates at which they each fire make no difference to the information they code. This is a wholesale departure from traditional learning models, which depend on the idea that the permutation of active cells is code, or part of the code.

The traditional cerebellar model, and learning network models generally, rely on the idea that training by experience teaches changes of synaptic transmission strength in a learned pattern of modified synapses, each finely tuned. Following training, there is an acquired response to signals received at training-modified synapses. In that sense, the system acquires pattern memory. The implementation can be sophisticated, and depends on the particular model, but the rationale (here paraphrased) is uncomplicated.

This proposition depends on several assumptions. These are related, so they can appear to be mutually reinforcing. They are:

- The information coded by a group of cells depends on which cells are active and what rates they each fire at.
- Patterns are reliably replicable.
- Training can teach graded synaptic changes.
- Biological system components have standard and reliable performance characteristics.

We propose instead that:

- Information is coded in statistics of group activity. Groups are topographically defined. Computational nodes are not necessarily cells; units take the physical form of any site where signals converge (for this reason, we use ‘computational nodes’ instead of ‘computational units’).
- Functionally, it is unnecessary for patterns to be reliably (or ever) replicated, and probably they aren’t. Given the known unpredictability of the behavior of neurons and synapses (Faisal et al., 2008), the cerebellum may never receive an exact repeat of the same mossy fiber signals. Moreover, the granular layer of the cerebellar cortex appears to be designed to quasi-randomise internal patterns, so that even minor variation of either active cells or firing rates received as inputs is converted to random decorrelation of the makeup of the subset of active parallel fibers.
- Learning and memory are at microzone scale. Learning is stored as the ratio of working to silent parallel fiber–Purkinje cell synapses; synaptic weights are functionally binary (synapses are either robust transmitters or ‘silent’); and all Purkinje cells in a microzone learn the same ratio.
- Neurons and synapses do not have standard and reliable performance characteristics. This is well known but often disregarded by modeling. In fact (we argue), this is a problem for biological signaling to which the cerebellum is a solution. At each step in transmission through the cerebellum, physiology is designed to isolate the effect of one or sometimes two functional variables (that is, variables that code information) and eliminate or mitigate the effect of all other variables (which would otherwise be deafening noise).

Table 1 lists two further assumptions. One is an expectation that neural networks are highly parameterised non-linear systems. Intuition would suggest that powerful computations are likely to be necessary to achieve complex behavioral control. We propose instead that computations are individually simple linear functions that run in parallel in large numbers with statistical effects at scale.

The other further assumption is that the role of sensory feedback, which by convention embraces proprioception, is limited to performance tuning. We ask whether proprioception contributes to motor signals rather than only refining them. Of course, a contribution could provide performance enhancement. Our proposal is that drive may not be exclusively generated internally by the central nervous system. We envisage that proprioception-driven cerebellar motor control operates alongside executive signals and spinal central pattern generators.

It forms part of our thinking that proprioception should not be classed functionally as sensory. Instead, it is an always-present and internally generated component of motor control rather than reflecting unpredictable features of the terrain or medium. In this way, it is distinct and separate. Signals arising in muscles and joints are able to make movement coordinated and skilled precisely because they are a predictable and reliably generated consequence of movement itself.

### 12.5 Why are there so many granule cells?

A high cost in granule cells for a mathematically unsophisticated transformation is counterintuitive. As a rule of thumb, the costs of a biological system are expected to be optimised. We make these observations.

#### 12.5.1 Cost I: cell numbers

Optimality does not set a ceiling on costs. Costs are justified by behavioral utility. If the utility repays the cost, there is a selection advantage. If there is a viable, cheaper way to achieve the same result, a more expensive theory looks less likely, because a frugal cost–benefit ratio will often be more competitive. However, there are variables that can force up costs in practice, provided the price is still justified by utility. Two of those are the volume of signals traffic the system needs to handle and the selection pressure on speed.

The cerebellum does not work like a central processing unit. To avoid a bottleneck, speed is attained by running computations in parallel (in large numbers). Running computations in parallel is also necessary because computations are analogue—nodes are in constant use, so all tasks need dedicated hardware. Locomotor ‘hardware’ means: a functional set of connected networks, which in our example of anguilliform swimming control a corresponding set of same-sided trunk muscles, which is mirrored contralaterally.

This imposes important functional constraints that increase the cost in building materials and maintenance (although not necessarily energy: see below). For multi-tasking in all possible permutations—anything can run in parallel with anything else—the cost in hardware is set by the number of task-dedicated modules and afferents per module, to preserve the vital capacity to process data at modular level in parallel, so that the cerebellum can perform multiple tasks concurrently.

At bottom, the cost in cerebellar hardware is proportional to the total number of afferents because this is necessary to allow multi-tasking at high speed that is never affected by the volume of traffic. The number of afferents is increased because a single mossy fiber may collateralise to innervate multiple modules. Also, at local level, collaterals give rise to sagittal side branches, and these give rise to terminal branches, each ending in a cluster of terminals. As a result, the cerebellum receives at least scores and may receive hundreds of copies of each signal, proportionally expanding the number of granule cells needed to receive it. There is a linear relationship between the average number of terminals per cluster and the number of granule cells, and also between the average number of terminal branches per mossy fiber and the number of granule cells, other things being equal. Given an average of 7–8 terminals per cluster (Sultan and Heck, 2003) and 4.5 terminal branches per mossy fiber per microstrip (Gilbert and Rasmussen, 2025b), there is a cost of about 97% of the total population of granule cells to implement this strategy.

The cost is inherent in the strategy of collectively coding input data in topographically grouped analogue signals. Given a maximum number of terminals per unit volume, clustering means fewer mossy fibers can terminate in a given volume. If input data are coded in a larger number of signals, inputs must spill into a larger volume. In the cerebellar cortex, the mediolateral dimension is functionally constrained, so spilling is sagittal. That forces terminal branching; otherwise, different locations do not all receive (a representative sample of) the same frequency distribution. The larger the population of signals that code input, the more terminal branches per mossy fiber are necessary, making strips still longer.

Summary: An expensive, computationally unsophisticated mechanism is counterintuitive because there is a presumption that simple sums cannot control complex behavior and also that they should be cheap. We claim to overturn those presumptions. The cell count is justified by behavioral utility because parallel processing (at both modular and sub-modular levels) is necessary for the cerebellum to be able to multitask at behaviorally competitive speed.

#### 12.5.2 Cost II: energy

In mature animals, the main energy costs are involved in signaling (vs. growth and maintenance). These do not depend only on a cell count, but also on several other factors, which can reduce them. The ratio of granule cells to Purkinje cells is at least several hundred to one (well over 1,000:1 in cats) (Palkovits et al., 1971). If we take the ratios of granule cells, Golgi cells, stellate cells, and basket cells to Purkinje cells as 1,000:1, 1:1, 16:1, and 6:1, respectively (Llinás and Negrello, 2015), granule cells make up approximately 97.5% of the principal cerebellar neuron types. If a small percentage of granule cells is active at any time—say 1%—only around 3.5% of the total cell population is active at any time *in active circuits*. Moreover, granule cell total axon length (and therefore signalling costs) are not increased by collaterals. In addition, parallel fibers are very thin, probably as thin as they can be and still transmit signals (Sultan, 2000). Doubling the diameter of an axon quadruples the volume it contains. Therefore, reducing axon diameter reduces the energy needed to pump sodium out and potassium in after an action potential, because less charge is needed to restore resting potential. Finally, granule cells fire in high-frequency bursts. For most of the time a cell is active, only part of the axon is doing work (vs. prolonged signals, where the whole axon is working for the time the cell is active).

#### 12.5.3 Cost III: cell numbers and energy

Networks share most of their granule cells with neighboring networks. The percentage depends on microzone/microstrip dimensions, which are likely to vary regionally. A mid-range estimate of microzone dimensions would mean a network shares as much as >95% of its granular layer cells with each of the two immediately neighboring networks and a linearly declining percentage with more distant neighbors. So, granular layer cell and energy costs of the network computation are shared: there is a large saving because granular layer cells participate in the concurrent computations of multiple networks.

## Data Availability

Publicly available datasets were analyzed in this study. This data can be found here: DOI: https://doi.org/10.25500/edata.bham.00001160; DOI: https://doi.org/10.25500/edata.bham.00001166; DOI: https://doi.org/10.25500/edata.bham.00001167.

## Footnotes

1 The result of taking the mean and taking the sum of sampled values is of course different, but the distribution of the sample sums is linearly related to the sampled distribution.

2 There may also be a third, innermost stratum but, if so, it is not known where input is from.

3 Recurrent network: cells of the same type have input to each other (note: this is not the meaning of recurrent generally used by network modellers). Feed-forward circuit: two cell types sample the same data and one has input to the other (note: circuit in this context simply means pathway).

## Glossary

Circuit: The classic cerebellar circuit (sometimes called a microcircuit) is formed of a microzone, a small group of cells in the inferior olive, and a group of cells in the deep cerebellar nuclei, which contains the main output cells of the cerebellum. The microzone sends an inhibitory projection to the nuclear group, which in turn projects to the olivary group (this projection is also inhibitory), which sends an excitatory projection back to the microzone, terminating as climbing fibers. Circuits are functionally and (with variations—see Supplementary materials SM1) anatomically segregated.
Central limit theorem: Statistical effects of independent random sampling, which reliably relate the sampled distribution to the distribution of the sample means. (The same effects occur if samples are summed.)
Code group: A functional group of computational nodes. Nodes in a code group form a single functional unit. A code group has volume with spatial dimensions. The shape and size of a code group are defined by topography.
DCN: Deep cerebellar neuron: excitatory projection neurons that carry the main output of the cerebellum. The deep cerebellar nuclei also contain other cell types. We specify when we mean other cell types.
Distribution: Frequency distribution, unless it is obvious from the context that it takes another meaning. We use ‘frequency distribution’ and ‘distribution’ interchangeably.
Field: A purely notional division of the granular layer. A field has the average dimensions of a rectangle that encloses a mossy fiber terminal cluster viewed from the cerebellar surface (200 × 150 μm). A sagittal row of 100 fields has the dimensions of a microstrip. We use the notion of fields to quantify granular layer simulations.
Inner and outer levels/strata: Both the granular layer and the molecular layer of the cerebellar cortex are functionally subdivided into inner and outer strata. We use ‘stratum’ and ‘level’ interchangeably. Levels are modally defined: mossy fiber input to the inner level of the granular layer is predominantly from muscles and joints, while signals received at outer level are from receptors at the body surface. Prevalently, the inner level granule cell axon divides in the inner stratum of the molecular layer, and the outer level granule cell axon divides in the outer stratum of the molecular layer, so that modal strata are preserved in the molecular layer.
IPSC: Inhibitory postsynaptic current.
Linear function: An equation where the left and right sides are related by a straight line. We propose that a network is the physical form of a network of linear functions, each a computational node.
Microfile: A mediolateral row of fields. Like fields, microfiles are purely notional. They are simply a modeling aid to quantify parallel fiber activity. Microfiles lie at right angles to microstrips and microzones.
Microstrip: A proposed functional division of the granular layer of the cerebellar cortex. Microstrips mirror microzonal organization but with less clearly defined boundaries (that is, there is more overlap of the cell population of immediately neighboring microstrips than immediately neighboring microzones). Microstrips are defined by their mossy fiber input just as microzones are defined by their climbing fiber input. Unlike purely notional fields, microstrips are functional.
Microzone: Well-established functional divisions of the molecular layer of the cerebellar cortex.
Network: A ‘network’ is formed of a microzone, the region of the underlying granular layer that provides parallel fiber input to it, and the group of DCNs it has output to (see Figure 2 for a schematic). Part of a network, therefore, is also part of an associated circuit. Networks overlap anatomically: closely neighboring networks share most of the same region of the granular layer.
Node (or computational node): A computational unit. Units take the physical form of any site where signals converge, hence ‘nodes’ instead of ‘units’.
Pinceau: The basket cell axon terminates in fine filaments that wrap around the Purkinje cell soma and first axonal segment. Perhaps 60 basket cells converge onto a single Purkinje cell, forming a basket-like mesh. Basket cell–Purkinje cell communication is ephaptic.
Sector: A nominal division of the molecular layer with the same dimensions as a field viewed from the cerebellar surface. Like fields, sectors are purely notional—they are not anatomically or even functionally defined, they are simply a modeling device.
Synaptic ratio: The ratio of working (robustly transmitting) to silent (severely long-term depressed) parallel fiber–Purkinje cell synapses. All Purkinje cells in a microzone have the same synaptic ratio.
Topography: Functionally ordered projections that connect one location or group of cells to another; at long range, also called mapping, but similar organization also exists at short range, and can even exist between code groups that occupy the same location/volume.
Topographically defined: Topography determines the spatial dimensions of code groups. Microzones, for example, are defined by their climbing fiber input from an olivary group. The functional significance of mapping is that it determines how information is routed through a chain of code groups. Information is coded at collective level by the pathways that connect code groups. ‘Functionally defined’ takes the same meaning as ‘topographically defined’.
Working and silent synapses: Robustly transmitting and long-term severely depressed synapses, respectively.
