# Supplementary material for: Beyond synaptic plasticity: a summary of a linear model of the cerebellar locomotor computation
Source: Front Neural Circuits. 2026 May 12;20:1815319. doi: 10.3389/fncir.2026.1815319 (PMC13201492; doi:10.3389/fncir.2026.1815319)
Supplement: Supplementary file 1 [file Data_Sheet_1.DOCX]

**The locomotor cerebellum rethought: a summary of a theory**

SUPPLEMENTARY MATERIALS

**SM1. How closed are closed cerebellar circuits?**

It remains unconfirmed whether the whole of the cerebellum is divided into microzones. Evidence is still limited to only certain regions, such as the C3 [1] and C1 [2] zones. While some aspects of circuit wiring are probably common to all circuits, cerebellar circuits are modified in different regions for different functions [3]. Indeed, there are other ways that the organisation of the cerebellum as a whole can be divided [4-6].

Nonetheless, basic circuit wiring appears to be well preserved (and is also well preserved between taxa [7-10]). Most Purkinje cells that innervate a nuclear group originate in the same microzone or the same functional but dispersed group of microzones which form part of a multizonal circuit [11, 12]. Microzones are defined by their climbing fibre input from a discrete group of cells in the contralateral inferior olive, which give rise to collaterals that terminate in deep nuclei. Olivary cells contact Purkinje cells in the cerebellar cortex with which they co-terminate collaterally in deep nuclei [13] [see also 14, 15]. Collaterals are reciprocated by the inhibitory nucleo-olivary projection [16, 17], forming what are thought to be substantially closed circuits [15, 18-20].

Segregation is not perfect but organisation into circuits is well established. ‘CF [climbing fibre], olivonuclear, and corticonuclear axons project in a map-like fashion: neighbouring neurons in one region (olive, cortex, or deep nucleus) innervate neighbours in the other two regions’ [21 p.14]. In part of the C1 zone, for example, whose output is to the anterior interpositus nucleus and whose circuits control hind limb movements in rats, ‘a fine grain topography exists’ [2 p.16440].

Possibly, this does not hold for all circuits. Purkinje cell termination patterns are less focussed in the fastigial nucleus, for example [22]. Also, output of a microzone may be received by more than one nuclear location [12], probably as the result of Purkinje cell collateralisation [23]. Nonetheless, closed circuit organisation is generally preserved [12].

**SM2. A review of the evidence of linear signalling in the granular layer**

These materials have previously appeared here [24].

**SM2.1 Linear transmission of mossy fibres to Golgi cells**

Golgi cells are large interneurons whose cell bodies and basal dendrites lie in the granular layer. Mossy fibres directly contact Golgi cell basal dendrites. Golgi cell firing frequency increases linearly with the amplitude of depolarising current [25 p.845]. ‘Golgi cells can follow peripheral signals in a continuous fashion, modulating their frequency with the intensity of the stimulus [citing [26, 27]]’ [28]. As a result, during movement, the ‘output of Golgi cells varies smoothly as a function of input.…They are capable of modulating their output to reflect their input with little noise or signal distortion’ [29]. ‘Sensory-evoked Golgi-cell inhibition scales proportionally with the level of mossy fiber excitatory synaptic input’, such that inhibition reliably conveys mossy fibre rate information [30]. On this evidence: mossy fibre rate information is conserved proportionally in Golgi cell firing rates.

Golgi cells extend their basal dendrites into glomeruli [31, 32]. Contact on them by mossy fibres is multi-synaptic [33], contributing to a reliable, rapid (submillisecond) response. The fast rise time and weak distance dependence of the amplitude and timing of Golgi cell EPSCs evoked by mossy fibre signals, suggests that basal dendrites introduce relatively little filtering [34, 35], as expected for large-diameter dendrites. We note that the pause in Golgi cell firing following a discrete stimulus under anaesthesia [30, 36, 37] disappears from Golgi cell recordings during locomotion [27].

During behaviour in freely-moving animals, mossy fibre activity is dense: a high fraction of mossy fibres are active [38]. Both mossy fibres [39-41] and Golgi cells [27] have been reported to fire with a sustained, time-varying signature. In the behaving animal, Golgi cell basal dendritic membrane potential is a continuous variable under modulation by sustained inputs, we submit. As charge transfer to the soma is passive, polarisation of the soma is likewise sustained, under modulation by dendritic states.

It is worth noting that very high mossy fibre rates can be generated in experimental conditions. However, the typical physiological range is 50–300 Hz [40]. In the simulations, we use the physiological rates, within stated constraints on the shape of the distribution.

**Is there other control of Golgi cells?** Do Golgi cells receive a significant influence of input from other sources? The evidence is incomplete but currently suggests other influence is absent or weak. Contrary to early reports, neither Purkinje cells nor climbing fibres contact Golgi cells [42, who give references], and only a modest minority of inhibitory inputs to Golgi cells (if any) are from molecular layer interneurons [43], which generate weak synaptic currents [44], consistent with either extremely weak or wholly absent innervation [45]. There is conflicting evidence whether Golgi cells make inhibitory synaptic contact on each other [45, 46]. Our simulation does not include an effect on Golgi cell firing by other sources of input.

**Golgi cell oscillations.** Golgi cells fire autonomously. Under anaesthesia, firing falls into a slow oscillating pattern [47]. Under excitatory synaptic input, however, this disappears [46]. Anaesthesia ‘has a strong influence on spontaneous activity of Golgi cells’ [42]. Discharge at low rates with irregular timing under anaesthesia [48] is replaced by higher rates with more regular timing without anaesthesia [26, 27, 49]. This paragraph is a cautionary note about evidence obtained under anaesthesia, which has been used to argue that oscillations provide a form of signalling.

**SM2.2 Linear transmission of mossy fibres to granule cells**

There is significant evidence that granule cell firing rates are linearly related to input rates, but also evidence that has been given a conflicting interpretation. We take them in turn.

The short and equal length of granule cell dendrites would suggest light and equal filtering. The mossy fibre-granule cell connection has a range of adaptations which support high-fidelity transmission of high frequency signals across a wide bandwidth [50]. Faithful transmission of rate information is reported [51, 52]. Vesicle release and replenishment are fast [53, 54] and postsynaptic AMPA receptors operate in their linear range [54], where they are resistant to desensitisation [55]. Multiple contacts are made by a mossy fibre with each granule cell [56]. Conversion of depolarising somatic charge into granule cell firing is a simple function, such that granule cells ‘have a relatively linear and uncomplicated conversion of depolarisation level to spike rate’ [57 p.2393, citing Jorntell and Ekerot 2006 and D'Angelo et al 1998].

Glutamate spillover enhances precision and reliability of transmission [54, 58]. During behaviour, at sustained physiological mossy fibre rates, there may be a build-up of intraglomerular glutamate, increasing the relative influence of spillover in a balance with synaptic transmission, assisted by short-term synaptic depression. In these conditions, spillover may dominate, as it dominates inhibitory transmission [59, 60]. Spillover from multiple release sites (200–400 at a single mossy fibre terminal) is plausibly sufficient to mitigate variability of vesicular size, release probability, and the number of release points at synapses made on any individual cell, increasing fidelity and equality of excitation of granule cells.

Against that, there are reported to be heterogeneous mossy fibre-granule cell synaptic weights. Different strength and short-term dynamics of the mossy fibre to granule cell connection have been reported in vestibular slices, with GABA_A_ receptors blocked. The amplitude of the response, measured at the soma, is reported to be statistically correlated to the source of the mossy fibre that is stimulated [61], suggesting that the strength of the connection depends on the source.

However, the presence of weights does not necessarily mean that the function is to modulate firing rates. Rather, the authors suggest that the function may temporally code the source of inputs because different combinations have different response onset times. An alternative, which they also mention, is that some inputs may be in a supporting role, reminiscent of ‘driver’ and ‘modulatory’ signals in the thalamus and cortex [62]. Feasibly, signals in a supporting role must be present for the postsynaptic granule cell to fire, but do not affect the granule cell firing rate.

**SM2.3 Linear transmission of Golgi cells to granule cells**

The granular layer hypothesis includes the proposal that inhibition of granule cells is, at any time, linearly related to the mean firing rate of Golgi cells afferent to each glomerulus. In support, we cite evidence that GABA spillover into the intraglomerular space is proportional to afferent Golgi cell rates and that inhibition of granule cells is almost exclusively mediated by spillover, and therefore linearly reflects the intraglomerular concentration of GABA.

Fine, beaded axon fibres enter glomeruli, where they inhibit granule cells [32]. ‘In the adult rat cerebellum, each granule cell dendrite receives 2.6 ± 0.55 synaptic contacts from Golgi axon terminals’ [63] citing [56].^[[1]](#footnote-1)^ However, the large majority of inhibition (98%) is by spillover [59], where neurotransmitter released into the synaptic cleft spills out into the glomerular space. Golgi cells release GABA, an inhibitory neurotransmitter. This is detected by high-affinity GABA_A_ receptors located perisynaptically and extrasynaptically on granule cells [64, 65]. Even synaptically-received signals have most of their effect (i.e., the large majority of charge transfer is mediated) via spillover.^[[2]](#footnote-2)^

Golgi cells fire [48] at a time-varying rate in the behaving animal, so that a glomerulus receives continuous input. As a result, there is a sustained build-up of glomerular GABA during behaviour [66] at an adjustable concentration controlled by Golgi cell firing rates [60]. Signalling by spillover is sometimes assumed to be ambient and slow. However, the action of glomerular GABA spillover has a fast phasic component – not as fast as synaptic transmission (~1 ms) but with a rise time of only a few milliseconds [60]. Unlike the spiky appearance of synaptically-induced inhibitory postsynaptic currents, spillover [30] generates a sustained outward current.

**SM3. A calculation of the distributed probability of the number of co-active inputs to a stellate cell**

The stellate cell dendritic territory varies with the depth of the cell body in the molecular layer. For superficially located (‘outer-level’) cells it is around 80 x 80 µm. At deeper level (‘inner level’) it is larger, around 120 x 120 µm [33 pp.217-221]. The number of parallel fibres which pass through an inner-level stellate cell territory is given by $a$/(($b$ x $c$)/120^2^), assuming parallel fibres are uniformly distributed, where $a$ is the number that pass through a Purkinje cell territory (350,000 [67]), and $b$ and $c$ are the dimensions of the Purkinje cell territory in the same plane (200 x 300 µm), giving ~84,000. If 0.5% are active in the general population, around 420 of those are active.

It has been estimated that a stellate cell receives contact from several hundred parallel fibres [33, 68], which we take as 1,000. Working with these estimates, 1 in 84 make contact (i.e., 1,000 out of 84,000), so the probability that an active cell makes contact is 1/84 = ~0.0119. Accordingly, contact by $x$ (out of 420) active cells has a probability given by

$$\frac{420!}{x!\left( 420-x \right)!}*{0.0119}^{x}*\left( 1-0.0119 \right)^{420-x}$$

Probabilities are shown in Table A for the range $x$ = 0 to 12, where $x$ is the number of active parallel fibres that make contact and $p$ is the probability of that number. The distribution of probabilities is in good agreement with the modest number – single figures – suggested by ‘two to eight substantial EPSPs’ [69 p.9628].

**Table A** The probability, $p$, that a stellate cell receives contact from $x$ active parallel fibres

| $\boldsymbol{x}$ | **0** | **1** | **2** | **3** | **4** | **5** | **6** | **7** | **8** | **9** | **10** | **11** | **12** |
| --- | --- | --- | --- | --- | --- | --- | --- | --- | --- | --- | --- | --- | --- |
| $\boldsymbol{p}$ | .0066 | .0331 | .0836 | .1403 | .1762 | .1765 | .147 | .1047 | .0651 | .0359 | .0178 | .008 | .0033 |

**Caption.** Distributed probabilities of the number of active inputs to a single stellate cell.

There are theoretical reasons that the proportion of active parallel fibres may be higher than we have assumed here, (perhaps nearer1%). The most important is that the proportion of active granule cells may need to be higher for the number of active cells at local level (that is, in a field) to be large enough to be faithfully representative of the frequency distribution of firing rates in a microstrip.

**SM4. Adaptations for high-fidelity linear Purkinje cell-DCN communication**

**SM4.1 Rate proportional inhibitory charge transfer is reported**

Rate proportional inhibitory charge transfer across the DCN somatic membrane feels counterintuitive because we might expect there would be a mutual effect of current and voltage. However, a linear relationship has been reported. The ‘Purkinje cell-DCN synapse differs from many other synapses in that frequency-independent transmission leads to linear charge transfer, which could encode the absolute rate of Purkinje cell firing more efficiently than a typical depressing synapse’, recording from mouse slices [70, 71].

**SM4.2 Noise cancelling boutons**

We have previously argued that a linear relationship with the afferent spike rate is accomplished by converting each spike to a standard unit of charge transfer, and that contact of Purkinje cells on DCNs is highly adapted for this result [72]. There is a large presynaptic vesicle pool per bouton [73], so there is a reliable result of a low release probability. Neurophysiology mitigates an arbitrary effect of spatial and temporal integration (in addition to the convergence-onto-a-point strategy). The decay constant of inhibitory postsynaptic current is very fast, ~2.5 ms [74, 75], mitigating (i.e., there is modest or no) intrabouton temporal GABA summation of consecutive releases even at high afferent rates. Spatial summation is mitigated because spillover is eliminated or much reduced by specially adapted boutons. Clearance by astrocytic transporters is confined to the bouton perimeter. Otherwise, synaptic signalling would be affected by spillover from closely packed neighbouring synapses.

**SM4.3 Short per spike postsynaptic IPSCs conserves a linear relationship of Purkinje cell signals and postsynaptic current**

Purkinje cell-DCN postsynaptic IPSCs have a short time constant, ~2.5 ms [74, 75]. One might think that a longer time constant would give a more smoothly modulated postsynaptic effect. We have simulated the effect of a longer time constant on postsynaptic current [72 fig 2]. As expected, with a longer time constant there is smoothing on a short (millisecond) time scale. However, there is larger random deviation from a linear relationship with Purkinje cell discharge probability. That is, a longer time constant less faithfully converts the Purkinje cell code (discharge probability) into the signal received by a DCN (the momentary sum of postsynaptic inhibitory current).

**SM5. Basket cells**

The basket cell main axon follows a horizontal course which can extend in either sagittal direction. Basket cells have a longer sagittal range than stellate cells and, unlike stellate cells, their main axon gives rise to horizontal side branches. Basket cell axons ‘may spread over a distance equal to 20 Purkinje cell widths and 6 deep … and may contact as many as 150 Purkinje cell bodies’ [76]. A more conservative estimate is that basket cells contact the territory of 10–12 Purkinje cells sagittally and 3–6 mediolaterally, so around 30–70 Purkinje cells [cats: 77]. As a result there is strong convergence onto Purkinje cells (estimated to be 40–60:1 in cats [78]).

Communication of basket cells with a Purkinje cell is through a pinceau – a mesh of fine terminal axon filaments that enwraps the Purkinje cell soma and first axonal segment, where simple spikes are generated [79, 80]. Like stellate cells, basket cells fire intrinsically. Basket cells are networked with stellate cells and exhibit the same unpredictable spike delays. Accordingly, spike timing of basket cells that form a pinceau is asynchronous. Transmission is exclusively ephaptic and effectively instantaneous [81].

The biophysical effect is to generate a local electric field around the Purkinje cell soma and first axonal segment which is linearly and almost instantaneously responsive to the combined input spike rate (that is, the sum of input spikes to a pinceau per unit time received from afferent basket cells).

We propose a twofold function. First, it stops fluctuation of field potential generated by simple spike discharge falling into an oscillating pattern that aligns spike timing between neighbouring Purkinje cells. Second, it bidirectionally modulates somatic and first axonal segment membrane potential (with a corresponding modulatory effect on the probability of Purkinje cell spike discharge). Communication is fast and noiseless.

Basket cell dendrites lie in the lower two thirds of the molecular layer where, like stellate cells at that level, they receive a constant turnover of active inputs independently sampled from the inner-level parallel fibre distribution. Also like stellate cells, basket cells are connected dendritically by gap junctions (both with each other and stellate cells). Basket cells make more gap junctions per cell, and make connections with a more of their neighbours, than stellate cells [82, 83]. The computational effect of receiving a shifting pattern of randomly sampled inputs, and of gap junctions, is the same as we claim for inner-level stellate cells. The computational effect of spike timing asynchrony and convergence is the same as our equivalent claims for Purkinje cell control of DCNs.

**SM6. Evidence that climbing fibre instruction signals are functionally binary**

The substance of these materials has appeared previously here [84].

**SM6.1 Climbing fibre axonal discharge**

Climbing fibres discharge in a short burst of spikes with what was originally reported to be an all-or-nothing signature [85]. It has been argued that the number of spikes in a burst codes a variable lesson on the basis of a statistical correlation, over many bursts, between the number of spikes and the phase of subthreshold oscillations [86]. A later study reported that there was no correlation with phase but that the number of spikes may code the amplitude of oscillations [87].

Functional significance of a correlation was inferred notwithstanding: i) The maximum functional range of variation was only 3 or 4 spikes; the average number is 2–3 and the range is 1–6 [86, 87]. The number is further reduced because at least 3 spikes are needed to induce LTD [88]. ii) The number of spikes is unpredictable on any given occasion [89]. iii) The intraburst rate is invariable [86, 90]. The “timing of spikes within a burst in the olivary axon is highly stereotyped…with only the number of spikes varying’ [86 p.392]. iv) The number of spikes is unrelated to the strength of stimulation of the inferior olive. ‘The mean number of spikes … [is] independent of the stimulus intensity’ [89 p.201] whether stimulation is only just over threshold or any other strength. v) Research groups reporting a correlation (cited above) made conflicting claims.

Caution was urged even by supporters of a variable lesson. The “number of spikes per CF [climbing fibre] burst was quite variable from one burst to the next and … the changes in burst size for any given situation were small (<1 spike per burst) and could only be detected in the average as a slight probability bias toward generating more bursts with many (>4) or few (1) spikes” [91]. Nonetheless, a variable lesson became what was probably the mainstream view [92-94].

One of the authors has previously argued that the original evidence for a correlation in fact supports the opposite conclusion [84]. Some spikes (generated in the first axonal segment and recorded at the soma) fail to propagate far (>125 μm). “Transmission failure is at random: the first spike always propagates, and the others propagate with variable probability (range *p* = 0.66-0.89 [86]) which depends on their position in a burst…and the original size of the group” [84]. Propagation failure is frequent. For example, an average of 2 out of 3 five-spike bursts are redacted (fig. 1). So, if the number of spikes in a burst codes anything, targets more often than not receive the wrong lesson (and the number does not code stimulus strength received by climbing fibres in the first place [89], since confirmed [95]).

An alternative view is that the number of spikes in a burst codes nothing. In this view, lessons are invariant and instruction signals are functionally binary (on/off).


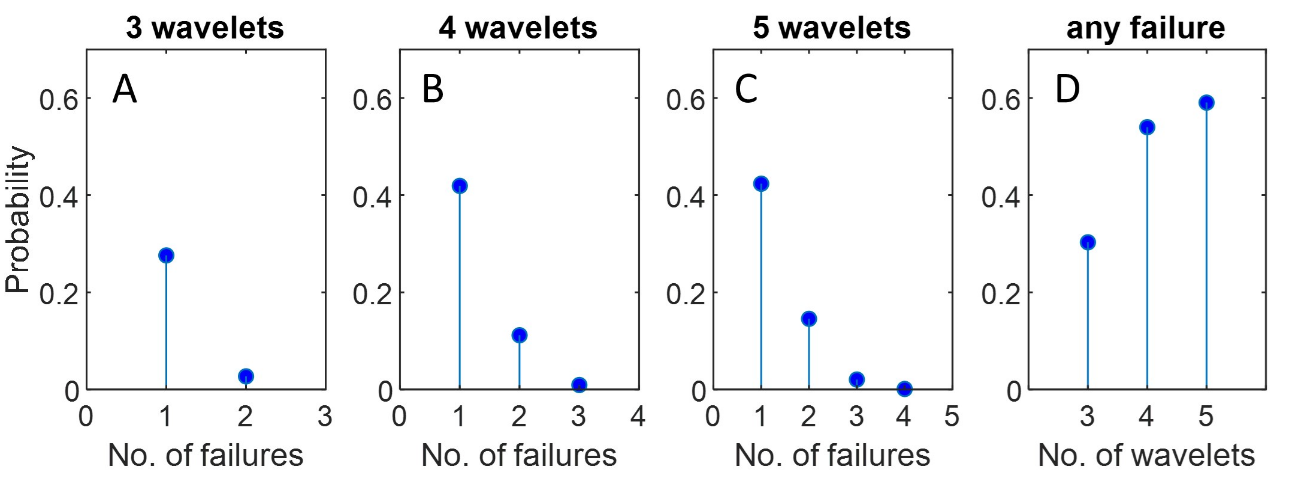


**Fig 1. Spike propagation failures in a climbing fibre burst**

Recorded at the soma, the climbing fibre burst signature is a strong spike followed by a variable number of smaller spikes or wavelets [89, 96, 97]. All are reflected as full spikes in the proximal axon [86]. However, some spikes do not propagate as far as 125 µm. The probability of a failure and of multiple failures depends on the number of spikes in a burst.

**SM6.2 Calcium transients**

SM5 addresses only the form of climbing fibre axonal signals. Signals are relayed to the site of learning – spines on tertiary dendrites where parallel fibres make contact – as dendritic calcium transients. If calcium transients varied functionally, climbing fibre lessons would not be standard.

Climbing fibre calcium transients peak at short latency and reach all parts of the dendritic tree almost simultaneously [98]. While dynamics vary between dendritic branches that have a different diameter and with the local density of calcium channels [99], they do not vary in distal branches that bear spines where parallel fibres make contact, that is to say, at the site of learning. It is rare for the climbing fibre discharge rates to exceed a few Hz [100] and their maximum rate is limited by a lengthy refractory period [101], so in their functional range transients do not overlap temporally, and may not sum anyway: in anaesthetised rats, transients have similar peak amplitude ‘even when the dendritic Ca^2+^ level is already elevated’ [98 p.10849].

There would still be a variable effect of calcium signals if calcium entry triggered by parallel fibre synaptic activation spread so that there was interference between co-active synapses. Spatial or temporal summation of parallel fibre calcium transients would be noise. However, transients are locally confined. Parallel fibres make contact exclusively on spines that spiral around distal dendrites [102], at a single synapse. Calcium entry is locally confined [103]. A number of mechanisms combine to ensure a spine-limited effect [102, 103]. Spines themselves are standard, with a pinched neck, and expressed only by thin tertiary dendrites, so that calcium amplitude and dynamics are not varied by the size or shape of the space it flows into.

**SM6.3 Learning units**

We contend that functionally grouped Purkinje cells receive climbing fibre instruction and learn as a unit. The termination pattern of a climbing fibre is narrowly sagittally constrained. A single climbing fibre contacts 1–10 Purkinje cells at hundreds of synapses each. Accordingly, all targets of a climbing fibre receive the same lesson simultaneously. Microzones are defined by their climbing fibre input [104, 105], which is received from a functionally discrete group of olivary cells. During behaviour, microzone-grouped Purkinje cells receive instruction signals from climbing fibres that discharge as a gap-junction-connected unit in response to sensory stimuli [106]. Synchrony is less evident in resting animals and between spontaneous climbing fibre signals, but synchrony correlations are stronger during locomotion [107]. As a result, microzones receive discharge as a more coherent volley [106]. Therefore, the site of instruction has the dimensions of a microzone.

**SM6.4 Standard dynamics of glutamate spillover from climbing fibre synapses**

If it is correct that evoked climbing fibre signals are functionally binary (SM4), and the climbing fibre calcium transient is not functionally variable (SM6), climbing fibre instruction at the site of learning is functionally invariant at the parallel fibre-Purkinje cell synapses. However, climbing fibres communicate exclusively by glutamate spillover with molecular layer interneurons [108], which express both AMPA receptors and NMDA receptors extrasynaptically.

It forms a proposal of the model that instruction received by interneurons also has a functionally standard signature. The synaptic population which receives a lesson is defined in the same way as the parallel fibre-Purkinje cell synaptic population, by the footprint of climbing fibre termination. Accordingly, the effect of nucleo-olivary feedback has the same spatio-temporal footprint.

As we understand the evidence (discussed in SM4), the number of spikes in a climbing fibre burst is random each time a cell discharges and independent of other cells. A single olivary cell terminates on a variable number of Purkinje cells, up to around 10, in a narrow sagittal strip [109, 110], but on only 1 locally, such that a climbing fibre rarely innervates ‘more than one cell in a field of 30’ [111]. Therefore, signals that converge onto a location are effectively a random sample of a climbing fibre group that reflects the distributed probability of the number of spikes in a burst. If there is an effect of the number of spikes on spillover (not a given), it is mitigated.

Microzone dimensions may assist standardisation between locations in a microzone of the peak amplitude and time course of spillover. Because stellate cells are sagittally flattened and interleaved with Purkinje cells, a whole cell is immersed, at any time, in the same glutamate concentration. As microzones are thin and stellate cells are tightly packed, and glutamate is primarily reabsorbed by astrocytes (rather than dissipating), glutamate concentration is the same at all locations because i) it is not (or is less) affected by a diffusion gradient, and ii) conditions don’t differ between locations (as they would if microzones were irregularly shaped, for example).

Induction of both LTD [112] and LTP [113] at the parallel fibre-stellate cell synapse are calcium dependent. As with Purkinje cells, spatial integration of parallel fibre calcium transients would be noise. AMPA receptors at the parallel fibre-stellate cell synapse lack GluR2 subunits, making them permeable to calcium ([114 p.559], citing [115, 116]). However, postsynaptic diffusion of calcium is severely restricted [112], and the number of co-active inputs to a stellate cell is low.

**SM7. Inner-level stellate cells have bad memory**

**SM7.1 What proportion of parallel fibre-stellate cell synapses are silent?**

An estimated 80–85% of parallel fibre-Purkinje cell synapses are strongly long-term depressed, to the extent that there is ‘no detectable somatic response’ to granule cell stimulation [117 p.9676]. As parallel fibre synapses on Purkinje cells and on stellate cells in the same microzone are trained under instruction of the same climbing fibre signals, we might expect stellate cells would have the same ratio of working (in this section, ‘trained’) to silent (‘untrained’) synapses, but with the sign reversed. For both populations, the total number of trained synapses with $n$ stored patterns is given by

$$x_{n}=x_{n-1}+a\left( \left( m-x_{n-1} \right)/m \right)$$

such that the percentage left untrained is $y_{n}=100\left( 1-\left( {x_{n}}/m \right) \right)$ where $m$ is the total number of parallel fibres which make synaptic contact on the efferent cell (175,000 on a Purkinje cell and some hundreds on a stellate cell) and $a$ is the average number that are active per pattern (a weighted average for stellate cells, derived from a probability distribution). The value of $m$ is an estimate derived from observation and $x_{1}=ar$, where $a=df$ and $r=m/w$. Here, $d$ is the density of parallel fibre activity, $f$ is dendritic field size, and $w$ is the total number of parallel fibres intersecting a field. Field ‘size’ means area in the sagittal plane and the density of active parallel fibres is the number per unit area. The total number of parallel fibres intersecting a field $w$ and field size $f$ are from observation, and density $d$ is an estimate.

As expected, the same proportion of synapses are trained on stellate cells as on Purkinje cells, assuming an even density of parallel fibre activity, calculated in this way. In fact we get the same result for all values of $f$ and $r$ – that is, the result is independent of cell morphology, including depth-dependent morphology of stellate cells.

Note that there is no variable for the number of times a single parallel fibre makes contact on a stellate cell. Contact by a parallel fibre on a Purkinje cell is reported to be at an average of 1.24 synapses, i.e., one and sometimes two [118]. There are no equivalent data for contact on a stellate cell. Contact (if any) made by a passing parallel fibre is assumed to be at a single synapse (flattening of the stellate cell dendritic field is severe and parallel fibres pass through at right angles).

**SM7.2 What weights do working synapses have?**

As far as we know, there is no evidence that parallel fibre-stellate cell synaptic transmission strength is functionally graduated. In an iterative model of parallel fibre synaptic learning under climbing fibre tuition, weights polarise, that is, all synapses either transmit robustly or else transmit very weakly or not at all [119]. Stated very briefly, this is because the learning outcome is itself a parameter of the learning function both at single synapse and collective level.

The result of the reciprocal influence of memory and training is to polarise synaptic weights. The learning outcome is the ratio of silent to functional parallel fibre synapses (‘synaptic ratio’). The unit of memory is a microzone. That is, the ratio is the same for all stellate cells in a microzone, because they all receive the same training. Weights are forced towards the limits of the range because the learning curve is sigmoidal, so the fringes are more stable because learning step size (change per iteration) diminishes towards the limits.

**SM7.3 How, if at all, does inner-level learning affect stellate cell behaviour?**

An unknown pattern of input to a stellate cell may be to all trained synapses, all untrained, or a mixture, with all untrained the least likely (trained and untrained take the same meaning as section 6.1). The probability of $n$ inputs at trained synapses is the probability of $n$ inputs to any synapse (per Table A) reduced by the probability that at least one input is to an untrained synapse (so: the product of *P_n_* and 1 – *P*(none) which becomes *P_n_*(1 – 0.85^n^) below) but increased by the sum of the probabilities that a higher total number of inputs, $a$, is reduced by a number, $b$, to untrained synapses, such that $a - b = n$. For example, the odds that there are two inputs to trained synapses is increased by the product of the probabilities that there are three inputs in total, and any one is to an untrained synapse. It is also increased by the product of the probabilities that there are four inputs, and any two are to untrained synapses, and so on.

So the probability of $n$ inputs at trained synapses with a random pattern is:

$$P_{n}-P_{n}\left( 1-{0.85}^{n} \right)+\sum_{y=1}^{z} P_{n+y}\left[ \frac{\left( n+y \right)!}{y!n!}*{0.15}^{y}*\left( 1-0.15 \right)^{n} \right]$$

where $P_{n}$ is the probability of $n$ inputs (to any synapse, derived in Table A) and $n+z$ is the maximum number of inputs with more than insignificant odds (so around 12 because higher numbers have a very low probability). This gives the Table B probabilities.

**Table B**

| $\boldsymbol{n}$ | **0** | **1** | **2** | **3** | **4** | **5** | **6** | **7** | **8** | **9** | **10** | **11** | **12** |
| --- | --- | --- | --- | --- | --- | --- | --- | --- | --- | --- | --- | --- | --- |
| $\boldsymbol{p}$ | .0086 | .0435 | .1067 | .1715 | .2034 | .1906 | .1472 | .0966 | .0549 | .0276 | .0124 | .0050 | .0019 |

**Caption**. The probability, $p$, that a stellate cell receives contact at trained synapses from $n$ parallel fibres

The Table B probabilities have almost the same distribution as Table A – there are insignificantly different probabilities that signals are received at silent synapses. As synaptic weights are binary, the practical result is that, given our assumptions, training does not materially change stellate cell behaviour at this level. The only difference is a slight probability bias that is undetectable from stellate cell behaviour in any given instance, and averaged out of collective behaviour.

**SM8. Derivation of Golgi cell model parameters**

These materials are adapted from [24]. They are an example of the derivation of parameter values used in our simulations.

Golgi cells are large interneurons whose cell bodies lie in the granular layer. They are most concentrated just below the Purkinje cell layer [33]. The Golgi cell axon branches profusely, giving rise to a dense plexus. The axonal field is sagittally elongated, that is, in the direction of the long axis of a microzone – mean range 650 +/- 179 µm by 180 +/- 40 µm [in mice: 120] – and is the depth of the granular layer, which it fills vertically. Their density [76] and large, profusely ramified axonal field [120] means that Golgi cell axonal territories overlap extensively in the sagittal direction. The Golgi cell population of a row of three fields all inhibit approximately the whole of the middle field. Convergence of their axonal fields defines the size and territory of the smallest functional Golgi cell unit – an ensemble. Their combined range is longer – five fields. But convergence on the middle field is functionally significant because it means all granule cells in the middle field receive inhibition from a random sample of a three-field unit.

There is a significant population of Golgi cells at deeper level in the granular layer. Deeper cells tend to have smaller cell bodies and to lack dendrites that extend into the molecular layer. However, the axons of smaller Golgi cells are indistinguishable from large Golgi cells [33].

4 Purkinje cells fit in a field, based on an estimate of the number that span a microzone [121]. Estimates of the ratio of Golgi cells to Purkinje cells vary. ‘In man, the ratio [of Purkinje cells to Golgi cells] is 1∶1.5, while in the monkey and cat it is almost 1∶1.9 and in the rat 1∶3.3’ [122]. Estimates may be low where Golgi cells were identified by their large size, so that smaller cells at deeper level were not counted [33 p.121]. We take it that there are 10 Golgi cells per field, a ratio of 1:2.5 with 4 Purkinje cells, so 30 in an ensemble.

Contact by mossy fibres on a Golgi cell is on basal dendrites. 4–6 basal dendrites arise from the Golgi cell soma. If we take it as 5, a population of ensemble grouped Golgi cells has a combined total of 30 x 5 = 150 basal dendrites. The number of mossy fibres which contact a Golgi cell is unreported. It has been estimated that a Golgi cell ‘cannot receive more than 10–12 MF [mossy fibre] inputs, given the total basolateral dendrites length and the density of glomeruli’ [42]. We consider two per dendrite (10 between an average of 5 dendrites) is conservative. Dendrites radiate from the cell body. A single dendrite has a range of 50–100 µm [33]. The longest dimension of a field – which contains ~700 mossy fibre terminals – is 200 µm; on the other hand, there is little dendritic branching, so a dendrite occupies a path not a volume. The simulation assumes an average of 4 per dendrite, so 20 per cell.

Golgi cells inhibit granule cells in a structure termed a glomerulus [31-33]. Each mossy fibre terminal is ensheathed in a semi-permeable membrane which restricts neurotransmitter diffusion. Excitation of granule cells by mossy fibres and inhibition by Golgi cells takes place here. Most of the Golgi cell axonal plexus – which is confined to the granular layer – is made up of very fine beaded fibres. These enter glomeruli, where they contact granule cell dendrites [32]. ‘In the adult rat cerebellum, each granule cell dendrite receives 2.6 ± 0.55 synaptic contacts from Golgi axon terminals’ [63] citing [56].^[[3]](#footnote-3)^ However, the large majority of inhibition (98%) is by spillover [59], where neurotransmitter released into the synaptic cleft spills out into the glomerular space. Golgi cells release GABA, an inhibitory neurotransmitter. This is detected by high-affinity GABA_A_ receptors located perisynaptically and extrasynaptically on granule cells [64, 65]. Even synaptically-received signals have most of their effect (i.e., the large majority of charge transfer is mediated) via spillover.^[[4]](#footnote-4)^

As far as we know, inhibition received by a glomerulus is from a random sample of Golgi cells afferent to a field. The convergence ratio – the mean number of Golgi cells with convergent input to a single glomerulus – is unknown, but varies linearly with the divergence ratio, the mean number of glomeruli that receive innervation from a single Golgi cell. The divergence ratio is not known either, but it is high. Each Golgi cell inhibits a large number of granule cells [120, 123]. A single Golgi cell contacts thousands of granule cells [32]. The simulation assumes the convergence ratio is a random variable in the range 8–12:1. That is, we assume each glomerulus receives inhibition from 8–12 Golgi cells, around 1/3 of Golgi cells afferent to a field.

REFERENCES

1. Garwicz, M., H. Jörntell, and C.F. Ekerot, *Cutaneous receptive fields and topography of mossy fibres and climbing fibres projecting to cat cerebellar C3 zone.* J Physiol, 1998. **512 ( Pt 1)**: p. 277-93.

2. Cerminara, N.L., et al., *Structural basis of cerebellar microcircuits in the rat.* J Neurosci, 2013. **33**(42): p. 16427-42.

3. Cerminara, N.L., et al., *Redefining the cerebellar cortex as an assembly of non-uniform Purkinje cell microcircuits.* Nat Rev Neurosci, 2015. **16**(2): p. 79-93.

4. Apps, R. and R. Hawkes, *Cerebellar cortical organization: a one-map hypothesis.* Nat Rev Neurosci, 2009. **10**(9): p. 670-81.

5. Brochu, G., L. Maler, and R. Hawkes, *Zebrin II: a polypeptide antigen expressed selectively by Purkinje cells reveals compartments in rat and fish cerebellum.* J Comp Neurol, 1990. **291**(4): p. 538-52.

6. Wadiche, J.I. and C.E. Jahr, *Patterned expression of Purkinje cell glutamate transporters controls synaptic plasticity.* Nat Neurosci, 2005. **8**(10): p. 1329-34.

7. Bell, C.C., *Evolution of cerebellum-like structures.* Brain Behav Evol, 2002. **59**(5-6): p. 312-26.

8. Bell, C.C., V. Han, and N.B. Sawtell, *Cerebellum-like structures and their implications for cerebellar function.* Annu Rev Neurosci, 2008. **31**: p. 1-24.

9. Larsell, O., *The Comparative Anatomy and Histology of the Cerebellum from Myxinoids through Birds*. 1967: The University of Minnesota Press.

10. Larsell, O., *The Comparative Anatomy and Histology of the Cerebellum from Monotremes through Apes*. 1970: The University of Minnesota Press, Minneapolis.

11. Apps, R. and M. Garwicz, *Anatomical and physiological foundations of cerebellar information processing.* Nat Rev Neurosci, 2005. **6**(4): p. 297-311.

12. Pantò, M.R., et al., *Corticonuclear projections of the cerebellum preserve both anteroposterior and mediolateral pairing patterns.* European Journal of Neuroscience, 2001. **13**(4): p. 694-708.

13. Llinás, R.R., K.D. Walton, and E.J. Lang, *7. Cerebellum*, in *The synaptic organization of the brain*, G.M. Shepherd, Editor. 2004, Oxford University Press: Oxford.

14. Ruigrok, T.J., *Cerebellar nuclei: the olivary connection.* Prog Brain Res, 1997. **114**: p. 167-92.

15. Bengtsson, F. and G. Hesslow, *Cerebellar control of the inferior olive.* Cerebellum, 2006. **5**(1): p. 7-14.

16. Hesslow, G. and M. Ivarsson, *Inhibition of the inferior olive during conditioned responses in the decerebrate ferret.* Exp Brain Res, 1996. **110**(1): p. 36-46.

17. Bengtsson, F., P. Svensson, and G. Hesslow, *Feedback control of Purkinje cell activity by the cerebello-olivary pathway.* Eur J Neurosci, 2004. **20**(11): p. 2999-3005.

18. Shinoda, Y., et al., *The entire trajectory of single climbing and mossy fibers in the cerebellar nuclei and cortex.* Prog Brain Res, 2000. **124**: p. 173-86.

19. Pijpers, A., J. Voogd, and T.J. Ruigrok, *Topography of olivo-cortico-nuclear modules in the intermediate cerebellum of the rat.* J Comp Neurol, 2005. **492**(2): p. 193-213.

20. Fujita, H. and I. Sugihara, *Branching patterns of olivocerebellar axons in relation to the compartmental organization of the cerebellum.* Front Neural Circuits, 2013. **7**: p. 3.

21. Ozden, I., et al., *Widespread state-dependent shifts in cerebellar activity in locomoting mice.* PLoS One, 2012. **7**(8): p. e42650.

22. Sugihara, I., et al., *Projection of reconstructed single Purkinje cell axons in relation to the cortical and nuclear aldolase C compartments of the rat cerebellum.* J Comp Neurol, 2009. **512**(2): p. 282-304.

23. De Zeeuw, C.I., et al., *Projections of individual Purkinje cells of identified zones in the flocculus to the vestibular and cerebellar nuclei in the rabbit.* J Comp Neurol, 1994. **349**(3): p. 428-47.

24. Gilbert, M. and A. Rasmussen, *The cerebellum converts input data into a hyper low-resolution granule cell code with spatial dimensions: a hypothesis.* Royal Society Open Science 2025, in press (an earlier draft is available at bioRxiv DOI: 10.1101/2023.07.14.548987 ver 9, <https://www.biorxiv.org/content/10.1101/2023.07.14.548987v9>).

25. Dieudonné, S., *Submillisecond kinetics and low efficacy of parallel fibre-Golgi cell synaptic currents in the rat cerebellum.* J Physiol, 1998. **510 ( Pt 3)**: p. 845-66.

26. Miles, F.A., et al., *Long-term adaptive changes in primate vestibuloocular reflex. III. Electrophysiological observations in flocculus of normal monkeys.* J Neurophysiol, 1980. **43**(5): p. 1437-76.

27. Edgley, S.A. and M. Lidierth, *The discharges of cerebellar Golgi cells during locomotion in the cat.* J Physiol, 1987. **392**: p. 315-32.

28. Galliano, E., P. Mazzarello, and E. D'Angelo, *Discovery and rediscoveries of Golgi cells.* J Physiol, 2010. **588**(Pt 19): p. 3639-55.

29. Moore, J.W. and D.E.J. Blazis, *11 - Simulation of a Classically Conditioned Response: A Cerebellar Neural Network Implementation of the Sutton–Barto–Desmond Model*, in *Neural Models of Plasticity*, J.H. Byrne and W.O. Berry, Editors. 1989, Academic Press. p. 187-207.

30. Duguid, I., et al., *Control of cerebellar granule cell output by sensory-evoked Golgi cell inhibition.* Proc Natl Acad Sci U S A, 2015. **112**(42): p. 13099-104.

31. Eccles, J.C., R. Llinás, and K. Sasaki, *Golgi cell inhibition in the cerebellar cortex.* Nature, 1964. **204**: p. 1265-6.

32. Hámori, J. and J. Szentágothai, *Participation of Golgi neuron processes in the cerebellar glomeruli: an electron microscope study.* Exp Brain Res, 1966. **2**(1): p. 35-48.

33. Palay, S.L. and V. Chan-Palay, *Cerebellar cortex : cytology and organization*. 1974, Berlin: Springer.

34. Kanichay, R.T. and R.A. Silver, *Synaptic and cellular properties of the feedforward inhibitory circuit within the input layer of the cerebellar cortex.* J Neurosci, 2008. **28**(36): p. 8955-67.

35. Cesana, E., et al. *Novel granule cell-Golgi cell excitatory input in the cerebellar granular layer*. in *FENS Abstr*. 2010.

36. Holtzman, T., et al., *Different responses of rat cerebellar Purkinje cells and Golgi cells evoked by widespread convergent sensory inputs.* J Physiol, 2006. **574**(Pt 2): p. 491-507.

37. Vos, B.P., A. Volny-Luraghi, and E. De Schutter, *Cerebellar Golgi cells in the rat: receptive fields and timing of responses to facial stimulation.* Eur J Neurosci, 1999. **11**(8): p. 2621-34.

38. Hartmann, M.J. and J.M. Bower, *Tactile responses in the granule cell layer of cerebellar folium crus IIa of freely behaving rats.* J Neurosci, 2001. **21**(10): p. 3549-63.

39. Arenz, A., et al., *The contribution of single synapses to sensory representation in vivo.* Science, 2008. **321**(5891): p. 977-80.

40. van Kan, P.L., A.R. Gibson, and J.C. Houk, *Movement-related inputs to intermediate cerebellum of the monkey.* J Neurophysiol, 1993. **69**(1): p. 74-94.

41. Giovannucci, A., et al., *Cerebellar granule cells acquire a widespread predictive feedback signal during motor learning.* Nat Neurosci, 2017.

42. Pietrajtis, K. and S. Dieudonné, *Golgi Neurons*, in *Handbook of the Cerebellum and Cerebellar Disorders*, M. Manto, et al., Editors. 2013, Springer Netherlands: Dordrecht. p. 829-852.

43. Eyre, M.D. and Z. Nusser, *Only a Minority of the Inhibitory Inputs to Cerebellar Golgi Cells Originates from Local GABAergic Cells.* eNeuro, 2016. **3**(2).

44. Dumoulin, A., A. Triller, and S. Dieudonné, *IPSC kinetics at identified GABAergic and mixed GABAergic and glycinergic synapses onto cerebellar Golgi cells.* J Neurosci, 2001. **21**(16): p. 6045-57.

45. Hull, C. and W.G. Regehr, *Identification of an inhibitory circuit that regulates cerebellar Golgi cell activity.* Neuron, 2012. **73**(1): p. 149-58.

46. Vervaeke, K., et al., *Rapid desynchronization of an electrically coupled interneuron network with sparse excitatory synaptic input.* Neuron, 2010. **67**(3): p. 435-51.

47. Ros, H., et al., *Neocortical networks entrain neuronal circuits in cerebellar cortex.* J Neurosci, 2009. **29**(33): p. 10309-20.

48. Ruigrok, T.J., R.A. Hensbroek, and J.I. Simpson, *Spontaneous activity signatures of morphologically identified interneurons in the vestibulocerebellum.* J Neurosci, 2011. **31**(2): p. 712-24.

49. Rasmussen, A., et al., *Golgi cell activity during eyeblink conditioning in decerebrate ferrets.* Cerebellum, 2014. **13**(1): p. 42-5.

50. Delvendahl, I. and S. Hallermann, *The Cerebellar Mossy Fiber Synapse as a Model for High-Frequency Transmission in the Mammalian CNS.* Trends Neurosci, 2016. **39**(11): p. 722-737.

51. Rancz, E.A., et al., *High-fidelity transmission of sensory information by single cerebellar mossy fibre boutons.* Nature, 2007. **450**(7173): p. 1245-8.

52. Ritzau-Jost, A., et al., *Ultrafast action potentials mediate kilohertz signaling at a central synapse.* Neuron, 2014. **84**(1): p. 152-163.

53. Saviane, C. and R.A. Silver, *Fast vesicle reloading and a large pool sustain high bandwidth transmission at a central synapse.* Nature, 2006. **439**(7079): p. 983-7.

54. Sargent, P.B., et al., *Rapid vesicular release, quantal variability, and spillover contribute to the precision and reliability of transmission at a glomerular synapse.* J Neurosci, 2005. **25**(36): p. 8173-87.

55. DiGregorio, D.A., et al., *Desensitization properties of AMPA receptors at the cerebellar mossy fiber granule cell synapse.* J Neurosci, 2007. **27**(31): p. 8344-57.

56. Jakab, R.L. and J. Hámori, *Quantitative morphology and synaptology of cerebellar glomeruli in the rat.* Anat Embryol (Berl), 1988. **179**(1): p. 81-8.

57. Bengtsson, F. and H. Jörntell, *Sensory transmission in cerebellar granule cells relies on similarly coded mossy fiber inputs.* Proc Natl Acad Sci U S A, 2009. **106**(7): p. 2389-94.

58. DiGregorio, D.A., Z. Nusser, and R.A. Silver, *Spillover of glutamate onto synaptic AMPA receptors enhances fast transmission at a cerebellar synapse.* Neuron, 2002. **35**(3): p. 521-33.

59. Duguid, I., et al., *Tonic inhibition enhances fidelity of sensory information transmission in the cerebellar cortex.* J Neurosci, 2012. **32**(32): p. 11132-43.

60. Mapelli, L., S. Solinas, and E. D'Angelo, *Integration and regulation of glomerular inhibition in the cerebellar granular layer circuit.* Front Cell Neurosci, 2014. **8**: p. 55.

61. Chabrol, F.P., et al., *Synaptic diversity enables temporal coding of coincident multisensory inputs in single neurons.* Nat Neurosci, 2015. **18**(5): p. 718-27.

62. Sherman, S.M. and R.W. Guillery, *Distinct functions for direct and transthalamic corticocortical connections.* J Neurophysiol, 2011. **106**(3): p. 1068-77.

63. Rossi, D.J. and M. Hamann, *Spillover-mediated transmission at inhibitory synapses promoted by high affinity alpha6 subunit GABA(A) receptors and glomerular geometry.* Neuron, 1998. **20**(4): p. 783-95.

64. Nusser, Z., W. Sieghart, and P. Somogyi, *Segregation of different GABAA receptors to synaptic and extrasynaptic membranes of cerebellar granule cells.* J Neurosci, 1998. **18**(5): p. 1693-703.

65. Brickley, S.G., S.G. Cull-Candy, and M. Farrant, *Development of a tonic form of synaptic inhibition in rat cerebellar granule cells resulting from persistent activation of GABAA receptors.* J Physiol, 1996. **497 ( Pt 3)**: p. 753-9.

66. Rossi, D.J., M. Hamann, and D. Attwell, *Multiple modes of GABAergic inhibition of rat cerebellar granule cells.* J Physiol, 2003. **548**(Pt 1): p. 97-110.

67. Harvey, R.J. and R.M. Napper, *Quantitative study of granule and Purkinje cells in the cerebellar cortex of the rat.* J Comp Neurol, 1988. **274**(2): p. 151-7.

68. Carter, A.G. and W.G. Regehr, *Quantal events shape cerebellar interneuron firing.* Nat Neurosci, 2002. **5**(12): p. 1309-18.

69. Jörntell, H. and C.F. Ekerot, *Receptive field plasticity profoundly alters the cutaneous parallel fiber synaptic input to cerebellar interneurons in vivo.* J Neurosci, 2003. **23**(29): p. 9620-31.

70. Turecek, J., S.L. Jackman, and W.G. Regehr, *Synaptic Specializations Support Frequency-Independent Purkinje Cell Output from the Cerebellar Cortex.* Cell Reports, 2016. **17**(12): p. 12.

71. Turecek, J., S.L. Jackman, and W.G. Regehr, *Synaptotagmin 7 confers frequency invariance onto specialized depressing synapses.* Nature, 2017. **551**: p. 503.

72. Gilbert, M. and A. Rasmussen, *The cerebellar deep nuclei: a patch for rate codes?* Front Neural Circuits, 2025. **19**: p. 1548123.

73. Pugh, J.R. and I.M. Raman, *Nothing can be coincidence: synaptic inhibition and plasticity in the cerebellar nuclei.* Trends Neurosci, 2009. **32**(3): p. 170-7.

74. Mercer, A.A., et al., *Sex differences in cerebellar synaptic transmission and sex-specific responses to autism-linked Gabrb3 mutations in mice.* eLife, 2016. **5**: p. e07596.

75. Person, A.L. and I.M. Raman, *Purkinje neuron synchrony elicits time-locked spiking in the cerebellar nuclei.* Nature, 2012. **481**(7382): p. 502-505.

76. Llinás, R.R. and M.N. Negrello. *Cerebellum*. Scholarpedia, 10(1):4606 2015 [cited 2017 06/01]; Available from: <http://www.scholarpedia.org/article/Cerebellum>.

77. Eccles, J.C., M. Ito, and J. Szentágothai, *The cerebellum as a neuronal machine*. 1967, Berlin, New York etc.: Springer-Verlag. 335 p.

78. Palkovits, M., P. Magyar, and J. Szentágothai, *Quantitative histological analysis of the cerebellar cortex in the cat. III. Structural organization of the molecular layer.* Brain Research, 1971. **34**(1): p. 1-18.

79. Palmer, L.M., et al., *Initiation of simple and complex spikes in cerebellar Purkinje cells.* The Journal of physiology, 2010. **588**(Pt 10): p. 1709-1717.

80. Foust, A., et al., *Action potentials initiate in the axon initial segment and propagate through axon collaterals reliably in cerebellar Purkinje neurons.* J Neurosci, 2010. **30**(20): p. 6891-902.

81. Blot, A. and B. Barbour, *Ultra-rapid axon-axon ephaptic inhibition of cerebellar Purkinje cells by the pinceau.* Nat Neurosci, 2014. **17**(2): p. 289-95.

82. Kim, J. and G.J. Augustine, *Molecular Layer Interneurons: Key Elements of Cerebellar Network Computation and Behavior.* Neuroscience, 2021. **462**: p. 22-35.

83. Sotelo, C., *Molecular Layer Interneurons of the Cerebellum: Developmental and Morphological Aspects.* The Cerebellum, 2015. **14**(5): p. 534-556.

84. Gilbert, M. and R.C. Miall, *Gating by Functionally Indivisible Cerebellar Circuits: a Hypothesis.* The Cerebellum, 2021. **20**(4): p. 518-532.

85. Eccles, J.C., R. Llinas, and K. Sasaki, *The excitatory synaptic action of climbing fibres on the Purkinje cells of the cerebellum.* J Physiol, 1966. **182**(2): p. 268-96.

86. Mathy, A., et al., *Encoding of oscillations by axonal bursts in inferior olive neurons.* Neuron, 2009. **62**(3): p. 388-99.

87. Bazzigaluppi, P., et al., *Olivary subthreshold oscillations and burst activity revisited.* Front Neural Circuits, 2012. **6**: p. 91.

88. Rasmussen, A., et al., *Number of spikes in climbing fibers determines the direction of cerebellar learning.* J Neurosci, 2013. **33**(33): p. 13436-40.

89. Crill, W.E., *Unitary multiple-spiked responses in cat inferior olive nucleus.* J Neurophysiol, 1970. **33**(2): p. 199-209.

90. Maruta, J., R.A. Hensbroek, and J.I. Simpson, *Intraburst and interburst signaling by climbing fibers.* J Neurosci, 2007. **27**(42): p. 11263-70.

91. Najafi, F. and J.F. Medina, *Beyond "all-or-nothing" climbing fibers: graded representation of teaching signals in Purkinje cells.* Front Neural Circuits, 2013. **7**: p. 115.

92. Hansel, C., *Reading the clock: how Purkinje cells decode the phase of olivary oscillations.* Neuron, 2009. **62**(3): p. 308-9.

93. Rasmussen, A., *Graded error signals in eyeblink conditioning.* Neurobiol Learn Mem, 2019. **170**: p. 107023.

94. Zang, Y. and E. De Schutter, *Climbing Fibers Provide Graded Error Signals in Cerebellar Learning.* Frontiers in Systems Neuroscience, 2019. **13**(46).

95. Öhman, J., et al., *The Effect of Nucleo-Olivary Stimulation on Climbing Fiber EPSPs in Purkinje Cells.* Cerebellum, 2024.

96. Armstrong, B.D. and R.J. Harvey, *Responses in the inferior olive to stimulation of the cerebellar and cerebral cortices in the cat.* J Physiol, 1966. **187**(3): p. 553-74.

97. Armstrong, D.M. and J.A. Rawson, *Activity patterns of cerebellar cortical neurones and climbing fibre afferents in the awake cat.* The Journal of physiology, 1979. **289**: p. 425-448.

98. Kitamura, K. and M. Häusser, *Dendritic calcium signaling triggered by spontaneous and sensory-evoked climbing fiber input to cerebellar Purkinje cells in vivo.* J Neurosci, 2011. **31**(30): p. 10847-58.

99. Indriati, D.W., et al., *Quantitative localization of Cav2.1 (P/Q-type) voltage-dependent calcium channels in Purkinje cells: somatodendritic gradient and distinct somatic coclustering with calcium-activated potassium channels.* J Neurosci, 2013. **33**(8): p. 3668-78.

100. Gibson, A.R., K.M. Horn, and M. Pong, *Activation of climbing fibers.* Cerebellum, 2004. **3**(4): p. 212-21.

101. Lang, E.J., *Excitatory afferent modulation of complex spike synchrony.* Cerebellum, 2003. **2**(3): p. 165-70.

102. O'Brien, J. and N. Unwin, *Organization of spines on the dendrites of Purkinje cells.* Proc Natl Acad Sci U S A, 2006. **103**(5): p. 1575-80.

103. Isope, P., M.E. Hildebrand, and T.P. Snutch, *Contributions of T-type voltage-gated calcium channels to postsynaptic calcium signaling within Purkinje neurons.* Cerebellum, 2012. **11**(3): p. 651-65.

104. Oscarsson, O., *Functional units of the cerebellum - sagittal zones and microzones.* Trends in Neurosciences, 1979. **2**: p. 143-145.

105. Garwicz, M., C.F. Ekerot, and H. Jorntell, *Organizational principles of cerebellar neuronal circuitry.* News in Physiological Sciences, 1998. **13**: p. 28-34.

106. Ozden, I., et al., *Reliable coding emerges from coactivation of climbing fibers in microbands of cerebellar Purkinje neurons.* J Neurosci, 2009. **29**(34): p. 10463-73.

107. De Gruijl, J.R., T.M. Hoogland, and C.I. De Zeeuw, *Behavioral correlates of complex spike synchrony in cerebellar microzones.* J Neurosci, 2014. **34**(27): p. 8937-47.

108. Szapiro, G. and B. Barbour, *Multiple climbing fibers signal to molecular layer interneurons exclusively via glutamate spillover.* Nat Neurosci, 2007. **10**(6): p. 735-42.

109. Sugihara, I., H.S. Wu, and Y. Shinoda, *The entire trajectories of single olivocerebellar axons in the cerebellar cortex and their contribution to Cerebellar compartmentalization.* J Neurosci, 2001. **21**(19): p. 7715-23.

110. Shinoda, Y. and I. Sugihara, *Axonal Trajectories of Single Climbing and Mossy Fiber Neurons in the Cerebellar Cortex and Nucleus*, in *Handbook of the Cerebellum and Cerebellar Disorders*, S.J.D. Manto M., Rossi F., Gruol D.L., Koibuchi N., Editor. 2013, Springer, Dordrecht.

111. Wilson, A.M., et al., *Developmental Rewiring between Cerebellar Climbing Fibers and Purkinje Cells Begins with Positive Feedback Synapse Addition.* Cell Rep, 2019. **29**(9): p. 2849-2861.e6.

112. Soler-Llavina, G.J. and B.L. Sabatini, *Synapse-specific plasticity and compartmentalized signaling in cerebellar stellate cells.* Nat Neurosci, 2006. **9**(6): p. 798-806.

113. Rancillac, A. and F. Crépel, *Synapses between parallel fibres and stellate cells express long-term changes in synaptic efficacy in rat cerebellum.* J Physiol, 2004. **554**(Pt 3): p. 707-20.

114. Liu, S.J., et al., *Long-term synaptic plasticity in cerebellar stellate cells.* Cerebellum (London, England), 2008. **7**(4): p. 559-562.

115. Liu, S.Q. and S.G. Cull-Candy, *Synaptic activity at calcium-permeable AMPA receptors induces a switch in receptor subtype.* Nature, 2000. **405**(6785): p. 454-8.

116. Clark, B.A. and S.G. Cull-Candy, *Activity-dependent recruitment of extrasynaptic NMDA receptor activation at an AMPA receptor-only synapse.* J Neurosci, 2002. **22**(11): p. 4428-36.

117. Isope, P. and B. Barbour, *Properties of unitary granule cell-->Purkinje cell synapses in adult rat cerebellar slices.* J Neurosci, 2002. **22**(22): p. 9668-78.

118. Napper, R.M. and R.J. Harvey, *Number of parallel fiber synapses on an individual Purkinje cell in the cerebellum of the rat.* J Comp Neurol, 1988. **274**(2): p. 168-77.

119. Gilbert, M., *Gating by Memory: a Theory of Learning in the Cerebellum.* The Cerebellum, 2021. **21**(6): p. 926-943.

120. Barmack, N.H. and V. Yakhnitsa, *Functions of interneurons in mouse cerebellum.* J Neurosci, 2008. **28**(5): p. 1140-52.

121. Dean, P., et al., *The cerebellar microcircuit as an adaptive filter: experimental and computational evidence.* Nat Rev Neurosci, 2010. **11**(1): p. 30-43.

122. Lange, W., *Regional differences in the distribution of golgi cells in the cerebellar cortex of man and some other mammals.* Cell Tissue Res, 1974. **153**(2): p. 219-26.

123. D'Angelo, E., et al., *The cerebellar Golgi cell and spatiotemporal organization of granular layer activity.* Front Neural Circuits, 2013. **7**: p. 93.

1. Data are sparse. The original data are an estimate derived from visual inspection of slices thirty years ago so far without later corroboration, except from Rossi and Hamann 1998, who derive a lower estimate but propose the disparity is because their estimate is too low. [↑](#footnote-ref-1)
2. Because of the long duration of IPSCs generated by spillover (recorded in slices), the total charge carried is three times that of IPSCs generated by directly connected terminals. 63. Rossi, D.J. and M. Hamann, *Spillover-mediated transmission at inhibitory synapses promoted by high affinity alpha6 subunit GABA(A) receptors and glomerular geometry.* Neuron, 1998. **20**(4): p. 783-95. [↑](#footnote-ref-2)
3. Data are sparse. The original data are an estimate derived from visual inspection of slices thirty years ago, so far without later corroboration, except from Rossi and Hamann 1998, who derive a lower estimate but propose the disparity is because their estimate is too low. [↑](#footnote-ref-3)
4. Because of the long duration of IPSCs generated by spillover (recorded in slices), the total charge carried is three times that of IPSCs generated by directly connected terminals. 63. Rossi, D.J. and M. Hamann, *Spillover-mediated transmission at inhibitory synapses promoted by high affinity alpha6 subunit GABA(A) receptors and glomerular geometry.* Neuron, 1998. **20**(4): p. 783-95. [↑](#footnote-ref-4)
